# Multi-method genome- and epigenome-wide studies of inflammatory protein levels in healthy older adults

**DOI:** 10.1186/s13073-020-00754-1

**Published:** 2020-07-08

**Authors:** Robert F. Hillary, Daniel Trejo-Banos, Athanasios Kousathanas, Daniel L. McCartney, Sarah E. Harris, Anna J. Stevenson, Marion Patxot, Sven Erik Ojavee, Qian Zhang, David C. Liewald, Craig W. Ritchie, Kathryn L. Evans, Elliot M. Tucker-Drob, Naomi R. Wray, Allan F. McRae, Peter M. Visscher, Ian J. Deary, Matthew R. Robinson, Riccardo E. Marioni

**Affiliations:** 1 Centre for Genomic and Experimental Medicine, Institute of Genetics and Molecular Medicine, University of Edinburgh, Edinburgh, EH4 2XU UK; 2 Department of Computational Biology, University of Lausanne, 1015 Lausanne, Switzerland; 3 Department of Psychology, University of Edinburgh, Edinburgh, EH8 9JZ UK; 4 Lothian Birth Cohorts, University of Edinburgh, Edinburgh, EH8 9JZ UK; 5 Institute for Molecular Bioscience, University of Queensland, Brisbane, Queensland 4072 Australia; 6 Edinburgh Dementia Prevention, Centre for Clinical Brain Sciences, University of Edinburgh, Edinburgh, EH16 4UX UK; 7 Department of Psychology, The University of Texas at Austin, Austin, TX 78712 USA; 8 Population Research Center, The University of Texas at Austin, Austin, TX 78712 USA; 9 Institute of Science and Technology Austria, 3400 Klosterneuburg, Austria

## Abstract

**Background:**

The molecular factors which control circulating levels of inflammatory proteins are not well understood. Furthermore, association studies between molecular probes and human traits are often performed by linear model-based methods which may fail to account for complex structure and interrelationships within molecular datasets.

**Methods:**

In this study, we perform genome- and epigenome-wide association studies (GWAS/EWAS) on the levels of 70 plasma-derived inflammatory protein biomarkers in healthy older adults (Lothian Birth Cohort 1936; *n* = 876; Olink® inflammation panel). We employ a Bayesian framework (BayesR+) which can account for issues pertaining to data structure and unknown confounding variables (with sensitivity analyses using ordinary least squares- (OLS) and mixed model-based approaches).

**Results:**

We identified 13 SNPs associated with 13 proteins (*n* = 1 SNP each) concordant across OLS and Bayesian methods. We identified 3 CpG sites spread across 3 proteins (*n* = 1 CpG each) that were concordant across OLS, mixed-model and Bayesian analyses. Tagged genetic variants accounted for up to 45% of variance in protein levels (for MCP2, 36% of variance alone attributable to 1 polymorphism). Methylation data accounted for up to 46% of variation in protein levels (for CXCL10). Up to 66% of variation in protein levels (for VEGFA) was explained using genetic and epigenetic data combined. We demonstrated putative causal relationships between CD6 and IL18R1 with inflammatory bowel disease and between IL12B and Crohn’s disease.

**Conclusions:**

Our data may aid understanding of the molecular regulation of the circulating inflammatory proteome as well as causal relationships between inflammatory mediators and disease.

## Background

Inflammation represents a concerted cascade of molecular and cellular events to combat infectious pathogens and endogenous insults. Inflammatory proteins are key mediators of defence and repair responses, and tight spatiotemporal regulation of their plasma concentrations permits effective immune activation and resolution [1]. Whereas acute inflammatory states may prompt severe illness and death, absence of resolution precipitates transition from acute to deleterious chronic inflammatory states [2]. Chronic inflammation facilitates the pathogenesis of various disease states, including diabetes, heart disease, stroke and allergic conditions [3]. Furthermore, inflammatory lesions in brain tissue are often associated with, and may contribute to, neurodegeneration and cognitive decline [4]. Globally, 60% of individuals will die as a consequence of a chronic inflammation-associated disease state [5]. Therefore, identifying biological factors which govern inter-individual variation in circulating inflammatory protein levels may allow for better prediction of individual disease risk and prognosis, and inform disease biology.

To date, a number of studies have aimed to characterise genetic factors associated with the levels of single inflammatory proteins or a small number of such proteins, including C-reactive protein, fibrinogen and interleukin-6 [6–25]. These genetic factors are also known as protein quantitative trait loci or pQTLs. Additionally, studies have examined the genetic architecture of panels of proteins, including inflammatory mediators, and have investigated co-regulatory pathways and associations with disease states [26–35]. Instead of using imputed genotype data, Höglund et al. used whole genome sequencing data to carry out genome-wide association studies (GWAS) on the levels of 72 inflammatory proteins. This led to the identification of 18 novel loci that were not identified using genotyped or imputed SNPs [36]. A number of studies have also carried out epigenome-wide association studies (EWAS) on the levels of a small set of inflammatory proteins, including C-reactive protein, interleukins-(1β, 4, 6, 9 and 10), interferon-gamma, transforming growth factor-beta and tumour necrosis factor [37–42]. Zaghlool et al. performed an EWAS of 1123 proteins, which pointed towards networks of chronic low-grade inflammatory biomarkers (*n* = 944 individuals) [43]. In an integrative approach, Ahsan et al. aimed to identify genetic and epigenetic markers associated with protein biomarkers including inflammatory mediators (*n* ≤ 1033 individuals) [44]. No study has modelled GWAS and EWAS both as stand-alone association studies and in a combined analysis in the context of proteomic data. This would allow for the identification of genetic and epigenetic correlates of inflammatory protein levels and for the estimation of variance in protein levels explained by genetic and epigenetic data, considered in isolation but also conditioned on one another to reflect reciprocal influences of these molecular data types. Here, we triangulate results from multiple statistical approaches to provide a robust set of genetic and epigenetic correlates of inflammatory protein levels.

Notably, most studies examining the molecular architecture of human traits have relied on linear model-based methods which examine marker or probe effects marginally [45, 46]. A number of issues may arise when using linear regression-based methods and if these are not addressed in the study design, it may lead to model overfitting and biased estimation of effect sizes. These potential issues include correlation structure within molecular datasets, data structure (i.e. cellular heterogeneity, batch effects) and omitted variable bias [47]. Several approaches have been proposed to address these issues [47–53] and these encompass strategies which permit the joint and conditional estimation of effect sizes whilst accounting for correlations among markers and confounding variables. Here, we consider a Bayesian penalised regression framework termed BayesR+ which was developed to assess genetic and epigenetic architectures of complex traits [54]. In BayesR+, marker effects (SNP or CpG site) can be estimated jointly whilst controlling for data structure and correlations among molecular markers of different types. Indeed, this method permits the estimation of variance explained in the trait by all methylation probes or genetic markers, either separately or together. BayesR+ has been shown to outperform single-probe linear regression and penalised regression approaches, such as ridge and LASSO, in relation to the correlation of estimated effects with true simulated values as well as mean squared errors between true and estimated coefficients for single-probe regression. Additionally, BayesR+ shows a higher correlation between estimated effects for variance explained by genetic and epigenetic markers in phenotypic traits and true simulated values when compared to a mixed model strategy in both sparse and non-sparse marker settings [54].

In the present study, we use the BayesR+ method (and sensitivity analyses using ordinary least squares (OLS) [55, 56] and mixed model methods [57]) to examine both the genetic and epigenetic architectures of 70 blood inflammatory proteins in 876 relatively healthy older adults from the Lothian Birth Cohort 1936 study (mean age 69.8 ± 0.8 years; levels adjusted for age, sex, population structure and array plate). Hereinafter, we refer to the adjusted inflammatory protein levels as protein levels. These proteins are present on the Olink® inflammation panel and comprise a mixture of proteins with defined functions pertinent to human inflammatory pathways as well as putative roles in inflammation-related disease states. We use priors guided by results from previous genome-wide and epigenome-wide studies [54, 58] for the expected variance explained in circulating protein levels by genetic and epigenetic factors. Applying a stringent approach, we only consider markers or probes that were identified across all methods employed as being associated with a given protein (concordantly identified) and integrate multiple levels of ‘omics’ data to investigate mechanisms by which genetic variants may influence protein levels. Finally, we use our GWAS summary data to test for putatively causal relationships between inflammatory protein biomarkers and neurological or inflammatory disease states. Thus, this paper has two major aims. The first aim is to provide robust and novel estimates for the contribution of genetic and epigenetic factors towards inter-individual variation in circulating inflammatory protein concentrations. The relationships between genetic and epigenetic factors with inflammatory proteins levels are modelled both alone and together. The second aim is to provide the first use of multiple statistical methods in performing genome-wide and epigenome-wide association studies of human proteomic data.

## Methods

### The Lothian Birth Cohort 1936

The Lothian Birth Cohort 1936 (LBC1936) study is a longitudinal study of ageing. Cohort members were all born in 1936 and most took part in the Scottish Mental Survey 1947 at age 11 years. Participants who were living mostly within the Edinburgh area were re-contacted approximately 60 years later (*n* = 1091, recruited at mean age 70 years). Recruitment and testing of the LBC1936 cohort have been described previously [59, 60].

### Protein measurements in the Lothian Birth Cohort 1936

Plasma was extracted from 1047 blood samples and collected in lithium heparin tubes at mean age 69.8 ± 0.8 years. Following quality control, 1017 samples remained. Plasma samples were analysed using a 92-plex proximity extension assay (Olink® Bioscience, Uppsala Sweden). One protein from the panel, BDNF, failed quality control and was removed from the study. For a further 21 proteins, over 40% of samples fell below the lowest limit of detection. These proteins were removed from analyses leaving a final set of 70 proteins. The proteins assayed comprise the Olink® inflammatory biomarker panel. Briefly, 1 μL of sample was incubated in the presence of proximity antibody pairs linked to DNA reporter molecules. Upon appropriate antigen-antibody recognition, the DNA tails form an amplicon by proximity extension which is quantified by real-time PCR. Data pre-processing was performed by Olink® using NPX Manager software. Protein levels were transformed by rank-based inverse normalisation and regressed onto age, sex, four genetic principal components of ancestry and array plate. Standardised residuals from these regression models were brought forward for all genetic-protein and epigenetic-protein analyses. Pre-adjusted protein level distributions are presented in Additional file 1. Associations between pre-adjusted protein levels and biological as well as technical covariates are detailed in Additional file 2: Table S1.

### Genome-wide association studies

LBC1936 DNA samples were genotyped at the Edinburgh Clinical Research Facility using the Illumina 610-Quadv1 array (*n* = 1005; mean age 69.6 ± 0.8 years; San Diego). Quality control procedures for genetic data are detailed in Additional file 3.

BayesR+ is a software implemented in C++ for performing Bayesian penalised regression on complex traits [54]. The joint and conditional effects of typed SNPs (*n* = 521,523 variants) on transformed protein levels were examined. The prior distribution is specified as a mixture of Gaussian distributions, corresponding to effect sizes of different magnitude, and a discrete spike at zero which enables the omission of probes and markers with negligible effect on the phenotype. Informed by data from our previous pQTL study [58], mixture variances for genetic data were set to 0.01 and 0.1 for the stand-alone BayesR+ GWAS. In the combined analysis with epigenetic data, owing to the need for the same number of mixture variances for genetic and epigenetic data in the BayesR+ software, mixture variances were set to 0.01, 0.1 and 0.2. Input data were scaled to mean zero and unit variance, and adjusted for age and sex. To obtain estimates of effect sizes, Gibbs sampling was used to sample over the posterior distribution conditional on the input data. The Gibbs algorithm consisted of 10000 samples and 5000 samples of burn-in after which a thinning of 5 samples was utilised to reduce autocorrelation. Genetic markers which exhibited a posterior inclusion probability of ≥ 95% were deemed to be significant.

Details for the OLS regression model approach are outlined in Additional file 3. In the linear method, markers which surpassed a Bonferroni-corrected conditional significance threshold of 7.14 × 10^−10^ (= genome-wide significance 5.0 × 10^−8^/70 phenotypes) were considered. The genome-wide significance level of 5.0 × 10^−8^ was selected as per convention in GWAS studies.

### Epigenome-wide association studies

DNA from whole blood was assessed using the Infinium 450 K methylation array at the Edinburgh Clinical Research Facility (*n* = 876; mean age 69.8 ± 0.8 years). Quality control procedures for methylation data are detailed in Additional file 3.

Using BayesR+, prior mixture variances for methylation data (*n* = 459,309 CpG sites) were set to 0.001, 0.01 and 0.1. Age, sex and Houseman-estimated white blood cell proportions [61] were incorporated as fixed effect covariates. The same settings as in the genetic analyses were applied. Methylation probes which had a posterior inclusion probability of ≥ 95% were deemed to be significant.

Details for the OLS and mixed linear model approaches are outlined in Additional file 3. For these methods, probes which surpassed a Bonferroni-corrected significance threshold of 5.14 × 10^−10^ (= genome-wide significance 3.6 × 10^−8^/70 phenotypes) were deemed to be significant. The genome-wide significance level of 3.6 × 10^−8^ was selected as per the recommendations of Safarri et al. [62].

### Functional annotation of genetic and epigenetic loci

Genetic markers that were independently associated with protein levels were functionally annotated using ANNOVAR [63] and Ensembl genes (build 85) in FUMA (*FU*nctional *M*apping and *A*nnotation) [64]. Epigenetic probes associated with protein levels were annotated using the *IlluminaHumanMethylation450kanno.ilmn12.hg19* package [65].

### Identification of overlap between *cis* pQTLs and *cis* eQTLs

To determine whether pQTL variants may affect protein levels through modulation of gene expression, we cross-referenced *cis* pQTLs with publicly available (and FDR-corrected significant) *cis* expression QTL (eQTL) data from the eQTLGen consortium. Expression QTL data were derived from blood tissue, 85% of samples were derived from whole blood and 15% of samples were derived from peripheral blood mononuclear cell data [66]. For each protein, expression QTLs were also subset to the gene (messenger RNA) encoding the protein of interest.

### Colocalisation

To test whether a sole causal variant might underlie both an eQTL and pQTL association, we performed Bayesian tests of colocalisation using the *coloc* package in R [67]. For each protein of interest, a 200-kb region (upstream and downstream—recommended default setting) surrounding the appropriate pQTL was extracted from our GWAS summary statistics [68]. For each respective protein, the same region was also extracted from eQTLGen summary statistics. Default priors were applied. Summary statistics for all SNPs within these regions were used to determine the posterior probability for five distinct hypotheses: a single causal variant for both traits, no causal variant for either trait, a causal variant for one of the traits (encompassing two hypotheses), or distinct causal variants for the two traits. Posterior probabilities (PP) ≥ 0.95 provided strong evidence in favour of a given hypothesis.

### Pathway enrichment and tissue specificity analyses

Using methylation data, pathway enrichment was assessed among KEGG pathways and Gene Ontology (GO) terms through hypergeometric tests using the *phyper* function in R. All gene symbols from the 450 K array annotation (null set of sites) were converted to Entrez IDs using *biomaRt* [69, 70]. GO terms and their corresponding gene sets were retrieved from the Molecular Signatures Database (MSigDB)-C5 [71]. KEGG pathways were downloaded from the KEGG REST server [72]. Tissue specificity analyses were performed using the GENE2FUNC function in FUMA. Differentially expressed gene sets with Bonferroni-corrected *P* values < 0.05 and an absolute log-fold change of ≥ 0.58 (default settings) were considered to be enriched in a given tissue type (GTEx v7).

### Mendelian randomisation

Two-sample Mendelian randomisation was used to test for putatively causal relationships between (i) the 4 proteins whose pQTLs were previously shown to be associated with human traits, as identified through GWAS Catalog, and the respective traits [73, 74] (http://www.nealelab.is/uk-biobank/); (ii) the 13 proteins which harboured significant pQTLs and Alzheimer’s disease risk [75]; (iii) gene expression and inflammatory protein levels; and (iv) DNA methylation and inflammatory protein levels. Pruned variants (LD *r*^2^ < 0.1) were used as instrumental variables (IV) in MR analyses. In tests where only one independent SNP remained after LD pruning, causal effect estimates were assessed using the Wald ratio test, i.e. a ratio of effect per risk allele on trait to effect per risk allele on protein levels. In tests where multiple independent variants were identified, and if no evidence of directional pleiotropy was present (non-significant MR-Egger intercept), multi-SNP MR was carried out using inverse variance-weighted estimates. Analyses were conducted using MRbase [76]. Further details are provided in Additional file 3.

## Results

### Genome-wide studies of inflammatory protein levels

In a Bayesian penalised regression model (BayesR+), 16 pQTLs were identified for 14 proteins (Additional file 2: Table S2). Thirteen of these 16 pQTLs (*n* = 13 proteins) directly, or through variants in high linkage disequilibrium (LD) *r*^2^ > 0.75, replicated conditionally significant pQTLs from the OLS regression model (Additional file 2: Tables S3-S5; Additional file 3). The correlation structure among these 13 proteins is shown in Additional file 4: Fig. S1.

**Fig. 1:**
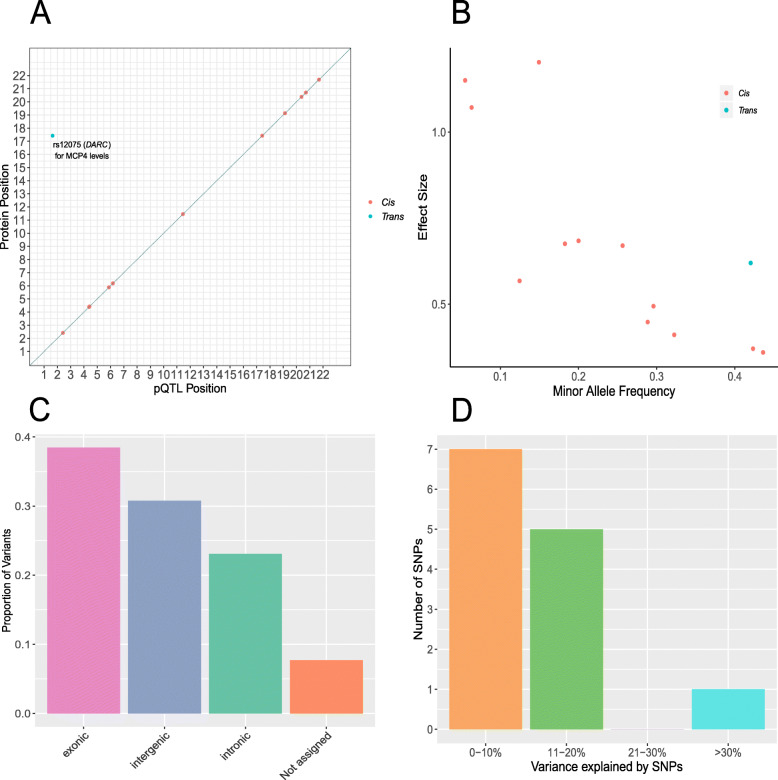
Genetic architecture of inflammatory protein biomarkers in the Lothian Birth Cohort 1936. **a** Chromosomal locations of pQTLs concordant between Bayesian penalised and ordinary least squares regression models for genome-wide association studies (*n* = 13 pQTLs). The *x*-axis represents the chromosomal location of concordantly identified *cis* and *trans* SNPs associated with the levels of Olink® inflammatory proteins. The *y*-axis represents the position of the gene encoding the associated protein. The sole conditionally significant concordant *trans* association is annotated. *Cis* (red circles); *trans* (blue circles). **b** Absolute effect size (per standard deviation of difference in protein level per effect allele) of pQTLs versus minor allele frequency. *Cis* (red circles); *trans* (blue circles). **c** Classification of 13 pQTLs by function as defined by functional enrichment analysis in FUMA. **d** Variance in protein levels explained by pQTLs (estimates from Bayesian penalised regression are displayed)

**Fig. 2:**
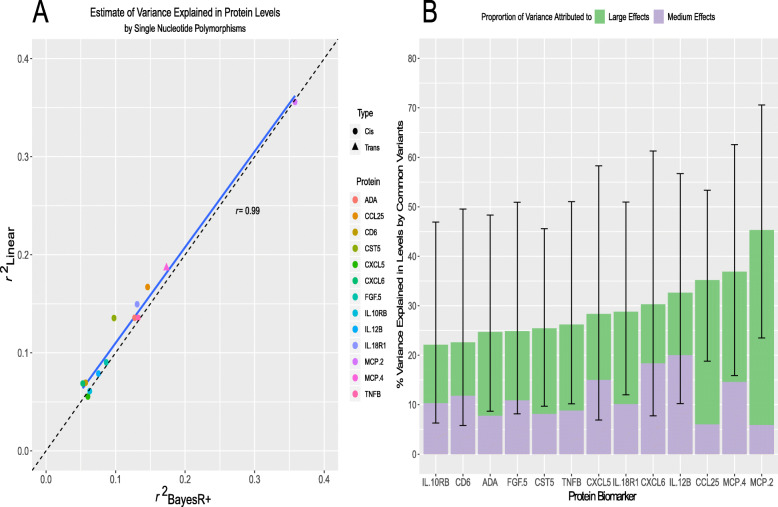
Variance in circulating inflammatory protein levels explained by common genetic variation. **a** In this panel, the variance explained (*r*^2^) by consensus SNPs (*n* = 13 SNP, 1 per protein) in the ordinary least squares regression model was compared against the variance explained by the same SNP set identified in the Bayesian penalised regression approach. **b** The proportion of variance explained in Olink® inflammatory protein levels by common genetic variants genotyped in the LBC1936 participants is shown. Only those proteins which had significant pQTL associations in both the ordinary least squares and Bayesian methods are presented (*n* = 13). Additionally, the proportion of variance explained attributable to medium effects (prior: variance of 1% explained) and large effects (prior: variance of 10% explained) are demonstrated in purple and green, respectively. Error bars represent 95% credible intervals

**Fig. 3:**
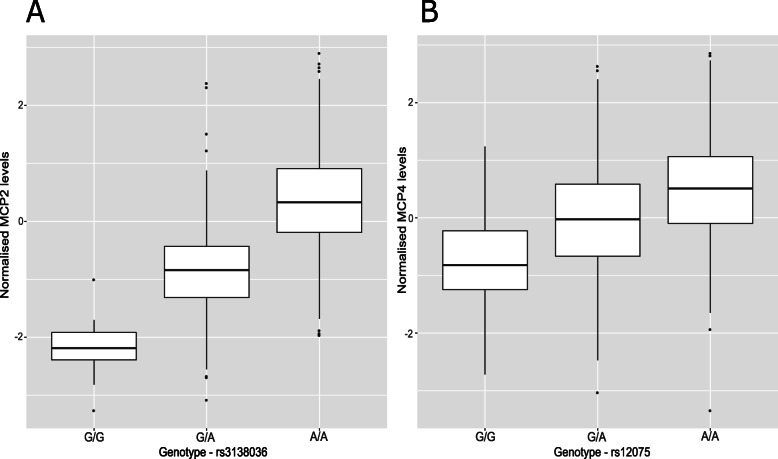
Effect of genetic variation on inflammatory protein levels. **a** Box plot of MCP2 levels as a function of genotype (rs3138036, effect allele: G, other allele: A, beta = − 1.20, se = 0.06). **b** Box plot of MCP4 levels as a function of genotype (rs14075, effect allele: G, other allele: A, beta = − 0.62, se = 0.05). Centre line of boxplot: median, bounds of box: first and third quartiles

Twelve (92.3%) of the concordant SNPs were *cis* pQTLs (SNP within 10 Mb of the transcription start site (TSS) of a given gene [69, 70]) and 1 pQTL (7.7%) was a *trans*-associated variant (Fig. 1a; Additional file 2: Table S6). There was an inverse relationship between the minor allele frequency of variants and their effect size (Fig. 1b). The functional category to which the greatest proportion of variants was assigned was exonic variants (38.5%), as identified by FUMA (*FU*nctional *M*apping and *A*nnotation analysis) (Fig. 1c). Four of the five SNPs annotated to exonic regions produce missense mutations. From the Bayesian model, pQTLs explained between 5.28% (rs10005565; CXCL6) and 35.80% (rs3138036; MCP2) of inter-individual variation in protein levels (Fig. 1d). The estimates for variance accounted for in protein levels by single SNPs were correlated 99% between the BayesR+ and OLS regression models (Fig. 2a; Additional file 2: Table S6). The BayesR+ common (minor allele frequency > 1%) SNP-based heritability estimates ranged from 11.4% (CXCL9; 95% credible interval [0%, 43.5%]) to 45.3% (MCP2; 95% credible interval: [23.5%, 70.6%]), with a mean estimate of 20.2% across the 70 proteins (Additional file 2: Table S7). Figure 2b shows heritability estimates for the 13 proteins exhibiting concordantly identified pQTLs across OLS regression and Bayesian approaches. Figure 3 demonstrates the effect of genetic variation at the most significant *cis* pQTL (rs3138036; MCP2) and the sole *trans* pQTL (rs12075; MCP4) on protein levels.

There was a strong correlation between our SNP-based heritability estimates and those from a previous study of 961 individuals [44]: 29 overlapping proteins, *r* 0.71, 95% CI [0.43, 0.84] (Additional file 2: Table S8 and Additional file 4: Fig. S2).

### Molecular mechanisms underlying pQTLs: colocalisation analysis

Of the 12 *cis* pQTLs which were identified across OLS regression and BayesR+, 8 SNPs (66.67%) previously have been identified as *cis*-acting expression QTLs (eQTLs) in blood (Additional file 2: Table S9). Using *coloc* [67], we tested the hypothesis that one causal variant might underlie both a pQTL and eQTL for each protein. For 4/8 proteins, there was strong evidence (posterior probability (PP) > 0.95) for colocalisation of *cis* pQTLs and *cis* eQTLs (Additional file 2: Table S10). These proteins were CCL25, CD6, CXCL5 and CXCL6.

Mendelian randomisation analyses (MR; see the ‘Methods’ section) indicated that altered gene expression was causally associated with changes in protein levels for each of the four aforementioned proteins (CCL25, CD6, CXCL5 and CXCL6; range of beta [0.68, 12.25], se [0.09, 1.12], *P* [9.54 × 10^−7^, 1.05 × 10^−37^]). However, a second colocalisation approach termed Sherlock [77] suggested that, from the 13 proteins with concordantly identified pQTLs, only expression of *ADA*, *CXCL5* and *IL18R1* were associated with levels of their respective protein products (Additional file 2: Table S11; Additional file 3).

### Epigenome-wide studies of inflammatory protein levels

**Fig. 4:**
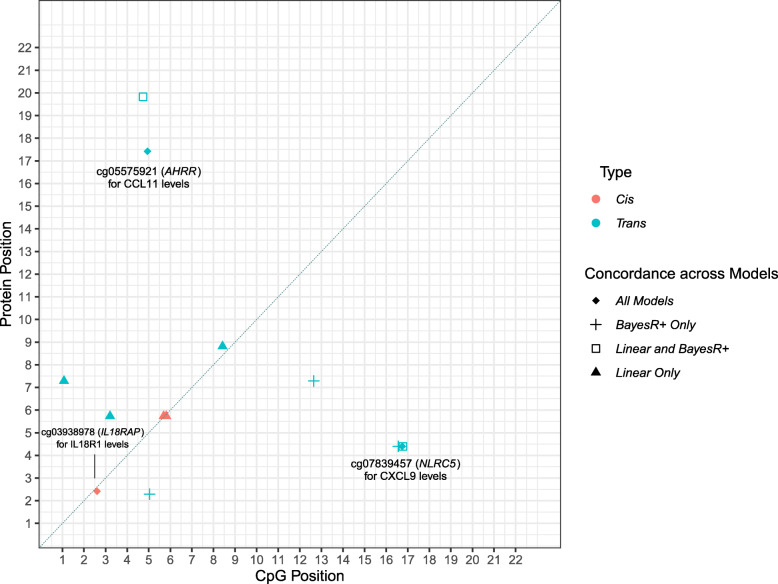
Genomic locations of CpG sites associated with differential inflammatory protein levels. The *x*-axis represents the chromosomal location of CpG sites associated with the levels of Olink® inflammation biomarkers. The *y*-axis represents the position of the gene encoding the associated protein. The level of concordance across three models used to perform epigenome-wide association studies is represented by different shape patterns. Those CpG sites (*n* = 3) which were identified by linear (ordinary least squares), mixed model and Bayesian penalised regression models, and passed a Bonferroni-corrected significance threshold are represented by diamonds and annotated. Three proteins (CXCL9, CXCL10 and CXCL11) were associated with differential methylation levels at the cg07839457 site in the *NLRC5* transcription factor locus. Additionally, two proteins (CCL11 and TGF-alpha) were associated with the smoking-associated cg05575921 site in the *AHRR* locus. *Cis* (red); *trans* (blue)

In the Bayesian model, 8 CpG-protein associations (*n* = 8 proteins) had a posterior inclusion probability of more than 95% (Additional file 2: Table S12). Five of these associations overlapped with those identified by the OLS regression model (*P* < 5.14 × 10^−10^; Additional file 2: Table S13); three of which were also identified in the mixed model approach (*P* < 5.14 × 10^−10^; Additional file 2: Table S14). These were the smoking-associated probe cg05575921 for CCL11 levels (*trans* association at *AHRR*; mixed model—beta − 1.97, se 0.32, *P* 4.86 × 10^−10^), cg07839457 for CXCL9 levels (*trans* association at *NLRC5*; beta − 2.91, se 0.39, *P* 8.03 × 10^−14 ^) and cg03938978 for IL18R1 levels (*cis* association at *IL18RAP*; beta − 1.37, se 0.16, *P* 5.86 × 10^−17^) (Additional file 2: Table S14). Adjustment for smoking attenuated the association between CCL11 levels and the cg05575921 probe (linear model—before adjustment: beta − 1.74, *P* 2.68 × 10^−10^, after adjustment: beta − 1.20, *P* 0.03; % attenuation 31.03%). GWAS and EWAS of CCL11 levels were repeated adjusting for smoking status, the results of the association studies are detailed in Additional file 3. Figure 4 depicts an epigenetic map of CpG-protein associations within this study and demonstrates the degree of overlap between methodologies. The correlation among the three proteins with concordantly identified CpG associations is shown in Additional file 4: Fig. S3. Look-up analyses of the top GWAS and EWAS findings with those reported in the literature are detailed in Additional file 3. For the GWAS, 11/13 pQTLs (84.62%) from the present study were previously reported in the literature. The two loci which represent novel pQTLs are rs11700291 (ADA) and rs1458038 (FGF-5). Beta coefficients displayed a correlation coefficient of 0.88 between those in the present study and those reported in previous studies. For the EWAS, only one of the three concordantly identified CpG-protein associations was previously reported in the literature by Ahsan et al. [44]. This association was between the cg07839457 probe (*NLRC5*) and CXCL9 levels (beta_LBC_ − 2.91 vs. beta_Ahsan_ − 3.26).

We conducted tissue specificity and pathway enrichment analyses based on genes identified by EWAS for each of the 3 proteins with significant CpG associations. Tissue-specific patterns of expression were observed for 2/3 proteins (Additional file 4: Fig. S4-S6). For CCL11, differential expression was observed in breast, adipose and kidney tissue. For IL18R1, differential expression of associated genes was observed in pancreatic tissue. Furthermore, down-regulation of genes associated with IL18R1 was observed in the hippocampus and substantia nigra. There was no significant enrichment of pathways incorporating genes annotated to CXCL9, CCL11 or IL18R1 following multiple testing correction.

One protein, IL18R1, harboured both a significant *cis* pQTL and *cis* CpG site in our study (Additional file 4: Fig. S7). This SNP (rs917997) previously has been identified as a methylation QTL (mQTL) for the single *cis* CpG site associated with IL18R1 levels identified by our epigenome-wide studies (cg03938978) [78]. Using bidirectional MR analysis (Wald ratio test; see methods), we show evidence that DNA methylation at this locus may be causally associated with circulating IL18R1 levels (beta − 0.81, se 0.17, *P* 2.14 × 10^−33^). Conversely, IL18R1 levels may also be causally associated with altered DNA methylation (beta − 1.22, se 0.16, *P* 3.4 × 10^−14^).

**Fig. 5:**
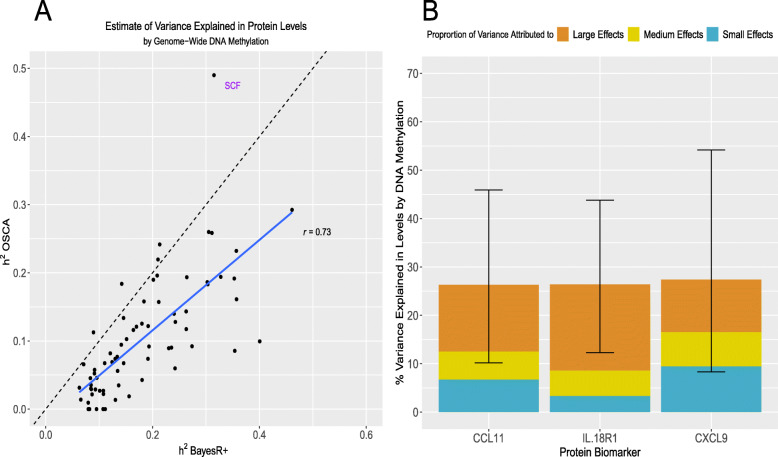
Variance in circulating inflammatory protein levels explained by DNA methylation. **a** In this panel, the variance explained in circulating protein levels by complete methylation data from sites present on the Infinium 450 K methylation array was examined. A comparison between variance explained (h^2^) by a mixed model approach (OSCA) and a Bayesian penalised regression approach (BayesR+) is shown. **b** The proportion of variance explained in Olink® inflammatory protein levels by DNA methylation, as estimated by BayesR+ is shown. Only those proteins (*n* = 3) which had significant CpG associations in ordinary least squares, mixed model and Bayesian methods are presented. Additionally, the proportion of variance explained attributable to small effects (prior: variance of 0.1% explained), medium effects (prior: variance of 1.0% explained) and large effects (prior: variance of 10% explained) are demonstrated in blue, gold and dark orange, respectively. Error bars represent 95% credible intervals

The methylation data explained an average of 18.2% of variance in protein levels using BayesR+; estimates ranged from 6.3% (IL15RA, 95% credible interval [0.0%, 27.3%]) to 46.1% (CXCL10, 95% credible interval [24.1%, 67.1%]) (Additional file 2: Table S15). There was strong concordance with estimates from the mixed model sensitivity analysis (Additional file 2: Table S16 and Fig. 5a). Figure 5b shows the variance explained by methylation data for the 3 proteins exhibiting concordantly identified CpGs across OLS regression, mixed-model and Bayesian approaches.

### Variation in inflammatory protein levels explained by genetics and DNA methylation

When accounting for genetic data, the estimates for variance explained by methylation data were largely unchanged for most proteins (Additional file 2: Table S17; *n* = 9 proteins with change > 5%, 1 with change < − 5% (VEGFA)). The mean absolute change was 2.6% (minimum 0.01% for TNFRSF9 and maximum 15.0% for IL18R1). Similarly, estimates from genetic data were largely unchanged in the combined analysis (*n* = 2 proteins with change > 5%). The mean absolute change was 1.8% (minimum 0.02% for CD244 and maximum 6.7% for CCL28). For 22 proteins, the variance explained by methylation data was greater than that explained by genetic data (Additional file 5).

For each protein, we performed *t*-tests to determine whether the variance explained by methylation or genetic data alone was significantly different from the estimate for variance explained in the combined analysis. For methylation data, 40 proteins showed a significant difference between the estimates for variance in protein levels explained by methylation data alone and methylation data conditional on SNPs (*P* < 0.05). For genetic data, 50 proteins showed a significant difference (*P* < 0.05) (Additional file 2: Table S17).

The combined estimate for variance explained by genetic and methylation data ranged from 23.4% for CXCL1 to 66.4% for VEGFA. The mean and median estimates were 37.7 and 36.0%, respectively. Details of which SNPs and CpGs were identified as being associated with protein levels in the combined BayesR+ analyses, accounting for all genetic and epigenetic factors together, is outlined in Additional file 2: Table S18 and Additional file 3.

### Evaluating causal associations between inflammatory biomarkers and human traits

The 13 independent pQTL associations were queried against GWAS Catalog to identify existing associations between these pQTLs and phenotypes [73]. We investigated whether these associations represented causal relationships. Using two-sample MR, we showed that CD6 levels were causally associated with inflammatory bowel disease (IBD) (beta 0.20, se 0.04, *P* 2.59 × 10^−6^). Furthermore, FGF-5 levels were causally associated with systolic and diastolic blood pressure (beta 0.07 and 0.07, se 0.01 and 0.01, *P* 1.04 × 10^−34^ and 4.29 × 10^−42^, respectively). IL12B levels were associated with Crohn’s disease (beta 0.42, se 0.05, *P* 2.76 × 10^−15^). Circulating IL18R1 levels showed a causal relationship with IBD (beta 0.17, se 0.03, *P* 1.63 × 10^−9^).

Peripheral inflammatory processes and proteins have been linked to risk of late-onset Alzheimer’s disease (AD) [79, 80]. We tested whether the 13 proteins with significant genetic correlates in our study were causally associated with AD risk (Additional file 3). One protein, IL18R1, showed a nominally significant, unidirectional relationship with AD risk (beta 0.02, se 0.01, *P* 0.04) (Additional file 2: Table S19).

## Discussion

Using a Bayesian framework and sensitivity analyses with OLS regression and mixed linear models, we robustly identified 13 independent genetic and 3 epigenetic correlates of circulating inflammatory protein levels. Two of these pQTLs and two CpG sites have not been previously reported as genome-wide significant in the literature. This is the first study to have integrated genetic and epigenetic data together using multiple methods to identify molecular correlates of, and estimate the contribution of these molecular factors towards inter-individual variability in, the circulating proteome. Our results also provide an important and novel demonstration of the overlap between disparate methodologies for performing genome-wide and epigenome-wide association studies on proteomic data. Using integrative causal frameworks, we identified mechanisms through which genetic variation may perturb plasma protein levels. Additionally, we demonstrated causal relationships between prioritised circulating inflammatory proteins and blood pressure as well as inflammatory bowel diseases.

For genome-wide association studies, there is a necessity to perform secondary analyses in order to identify independent loci from association studies. This is often carried out through employing conditional and joint analyses (GCTA-COJO) or LD clumping-based methods, such as those implemented in FUMA [54, 64]. BayesR+ negates the need for such secondary analyses; it allows for the modelling of single marker or probe effects whilst controlling for all other markers or probes. Indeed, BayesR+ can outperform OLS regression or mixed model methods in providing single probe or marker coefficient estimates whilst controlling for all other input SNP and/or CpG sites, as well as known and unknown confounding variables. However, identifying true molecular correlates of protein data over false positive associations is challenging. By relying on careful corrections for multiple testing and triangulation of evidence across disparate methods, our stringent approach was well-equipped to identify likely true biological signal as opposed to false positives.

The issue of identifying true biological signals over false positive associations is particularly pertinent in relation to *trans* associations which show poor replication and often have smaller effect sizes than *cis* associations [81]. We identified one *trans* pQTL (rs12075) associated with levels of the chemokine MCP4 (encoded for by *CCL13* gene on chromosome 17). This SNP represents a nonsynonymous polymorphism (Asp42Gly) annotated to the *Duffy antigen/chemokine receptor* (*DARC*) gene on chromosome 1. Previously, this SNP has been associated with lower MCP1 levels and evidence shows that the base-change results in altered chemokine-receptor binding [10, 20, 82]. Additionally, this polymorphism has been shown to explain approximately 20% of variation in MCP1 levels, similar to our estimate of 18.66% in MCP4 levels [82]. The Duffy antigen receptor is expressed on erythrocytes and acts as a reservoir for circulating chemokines resulting in reduced distribution of chemokines to extravascular tissue and dampened pro-inflammatory effects [83]. Our findings suggest that this polymorphism may also lead to reduced MCP4 levels, possibly through augmented chemokine-receptor interaction.

In the EWAS analyses, the probe cg05575921, located in the *AHRR* locus, was associated with CCL11 levels. This probe is strongly associated with smoking status [84–91] and the association was attenuated after adjustment for smoking. Furthermore, higher levels of CCL11 have been associated with tobacco smoking and cannabis use [92–94]. We also found altered methylation at the *NLRC5* locus (*NOD-like receptor family CARD domain containing 5*) is associated with circulating CXCL9 levels. NLRC5 acts as a potent regulator of the inflammasome [44, 95]. Zaghlool et al. showed that altered methylation at the *NLRC5* locus associates with several inflammatory markers, including CXCL10 and CXCL11, with pathway analyses linking it to disease states in which NLRC5 dysfunction is implicated such as cancer and cardiovascular disease [43].

Using our database of genotype-protein associations, we tested for causal relationships between inflammatory protein biomarkers and human phenotypes. However, in each case, only one variant was available to test for such associations which does not allow for the testing of pleiotropic effects. CD6 was associated with clinically diagnosed IBD. Expression of the CD6 receptor and its ligand, ALCAM, are overexpressed in the intestinal mucosa of IBD patients where it may promote CD4^+^ T cell proliferation and differentiation into pro-inflammatory Th1/Th17 cells [96]. FGF-5 levels were associated with automated readings of systolic and diastolic pressure; previously, FGF-5 levels have been significantly correlated with blood pressure [97]. Variation in the *IL12B* gene has been linked strongly to the pathogenesis of Crohn’s disease and an antibody targeted towards the p40 subunit of IL12 demonstrated efficacy in the treatment of moderate-to-severe Crohn’s disease [98]. In our study, we showed that circulating IL12B levels may be causally linked to this disease. Lastly, IL18R1 levels may also be causally associated with IBD. A number of studies have demonstrated that increased IL18 signalling confers detrimental effects in the context of gastrointestinal inflammatory processes [99].

Our study has a number of caveats. First, proteins with high sequence homology and structural similarities to a targeted protein of interest may be inappropriately captured by assay probes resulting in quantification errors. Olink®’s Proximity Extension Assay technology uses a matched pair of antibodies, coupled to unique, partially complementary oligonucleotides resulting in exceptional readout specificity and greatly reducing this problem compared to other immunoassays. Second, there was a strong correlation structure among the inflammatory protein panel. However, given that inflammatory proteins are often co-expressed and synergistic, overlapping loci may reveal biologically important foci or nodes of co-regulation [100]. Third, functional enrichment analyses indicated that four robustly identified pQTL signals reflect missense mutations in their protein products, three of which were *cis* associations with proteins present on the Olink® inflammation panel. This may lead to altered structural properties of the protein target, thereby affecting antibody-antigen recognition and the ability of assays to accurately quantify protein levels. It is possible that the variants identified may not reflect variants causally associated with blood protein levels, and instead capture a causal variant in the locus. Nevertheless, the identification of such potential protein-altering variants is an important technical consideration in studies aiming to determine the molecular architecture of the human proteome. Furthermore, these variants reflect important candidates for functional characterisation in in vitro studies which aim to dissect their influence on protein abundance in cellular systems. Fourth, our Scottish cohort contains individuals from a homogenous genetic background limiting the generalisability of our findings to individuals of other ethnic backgrounds. Fifth, ageing is closely linked to chronic low-grade inflammation. Therefore, the distributions of, and correlation structure among, inflammatory protein biomarkers may differ in our cohort of healthy older ageing when compared to other age ranges and the general older adult population. Sixth, the sample size within our study resulted in large confidence and credible intervals in the reported estimates for heritabilities in inflammatory protein levels.

## Conclusions

Our integrative and multi-method approach has identified high-confidence genetic and epigenetic loci associated with inflammatory protein biomarker levels. Furthermore, we have provided novel estimates for the contribution of common genetic and epigenetic variation towards differences in circulating inflammatory biomarker levels, considered alone and together. Together, our data may have important implications for informing the molecular regulation of the human proteome. Our data provides a platform upon which other researchers may investigate relationships between inflammatory biomarkers and disease, and a resource to further inform biological insights into immunological and inflammatory processes.

## Supplementary information

**Additional file 1.** Distribution of raw values for inflammatory protein levels across individuals in Lothian Birth Cohort 1936.

**Additional file 2: Supplementary Tables.** The association of pre-adjusted protein levels with biological and technical covariates. Protein levels were adjusted for age, sex, array plate and four genetic principal components (population structure) prior to analyses. Significant associations are emboldened. (**Table S1**). pQTLs associated with inflammatory biomarker levels from Bayesian penalised regression model (Posterior Inclusion Probability > 95%). (**Table S2**). All pQTLs associated with inflammatory biomarker levels from ordinary least squares regression model (P < 7.14 × 10^− 10^). (**Table S3**). Summary of lambda values relating to ordinary least squares GWAS and EWAS performed on inflammatory protein levels (n = 70) in Lothian Birth Cohort 1936 study. (**Table S4**). Conditionally significant pQTLs associated with inflammatory biomarker levels from ordinary least squares regression model (P < 7.14 × 10^− 10^). (**Table S5**). Comparison of variance explained by ordinary least squares and Bayesian penalised regression models for concordantly identified SNPs. (**Table S6**). Estimate of heritability for blood protein levels as well as proportion of variance explained attributable to different prior mixtures. (**Table S7**). Comparison of heritability estimates from Ahsan et al. (maximum likelihood) and Hillary et al. (Bayesian penalised regression). (**Table S8**). List of concordant SNPs identified by linear model and Bayesian penalised regression and whether they have been previously identified as eQTLs. (Table S9). Bayesian tests of colocalisation for *cis* pQTLs and *cis* eQTLs. (**Table S10**). Sherlock algorithm: Genes whose expression are putatively associated with circulating inflammatory proteins that harbour pQTLs. (**Table S11**). CpGs associated with inflammatory protein biomarkers as identified by Bayesian model (Bayesian model; Posterior Inclusion Probability > 95%). (**Table S12**). CpGs associated with inflammatory protein biomarkers as identified by linear model (*limma*) at P < 5.14 × 10^− 10^. (Table S13). CpGs associated with inflammatory protein biomarkers as identified by mixed linear model (OSCA) at P < 5.14 × 10^− 10^. (**Table S14**). Estimate of variance explained for blood protein levels by DNA methylation as well as proportion of explained attributable to different prior mixtures - BayesR+. (**Table S15**). Comparison of variance in protein levels explained by genome-wide DNA methylation data by mixed linear model (OSCA) and Bayesian penalised regression model (BayesR+). (**Table S16**). Variance in circulating inflammatory protein biomarker levels explained by common genetic and methylation data (joint and conditional estimates from BayesR+). Ordered by combined variance explained by genetic and epigenetic data - smallest to largest. Significant results from t-tests comparing distributions for variance explained by methylation or genetics alone versus combined estimate are emboldened. (**Table S17**). Genetic and epigenetic factors identified by BayesR+ when conditioning on all SNPs and CpGs together. (**Table S18**). Mendelian Randomisation analyses to assess whether proteins with concordantly identified genetic signals are causally associated with Alzheimer’s disease risk. (**Table S19**).

**Additional file 3.** Details of Supplementary Methods. Contains information for the following data: Conditional and joint analysis from ordinary least squares GWAS on protein levels; Sherlock: identifying genes whose expression associates with inflammatory biomarkers; GWAS and EWAS of CCL11 levels – incorporating smoking status as a covariate; Replication of previous pQTLs and protein associated-CpG sites; BayesR+ combined analysis – GWAS and EWAS modelled together; Evaluating causal associations between blood inflammatory proteins and Alzheimer’s risk.

**Additional file 4: Supplementary Figures.** Correlation between the 13 proteins with significant pQTLs as identified by ordinary least squares and Bayesian penalised regression. (**Figure S1**). Correlation between heritability estimates for circulating inflammatory protein biomarkers from present study and that of Ahsan et al. The protein with the greatest discordance between studies (MMP-1) is annotated. (**Figure S2**). Correlation between the 3 proteins with significant CpG associations as identified across ordinary least squares model, mixed model and Bayesian penalised regression approaches. (**Figure S3**). Tissue-specific expression of genes annotated to CpGs associated with CCL11 levels at P < 1 × 10^− 5^. Differential expression was observed in kidney, adipose and breast tissue. (**Figure S4**). Tissue-specific expression of genes annotated to CpGs associated with IL18R1 levels at P < 1 × 10^− 5^. Differential expression was observed in pancreatic, hippocampal and substantia nigra tissue. (**Figure S5**). Tissue-specific expression of genes annotated to CpGs associated with CXCL9 levels at P < 1 × 10–5. No tissue-specific expression was observed. (**Figure S6**). Miami plot for IL18R1 which exhibited both genome-wide significant SNP and genome-wide significant CpG associations. The top half of the plot (skyline) shows the results from the GWAS on protein levels, whereas the bottom half (waterfront) shows the results from the EWAS. IL18R1 (chromosome 2: 102,311,529-102,398,775). (**Figure S7**).

**Additional file 5.** Variance in circulating protein levels explained by common genetic and methylation data together.

## Data Availability

Lothian Birth Cohort 1936 data are available on request from the Lothian Birth Cohort Study, Centre for Cognitive Ageing and Cognitive Epidemiology, University of Edinburgh. Lothian Birth Cohort 1936 data are not publicly available due to them containing information that could compromise participant consent and confidentiality. Full and openly accessible summary statistics from the association studies on Olink® inflammatory protein levels are available on the University of Edinburgh Datashare site (https://datashare.is.ed.ac.uk/). These data pertain to summary statistics for GWAS (performed by two methods) and EWAS (performed by three methods) on the levels of 70 inflammatory proteins measured in members of the Lothian Birth Cohort 1936. For OLS regression GWAS data, see https://datashare.is.ed.ac.uk/handle/10283/3624; 10.7488/ds/2814 [101]. For BayesR+ GWAS data, see https://datashare.is.ed.ac.uk/handle/10283/3673; 10.7488/ds/2854 [102]. For OLS regression EWAS data, see https://datashare.is.ed.ac.uk/handle/10283/3628, 10.7488/ds/2818 [103]. For OSCA EWAS data, see https://datashare.is.ed.ac.uk/handle/10283/3627, 10.7488/ds/2817 [104]. For BayesR+ EWAS data, see https://datashare.is.ed.ac.uk/handle/10283/3626; 10.7488/ds/2816 [105]. Summary statistics for the OLS GWAS data are also available at GWAS Catalog (https://www.ebi.ac.uk/gwas/; Study Accessions: GCST90000437-GCST90000506) [106].
