# Supplementary material for: Multi-method genome- and epigenome-wide studies of inflammatory protein levels in healthy older adults
Source: Genome Med. 2020 Jul 8;12:60. doi: 10.1186/s13073-020-00754-1 (PMC7346642; doi:10.1186/s13073-020-00754-1)
Supplement: Supplementary file 3 — Additional file 3. Details of Supplementary Methods. Contains information for the following data: Conditional and joint analysis from ordinary least squares GWAS on protein levels; Sherlock: identifying genes whose expression associates with inflammatory biomarkers; GWAS and EWAS of CCL11 levels – incorporating smoking status as a covariate; Replication of previous pQTLs and protein associated-CpG sites; BayesR+ combined analysis – GWAS and EWAS modelled together; Evaluating causal associations between blood inflammatory proteins and Alzheimer’s risk. [file 13073_2020_754_MOESM3_ESM.pdf]

Quality control of genetic data in Lothian Birth Cohort 1936

SNPs were imputed to the 1000 G reference panel (phase 1, version 3). Individuals were excluded on the basis of sex discrepancies, relatedness, SNP call rate of less than 0.95 and evidence of non-Caucasian descent. SNPs with a call rate of greater than 0.98, minor allele frequency in excess of 0.01 and Hardy-Weinberg equilibrium test with $P \geq 0.001$ were included in analyses.

Genome-wide association studies

Ordinary least squares regression model

The computational strategy for GWAS/EWAS analyses are shown in Fig. 1. Genome-wide association analyses were conducted on 8675776 (typed and imputed) autosomal variants against protein phenotypes which were pre-corrected for age, sex, four genetic principal components of ancestry and Olink® array plate. Ordinary least squares regression was used to assess the effect of each genetic variant on transformed protein levels using mach2qtl (1, 2).

To identify independent genetic associations with Olink® inflammatory levels, we performed approximate genome-wide stepwise conditional analysis through GCTA-COJO (3). We used the 'cojo-slct' option and individual level genotype data. Default settings of the software were applied.

Quality control of methylation data in Lothian Birth Cohort 1936

Raw intensity data were background-corrected and normalised using internal controls. Following background correction, manual inspection permitted removal of low quality

samples presenting issues relating to bisulphite conversion, staining signal, inadequate hybridisation or nucleotide extension. Quality control analyses were performed to remove probes with low detection rate <95% at $P < 0.01$. Samples with a low call rate (samples with <450000 probes detected at *p*-values of less than 0.01) were also eliminated. Furthermore, samples were removed if they had a poor match between genotype and SNP control probes, or incorrect DNA methylation-predicted sex.

Epigenome-wide association studies

Ordinary least squares regression model

In the ordinary least squares model strategy (*limma*; linear models for microarray data), each CpG site (n = 459309) was regressed on transformed protein levels with adjustments for age, sex, estimated white blood cell proportions ($CD4^+$ T cells, $CD8^+$ T cells, B cells, Natural Killer Cells and granulocytes) and technical covariates (plate, position, array, hybridisation, date) (4). Proportions of white blood cells were estimated from methylation data using the Houseman method (5).

Mixed linear model

In contrast to *limma*, CpG site (n = 459309) was the independent variable whereas Olink® protein levels were input as dependent variables in all mixed models (performed using **O**mic**S**-data-based **C**omplex trait **A**nalysis: OSCA) (6). The same covariates were adjusted for as in the limma strategy. The MOMENT method was used to test for associations between traits of interest and methylation at individual probes. MOMENT is a mixed linear model-based method that can account for unobserved confounders and the correlation between distal probes which may be introduced by such confounders. The same Bonferroni-corrected

threshold as the linear model was applied: 5.14×10^{-10} (= genome-wide significance: 3.6×10^{-8}/70 phenotypes).

Sherlock

A Bayesian algorithm termed Sherlock (7) was used to detect gene-protein associations by incorporating information from publicly available eQTL data and GWAS summary statistics. This was carried out to infer genes whose differential expression may contribute to alterations in circulating levels of Olink® inflammatory proteins. Sherlock identifies all *cis* and *trans* eQTLs or expression-associated SNPs (eSNPs) for a given gene in a selected data set. The algorithm evaluates the association of each eSNP with the trait of interest (i.e. protein levels) using supplied GWAS data. A score is assigned to each gene based on aligning P-values for the association of the SNP with gene expression and the studied trait. There are three possible scenarios which affect this gene-based score: (i) if the eSNP for the gene is also associated with the trait, a positive score is assigned, (ii) if the eSNP is not associated with the trait, a negative score is assigned and (iii) if the SNP is associated with the trait only (non-eSNP), the score is not affected. The total score of a gene increases in tandem with an increase in the number of SNPs with combined evidence (SNPs that are associated with trait and expression). For each SNP in the alignment, the logarithm of Bayes factor is computed and the sum of constituent SNPs in the gene constitutes the final score for the gene. SNPs that have moderate statistical significance in GWAS and eQTL data sets, that are otherwise missed by traditional GWAS thresholds, are considered. SNPs with stronger associations with the trait contribute more to the final gene-based score than moderately-associated variants. Default settings were applied. As our protein data was collected from whole blood, analyses were restricted

to the eQTL GTEx (V7) Whole Blood data set (n = 369) (8). Correction for multiple testing was carried out using the Benjamini-Hochberg procedure at a threshold of $P < 1.0 \times 10^{-5}$ (9).

Mendelian Randomisation

(i) Pruned protein QTL variants were used as instrumental variables (IV) to determine the relationship between circulating inflammatory protein biomarkers and their respective phenotypic associations, as identified through GWAS Catalog. Four proteins were shown to have an association with five human traits. Thus, a Bonferroni-corrected significance threshold of 0.01 (0.05/5 tests) was applied.

(ii) For 11/13 proteins, only one SNP remained after linkage disequilibrium (LD) pruning. For 2 proteins (CCL25 and CST5), two independent SNPs were present after pruning. Pruned SNPs were used as IV to test for causal associations between each of the 13 inflammatory proteins and risk of late-onset Alzheimer's disease (10). A Bonferroni-corrected significance threshold of 3.85×10^{-3} (0.05/13 tests) was applied. For one protein (IL18R1) which showed a nominally significant association with AD risk, a bidirectional analysis was performed to assess for a putatively causal association in which AD risk affected circulating IL18R1 levels. For this test, 22 independent SNPs remained after LD pruning.

(iii) Expression QTLs obtained from eQTLGen consortium were used as IV to test whether changes in gene expression were causally associated with protein levels (11).

(iv) One protein (IL18R1) harboured both genome- and epigenome-wide significant associations in this study. Therefore, we wished to determine whether methylation affected protein levels and/or whether protein levels affected

methylation. We used Phenoscanner to determine whether the pQTL identified for IL18R1 levels (rs917997) has been reported as a methylation QTL for the corresponding *cis* genome-wide significant CpG site identified for IL18R1 levels in our study (cg03938978) (12). The methylation QTL was used as an instrument to test whether altered DNA methylation was causally associated with inflammatory protein levels. Conversely, the IL18R1 pQTL was used as an instrument to assess whether altered IL18R1 levels were causally linked to differential methylation.

Conditional and joint analysis from ordinary least squares GWAS on protein levels

In an ordinary least squares (OLS) regression model, 1531 SNPs were associated with the levels of 19/70 proteins at a Bonferroni-corrected threshold ($P < 7.14 \times 10^{-10}$; Additional file 2: Table S3). Manhattan and Q-Q plots for these 19 proteins are presented in Appendices 1 and 2, respectively. Estimates for inflation factors across each of the 70 genome-wide association studies are listed in Additional file 2: Table S4. Conditional and joint analysis (GCTA-COJO) was performed to identify which of these hits were independent of one another, resulting in the identification of 27 conditionally significant pQTLs associated with the circulating levels of 17 proteins (Additional file 2: Table S5). Of note, whereas Bonferroni-corrected genome-wide significant SNPs were identified for an additional two proteins (CCL23 and MMP-10), the conditional P value for these pQTLs from GCTA-COJO did not fall below the Bonferroni-corrected threshold of $P < 7.14 \times 10^{-10}$.

Sherlock: identifying genes whose expression associates with inflammatory biomarkers

The Bayesian algorithm termed Sherlock uses *cis* and *trans* eQTLs to assign gene-based scores from GWAS data to identify genes whose expression associates with a trait of interest (here,

protein levels). Putative gene expression-protein associations for all 13 proteins are outlined in Additional file 2: Table S11. From this gene-based colocalisation approach, only gene expression of *ADA*, *CXCL5* and *IL18R1* were associated with levels of their respective protein products. Expression of *MIF4GD* and *GRB2* were both associated with CD6 and TNFB levels (*r* between levels: 0.45). Expression of *TNRC6A* was associated with CD6 and ADA levels (*r:* 0.55). *CXCL4L1* expression was associated with CXCL5 and CXCL6 levels (*r*: 0.44).

The disparity between the employed colocalisation methods, *coloc* and Sherlock, may reflect differences in technical and biological variability. The former method considers *cis* regions in a continuous chromosomal region as defined by the pQTL whereas the latter takes into account all *cis* and *trans* eQTLs across the genome passing a soft significance threshold. Both transcript datasets and our pQTL estimates were generated in different samples which may have resulted in different molecular abundances and differences in the overlapping of transcript and protein distributions. The small sample sizes used to generate the datasets may have limited power to detect further *cis* gene expression-protein patterns.

GWAS and EWAS of CCL11 levels – incorporating smoking status as a covariate

The smoking-associated *AHRR* probe, cg05575921, was associated with CCL11 levels in the OLS regression-, mixed model- and Bayesian penalised regression-based EWAS. Adjustment for smoking attenuated this association in the OLS regression model, as detailed in the main text. Therefore, we repeated the EWAS of CCL11 levels adjusting for smoking status as an additional covariate. We also repeated the GWAS of CCL11 levels, through OLS regression and BayesR+, adjusting for smoking status.

Beginning with the genome-wide association studies, one SNP was significantly associated with CCL11 levels and survived multiple testing correction in the OLS regression GWAS following adjustment for smoking (rs2228467; effect allele: C, beta: 0.60, se: 0.10, P: 5.14×10^{-10}). This SNP was annotated to the *ACKR2* gene. The association between rs2228467 and CCL11 levels has previously been reported in two studies (13, 14). Beta coefficients were correlated 98% between the models with and without smoking status as a covariate. In the BayesR+ GWAS, no markers were significantly associated with CCL11 levels. However, the SNP with the highest posterior inclusion probability was rs2228467 at 48%. The variance in CCL11 levels explained by genetic data was 25.5% prior to adjustment for smoking and was 23.6% following controlling for smoking levels.

In the OLS regression-based EWAS, t-statistics were correlated 96% between the models with and without smoking status as a covariate. After accounting for smoking status, the association between the smoking-associated *AHRR* probe, cg05575921, and CCL11 levels was attenuated by 31.03% to non-significance. Twenty-one probes showed a difference in t-statistic > 3 between the model without smoking as a covariate and the model with smoking status included as a covariate. These probes were all annotated to either *AHRR*, *F2RL3*, *GFI1* or *RARA*. Differential methylation levels in these genes have been strongly linked to smoking status (15-18). In the mixed model-based EWAS, the beta coefficients were correlated 97% between the model without smoking as a covariate and the model which incorporated smoking status. The association between CCL11 levels and the cg05575921 probe was attenuated to non-significance upon controlling for smoking status (before adjustment: beta:

-1.96, P: 4.86 x 10^{-10}, after adjustment: beta: -0.21, P: 0.84; % attenuation: 89.29%). In the BayesR+ EWAS, no probes were significantly associated with CCL11 levels. As with the OLS regression and mixed model strategies, the association between CCL11 levels and the cg05575921 probe was attenuated to non-significance. The variance in CCL11 levels explained by methylation data was 26.3% prior to adjustment for smoking and was 19.6% upon controlling for smoking status. This reflects a decrease of 6.7%.

Replication of previous pQTLs and protein associated-CpG sites

Phenoscanner was used to search for previously reported pQTL associations (12). Default settings for the software were used, the "pQTL" catalogue was searched and the r^2 value between input SNPs and proxy SNPs was set to 0.8. Summary statistics from four major pQTL studies were extracted to determine whether the 13 pQTLs identified in this study replicated those of previous findings (19-22). Following an additional literature search, we also cross-referenced findings from Höglund *et al.*, Di Narzo *et al.,* Enroth *et al.*, Sun *et al.* for look-up analysis (13, 23-25). Across studies, all 13 proteins were available for look-up. Eleven (84.6%) of our conditionally significant and robustly identified pQTLs replicated previously reported genome-wide significant signals. Beta coefficients were correlated 88% between our study and those reported in previous studies. Of these eleven pQTLs, five were reported in just one other study (Table 1). Three pQTLs (rs2032887 for CCL25, rs6851997 for CXCL6 and rs10045431 for IL12B) were reported in two studies. Three pQTLs (rs425535 for CXCL5, rs3138036 for MCP2, rs917997 for IL18R1) were previously reported in four studies. This totalled 23 comparisons. We report two pQTL associations which have not yet been reported as genome-wide significant in the literature. These were identified by both OLS regression

and BayesR+ GWAS strategies. These are rs11700291 for ADA levels and rs1458038 for FGF-5 levels.

Additionally, we extracted beta coefficients for all pQTLs which have been previously reported as genome-wide significant in the literature. We sought to determine how well these beta coefficients correlated with beta values from our GWAS. This was carried out for all 70 proteins in our study, using Phenoscanner and manual literature searches. Of note, many of these pQTLs were non-significant in our study. Forty-two of the proteins had known pQTLs and these were identified across 8 studies (n = 1567 beta comparisons in total). We observed a strong correlation between effect sizes for previously reported pQTLs and corresponding effect sizes in our study (r: 0.70, 95% CI: [0.67, 0.72], Fig. 2).

Of the 3 proteins with significant CpG sites (n = 3) identified by multiple methods, 1 was available for look-up from the EWAS on inflammatory proteins performed by Ahsan *et al*. (26). This CpG-protein association was replicated in our study (cg07839457 (*NLRC5*) for CXCL9 levels; $beta_{LBC}$: -2.91 vs. $beta_{Ahsan}$: -3.26).

BayesR+ combined analysis – GWAS and EWAS modelled together

In the combined GWAS and EWAS, 2/3 CpG sites which were concordantly identified across all methods were present after adjusting for underlying genetic architecture (Additional file 2: Table S18). The *cis* association between cg03938978 and IL18R1 levels was not observed after accounting for SNP data, in keeping with the role of rs917997 as a *cis*-acting mQTL. In total, 6 CpG sites were present in the combined BayesR+ model, showing an overlap of 6/8

CpG sites with the stand-alone BayesR+ EWAS (Additional file 2: Table S11). In addition to the cg03938978 and IL18R1 association, the association between cg05575921 and TGF-alpha was also attenuated after accounting for genetic factors.

Eighteen pQTLs were identified in the combined BayesR+ analysis which accounted for genetic and epigenetic data together. Of these, 12 were also present in the stand-alone BayesR+ GWAS alone (Additional file 2: Table S2), with a further 3 sites directly replicating independent pQTLs identified by OLS regression models (Additional file 2: Table S5). Of the remaining 3 sites, one was in high LD with a corresponding variant identified in the stand-alone BayesR+ GWAS (CCL25: *trans* variants at *FUT2*, rs602662 and rs485186, respectively). One pQTL (rs9899183) for TWEAK was in low LD with the pQTL for TWEAK levels identified by OLS regression. The remaining pQTL identified in the combined analysis (rs17386472) was associated with GDNF levels. GDNF did not have a significant genetic signal in the stand-alone BayesR+ GWAS and although OLS regression identified pQTLs for GDNF, none of these survived multiple testing correction following conditional and joint analyses.

Evaluating causal associations between blood inflammatory proteins and Alzheimer's risk

Using two-sample Mendelian randomisation, we tested whether the 13 inflammatory proteins with significant genetic correlates in our study were causally associated with Alzheimer's disease risk (Additional file 2: Table S19). One protein, IL18R1, showed a nominally significant association with AD risk (no. of instruments: 1, beta: 0.02, se: 0.01, P: 0.04; Wald ratio test). Conversely, AD risk was not associated with IL18R1 levels (no. of instruments: 22, beta: 0.03, se: 0.21, P: 0.85; inverse variance-weighted method). The

intercept from MR Egger regression was −0.07 (P = 0.11) which does not provide evidence for directional pleiotropy.

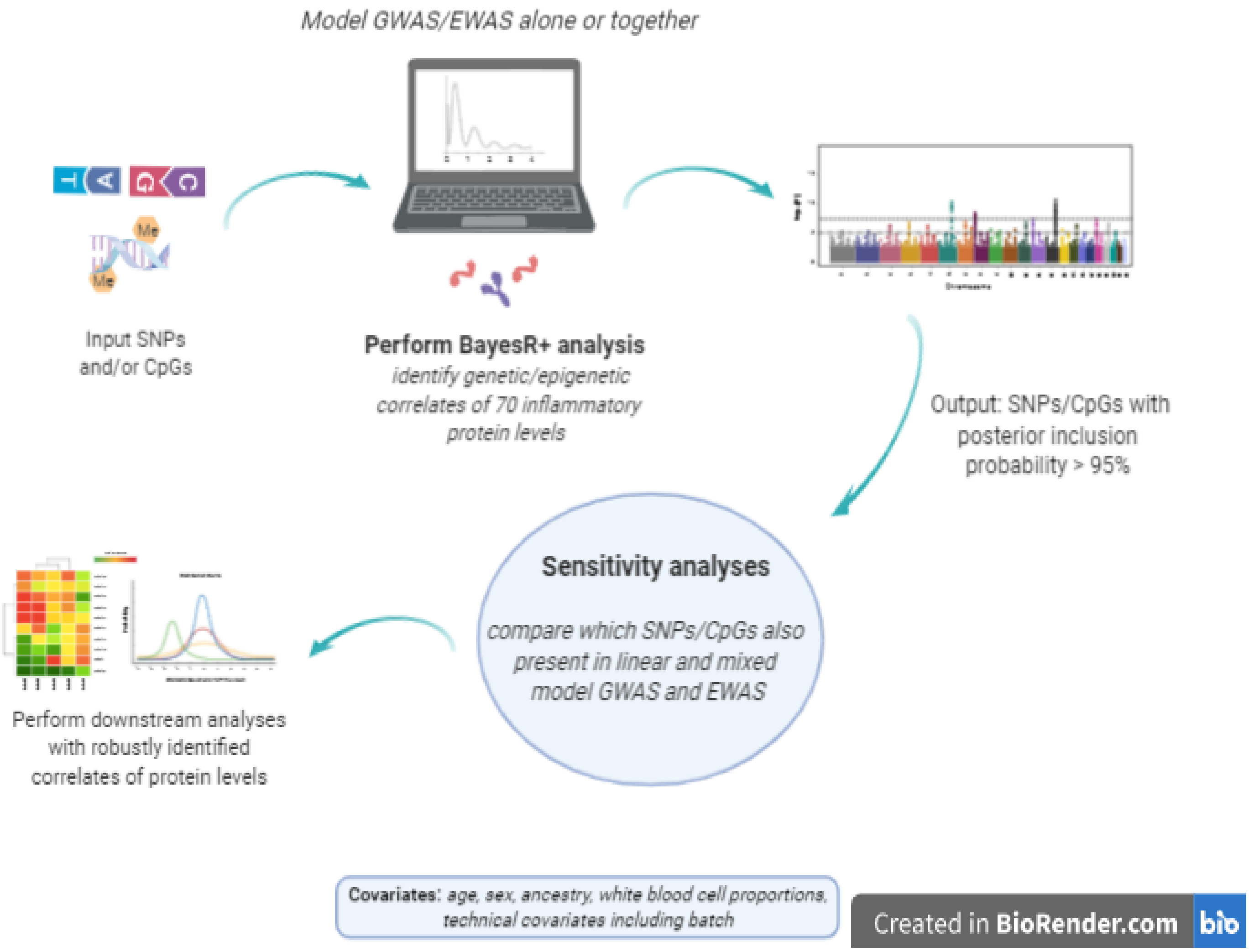

Fig. 1. Computational strategy of present study. Seventy inflammatory protein biomarkers present on the Olink® inflammation panel surpassed quality control procedures. We performed genome-wide and epigenome-wide association studies on the levels of these proteins in 876 healthy older adults who are participants of the Lothian Birth Cohort 1936 study. The primarily analysis was Bayesian penalised regression, using BayesR+, which allowed for the modelling of relationships between SNPs or CpGs and protein levels whilst accounting for known and unknown confounding variables and holding all other molecular markers as covariates. This allowed for estimates of single probe or marker coefficients conditional on all other probes or markers in the

input dataset. Furthermore, this strategy provided joint estimates for the contribution of genetic and epigenetic factors towards inter-individual variability in inflammatory protein levels, modelled both alone and together. We performed sensitivity analyses to determine whether SNPs and CpGs which were significantly associated with protein levels in the BayesR+ methodology (posterior inclusion probability > 95%), were also significantly associated with protein levels in ordinary least squares (OLS) regression and mixed model-based methods. Only those robustly identified SNPs and CpGs which were present in all methods (OLS and BayesR+ for GWAS, and OLS, mixed model (OSCA) and BayesR+ for EWAS) were considered for downstream analyses, including colocalisation, Mendelian randomisation and pathway enrichment analyses. Importantly, this also showed how well the different methods overlapped in relation to their identification of molecular correlates of inflammatory protein levels, and their estimates for phenotypic variance in protein levels explained by genetic and epigenetic factors. This is an important result of the present study. Protein data were pre-corrected for age, sex, array and ancestry. Additionally, where possible, the same covariates were controlled for across all methods, such as Houseman-estimated white blood cell proportions for EWAS analyses. Multiple testing correction was carried out by the Bonferroni method, accounting for all SNPs or CpGs used in the respective analyses. Image created using Biorender.com.

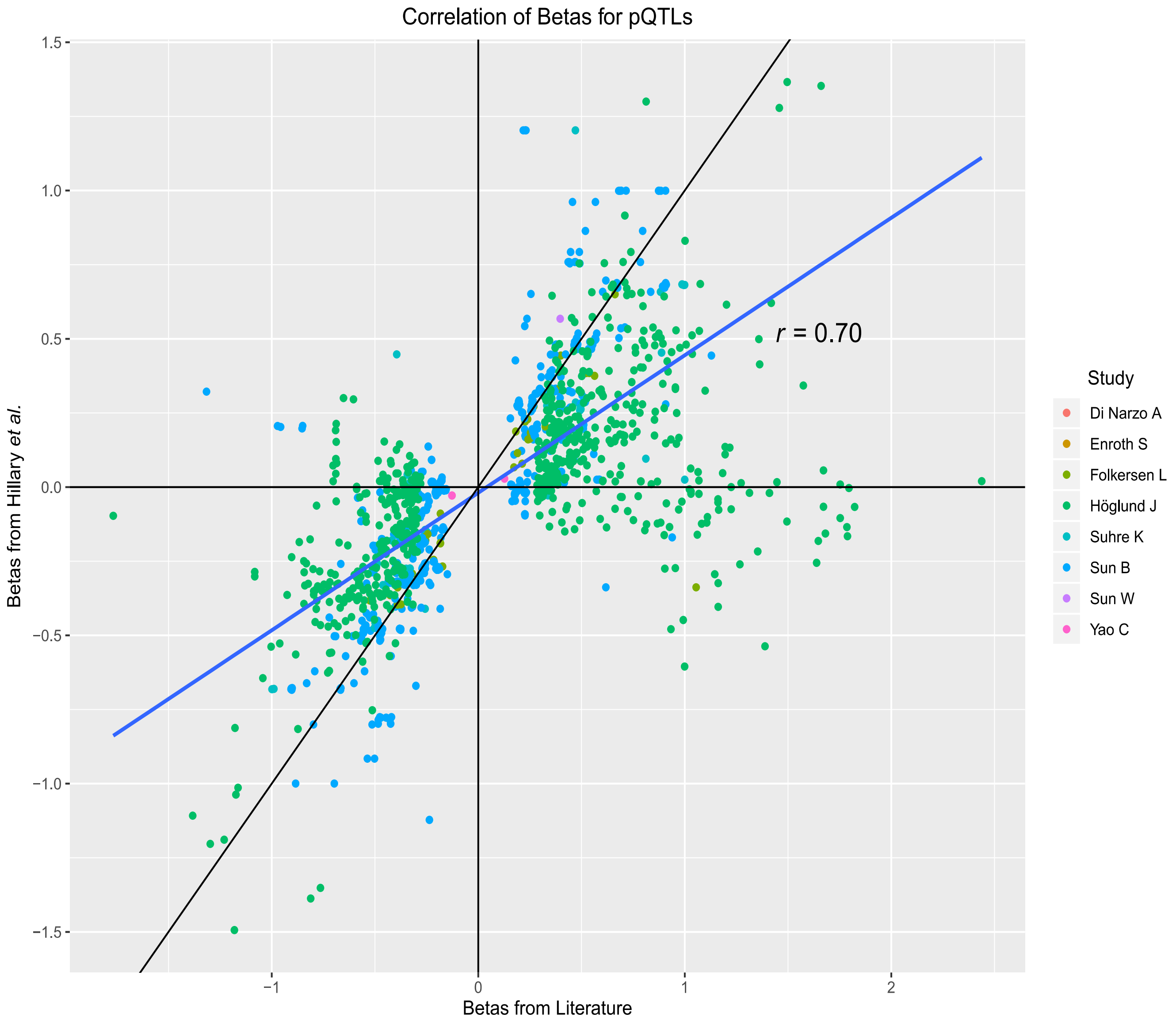

Fig. 2. Correlation of beta values for pQTL associations derived from literature with beta values from the present study. SNPs which were in high linkage disequilibrium ($r^2 \geq 0.8$) with input SNPs on Phenoscanner or from manual extraction of relevant studies were included in the analyses.

Table 1. Overlap between conditionally significant pQTLs identified by both OLS regression and BayesR+ and previously reported genome-wide significant pQTLs in literature.

**Protein**	**SNP**	**Beta from Literature**	**Study**	**Beta from Hillary *et al.***
CCL25	rs2032887	-0.30	Sun B	-0.67
CCL25	rs2032887	-0.72	Höglund J	-0.70
CD6	rs2032887	0.78	Höglund J	0.41
CST5	rs11230563	0.65	Höglund J	0.68
CXCL5	rs425535	0.24	Sun B	0.57
CXCL5	rs425535	0.95	Höglund J	0.57
CXCL5	rs425535	0.40	Sun W	0.57
CXCL5	rs425535	NA	Enroth S	0.57
CXCL6	rs6851997	-0.61	Folkersen L	-0.37
CXCL6	rs6851997	-0.67	Höglund J	-0.61
IL10RB	rs2247526	0.63	Höglund J	0.36
IL12B	rs10045431	-0.66	Höglund J	-0.45
IL12B	rs10045431	-0.26	Sun W	-0.45
IL18R1	rs917997	0.96	Suhre K	0.68
IL18R1	rs917997	0.65	Sun B	0.68
IL18R1	rs917997	0.89	Sun B	0.68
IL18R1	rs917997	1.07	Höglund J	0.68
MCP2	rs3138036	0.47	Suhre K	1.20
MCP2	rs3138036	0.22	Sun B	1.20

MCP2	rs3138036	1.23	Höglund J	1.20
MCP2	rs3138036	NA	Di Narzo A	1.20
MCP4	rs12075	-0.72	Höglund J	-0.62
TNFB	rs2229092	-1.76	Höglund J	-1.15

References

1. Li Y, Willer C, Sanna S, Abecasis G. Genotype imputation. Annual review of genomics and human genetics. 2009;10:387-406.
2. Li Y, Willer CJ, Ding J, Scheet P, Abecasis GR. MaCH: using sequence and genotype data to estimate haplotypes and unobserved genotypes. Genet Epidemiol. 2010;34(8):816-34.
3. Yang J, Ferreira T, Morris AP, Medland SE, Madden PA, Heath AC, et al. Conditional and joint multiple-SNP analysis of GWAS summary statistics identifies additional variants influencing complex traits. Nature genetics. 2012;44(4):369-75, s1-3.
4. Ritchie ME, Phipson B, Wu D, Hu Y, Law CW, Shi W, et al. limma powers differential expression analyses for RNA-sequencing and microarray studies. Nucleic acids research. 2015;43(7):e47-e.
5. Houseman EA, Accomando WP, Koestler DC, Christensen BC, Marsit CJ, Nelson HH, et al. DNA methylation arrays as surrogate measures of cell mixture distribution. BMC bioinformatics. 2012;13(1):86.
6. Zhang F, Chen W, Zhu Z, Zhang Q, Nabais MF, Qi T, et al. OSCA: a tool for omic-data-based complex trait analysis. Genome biology. 2019;20(1):107.
7. He X, Fuller CK, Song Y, Meng Q, Zhang B, Yang X, et al. Sherlock: detecting gene-disease associations by matching patterns of expression QTL and GWAS. American journal of human genetics. 2013;92(5):667-80.
8. Aguet F, Brown AA, Castel SE, Davis JR, He Y, Jo B, et al. Genetic effects on gene expression across human tissues. Nature. 2017;550(7675):204-13.
9. Benjamini Y, Hochberg Y. Controlling the False Discovery Rate: A Practical and Powerful Approach to Multiple Testing. Journal of the Royal Statistical Society Series B (Methodological). 1995;57(1):289-300.
10. Marioni RE, Harris SE, Zhang Q, McRae AF, Hagenaars SP, Hill WD, et al. GWAS on family history of Alzheimer's disease. Translational psychiatry. 2018;8(1):99.
11. Võsa U, Claringbould A, Westra H-J, Bonder MJ, Deelen P, Zeng B, et al. Unraveling the polygenic architecture of complex traits using blood eQTL meta-analysis. bioRxiv. 2018:447367.
12. Staley JR, Blackshaw J, Kamat MA, Ellis S, Surendran P, Sun BB, et al. PhenoScanner: a database of human genotype-phenotype associations. Bioinformatics (Oxford, England). 2016;32(20):3207-9.
13. Höglund J, Rafati N, Rask-Andersen M, Enroth S, Karlsson T, Ek WE, et al. Improved power and precision with whole genome sequencing data in genome-wide association studies of inflammatory biomarkers. Scientific reports. 2019;9(1):16844-.
14. Ahola-Olli AV, Wurtz P, Havulinna AS, Aalto K, Pitkanen N, Lehtimaki T, et al. Genome-wide Association Study Identifies 27 Loci Influencing Concentrations of Circulating Cytokines and Growth Factors. Am J Hum Genet. 2017;100(1):40-50.

15. Lee DH, Hwang SH, Lim MK, Oh JK, Song DY, Yun EH, et al. Performance of urine cotinine and hypomethylation of AHRR and F2RL3 as biomarkers for smoking exposure in a population-based cohort. PloS one. 2017;12(4):e0176783.
16. Joehanes R, Just AC, Marioni RE, Pilling LC, Reynolds LM, Mandaviya PR, et al. Epigenetic Signatures of Cigarette Smoking. Circ Cardiovasc Genet. 2016;9(5):436-47.
17. Ambatipudi S, Cuenin C, Hernandez-Vargas H, Ghantous A, Le Calvez-Kelm F, Kaaks R, et al. Tobacco smoking-associated genome-wide DNA methylation changes in the EPIC study. Epigenomics. 2016;8(5):599-618.
18. Tsaprouni LG, Yang T-P, Bell J, Dick KJ, Kanoni S, Nisbet J, et al. Cigarette smoking reduces DNA methylation levels at multiple genomic loci but the effect is partially reversible upon cessation. Epigenetics. 2014;9(10):1382-96.
19. Sun BB, Maranville JC, Peters JE, Stacey D, Staley JR, Blackshaw J, et al. Genomic atlas of the human plasma proteome. Nature. 2018;558(7708):73-9.
20. Suhre K, Arnold M, Bhagwat AM, Cotton RJ, Engelke R, Raffler J, et al. Connecting genetic risk to disease end points through the human blood plasma proteome. Nature communications. 2017;8:14357.
21. Lourdusamy A, Newhouse S, Lunnon K, Proitsi P, Powell J, Hodges A, et al. Identification of cis-regulatory variation influencing protein abundance levels in human plasma. Human molecular genetics. 2012;21(16):3719-26.
22. Emilsson V, Ilkov M, Lamb JR, Finkel N, Gudmundsson EF, Pitts R, et al. Co-regulatory networks of human serum proteins link genetics to disease. Science (New York, NY). 2018;361(6404):769-73.
23. Di Narzo AF, Telesco SE, Brodmerkel C, Argmann C, Peters LA, Li K, et al. High-Throughput Characterization of Blood Serum Proteomics of IBD Patients with Respect to Aging and Genetic Factors. PLoS genetics. 2017;13(1):e1006565.
24. Sun W, Kechris K, Jacobson S, Drummond MB, Hawkins GA, Yang J, et al. Common Genetic Polymorphisms Influence Blood Biomarker Measurements in COPD. PLoS genetics. 2016;12(8):e1006011-e.
25. Enroth S, Maturi V, Berggrund M, Enroth SB, Moustakas A, Johansson Å, et al. Systemic and specific effects of antihypertensive and lipid-lowering medication on plasma protein biomarkers for cardiovascular diseases. Scientific reports. 2018;8(1):5531.
26. Ahsan M, Ek WE, Rask-Andersen M, Karlsson T, Lind-Thomsen A, Enroth S, et al. The relative contribution of DNA methylation and genetic variants on protein biomarkers for human diseases. PLoS genetics. 2017;13(9):e1007005.

Appendix 1

Manhattan Plots – Hillary *et al.*

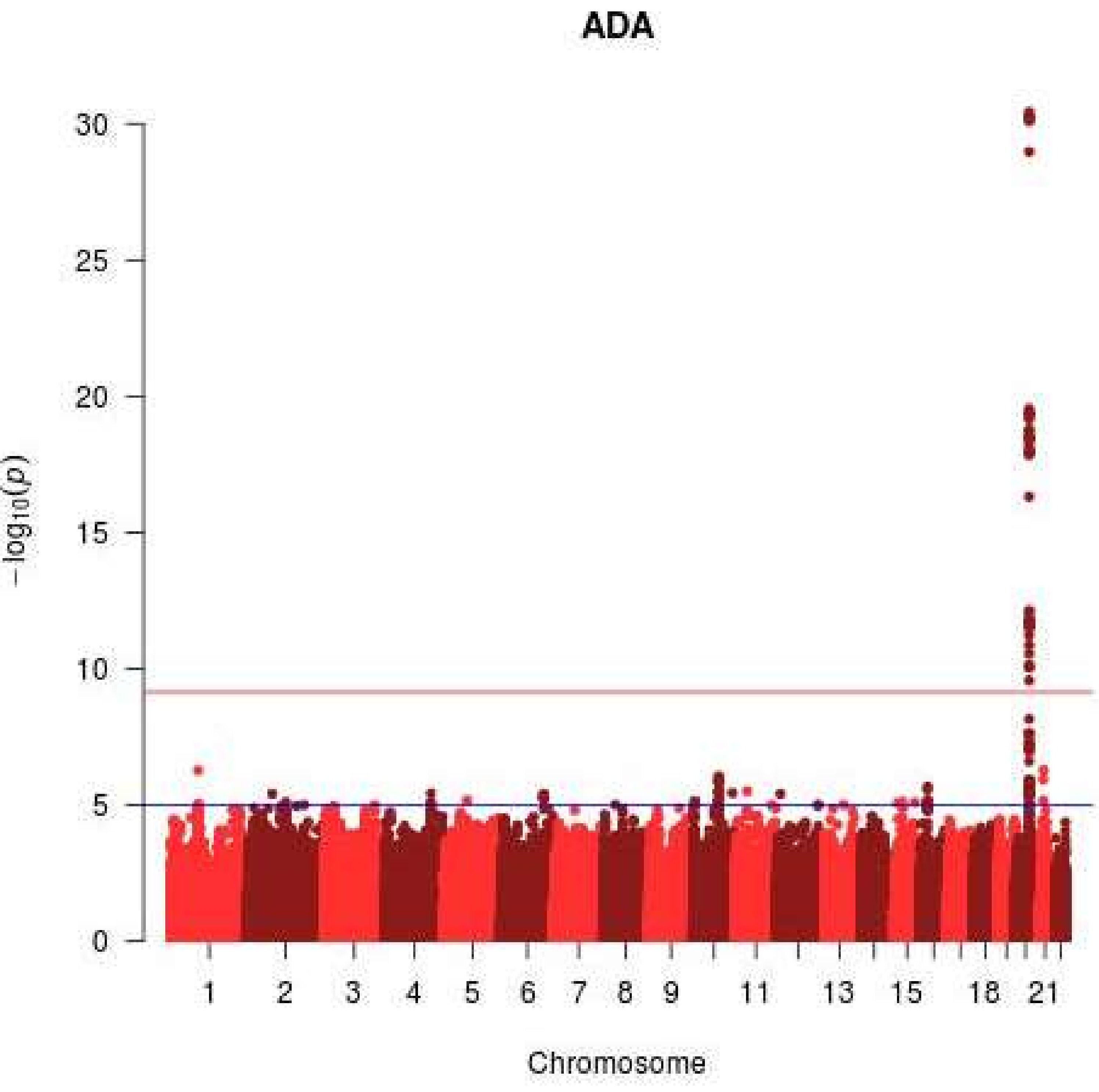
ADA
30
25
20
15
10
5
0
−log10(p)
1
2
3
4
5
6
7
8
9
11
13
15
18
21
Chromosome

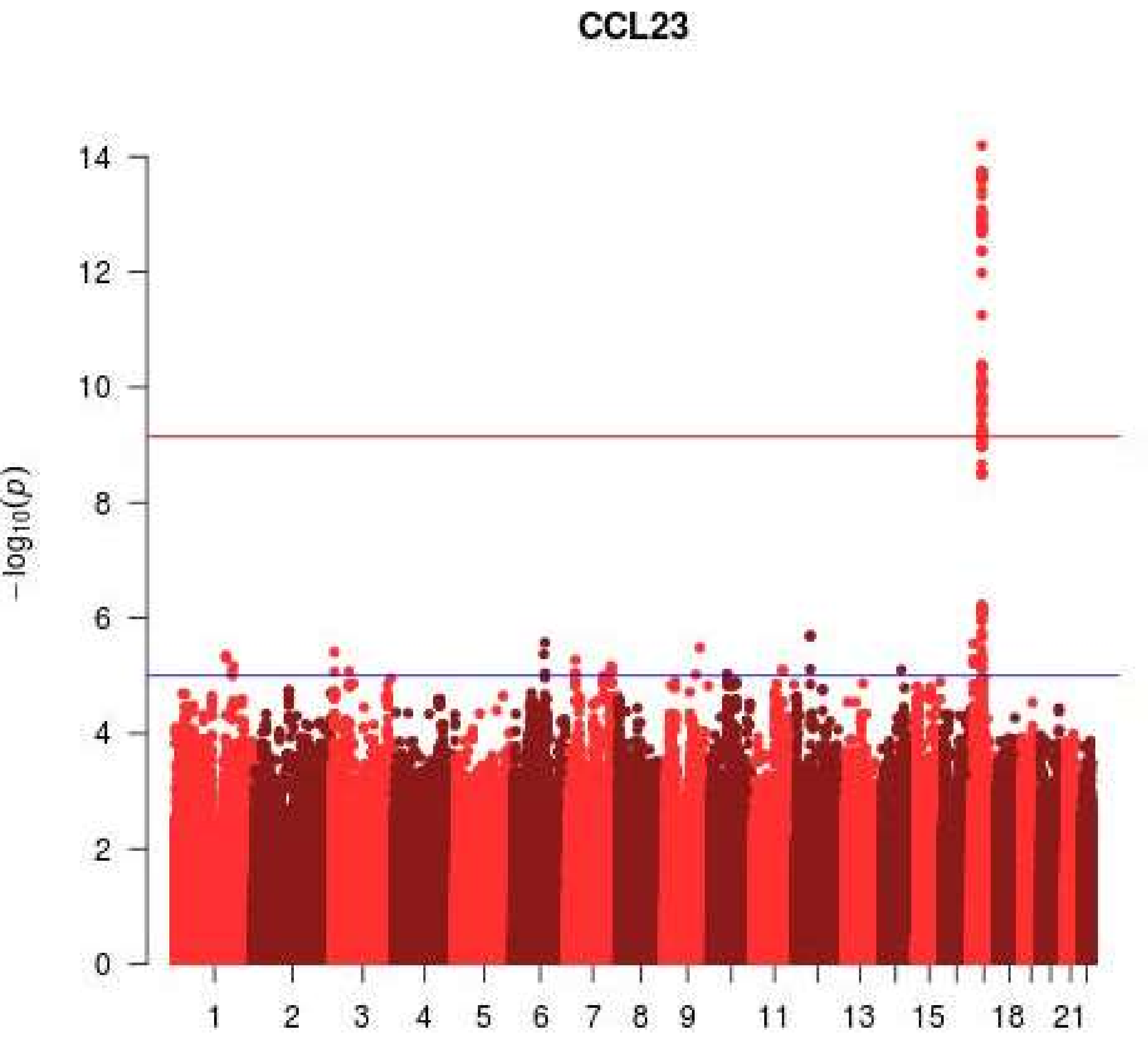
CCL23
14
12
10
8
6
4
2
0
$-\log_{10}(p)$
1
2
3
4
5
6
7
8
9
11
13
15
18
21
Chromosome

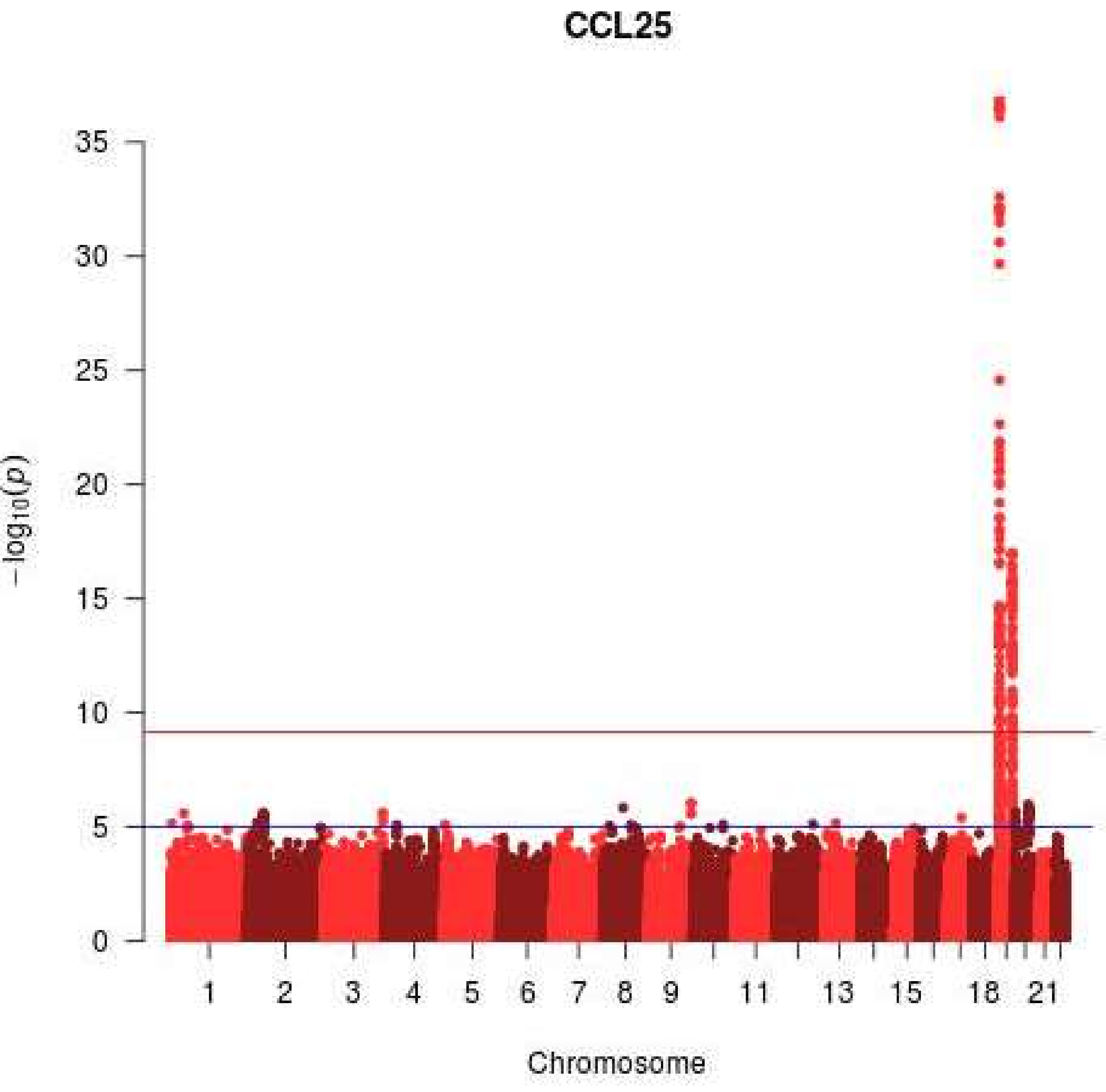
CCL25
35
30
25
20
15
10
5
0
$-\log_{10}(p)$
1
2
3
4
5
6
7
8
9
11
13
15
18
21
Chromosome

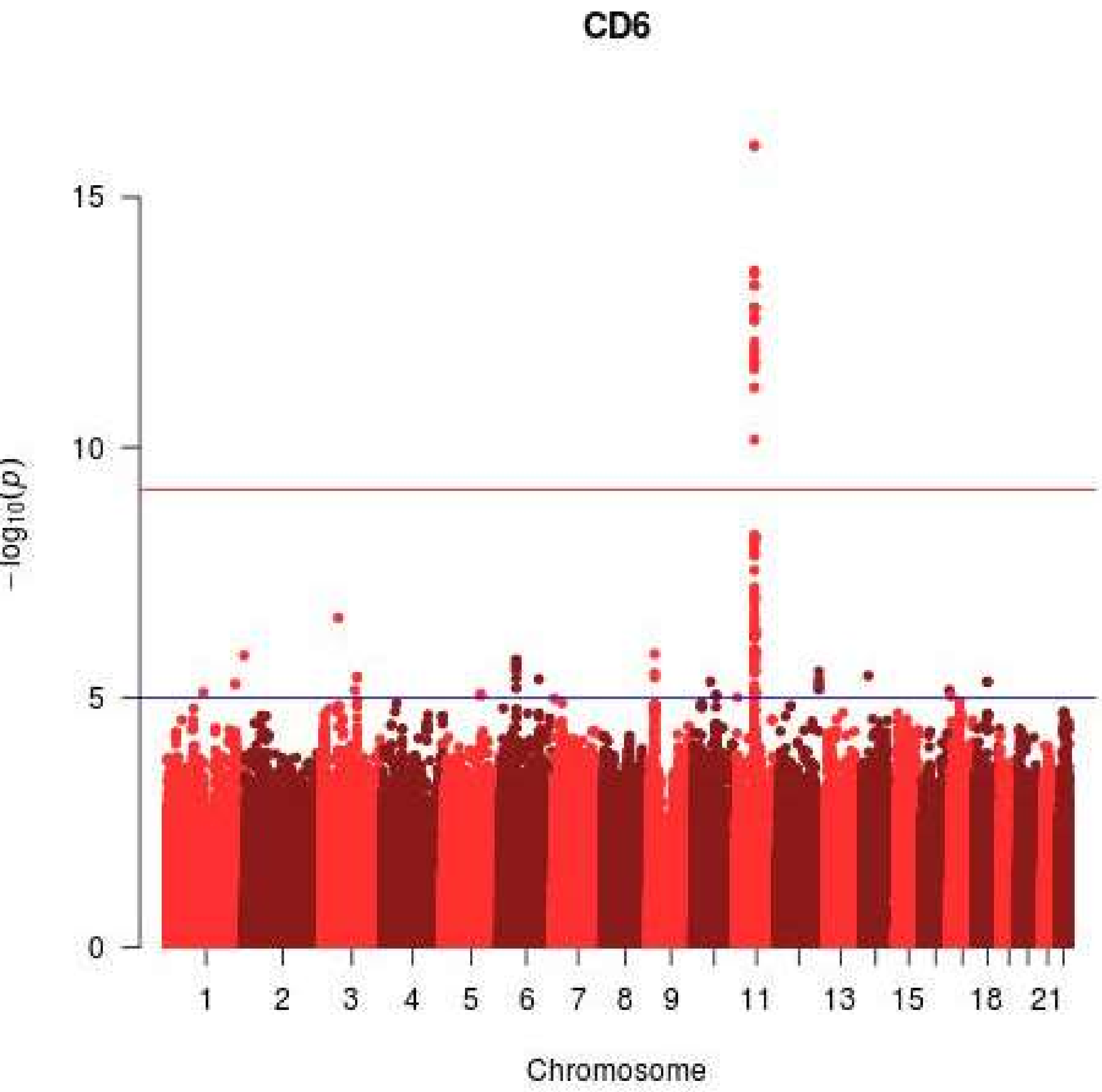

CD6
15
10
5
0
−log10(p)
1
2
3
4
5
6
7
8
9
11
13
15
18
21
Chromosome

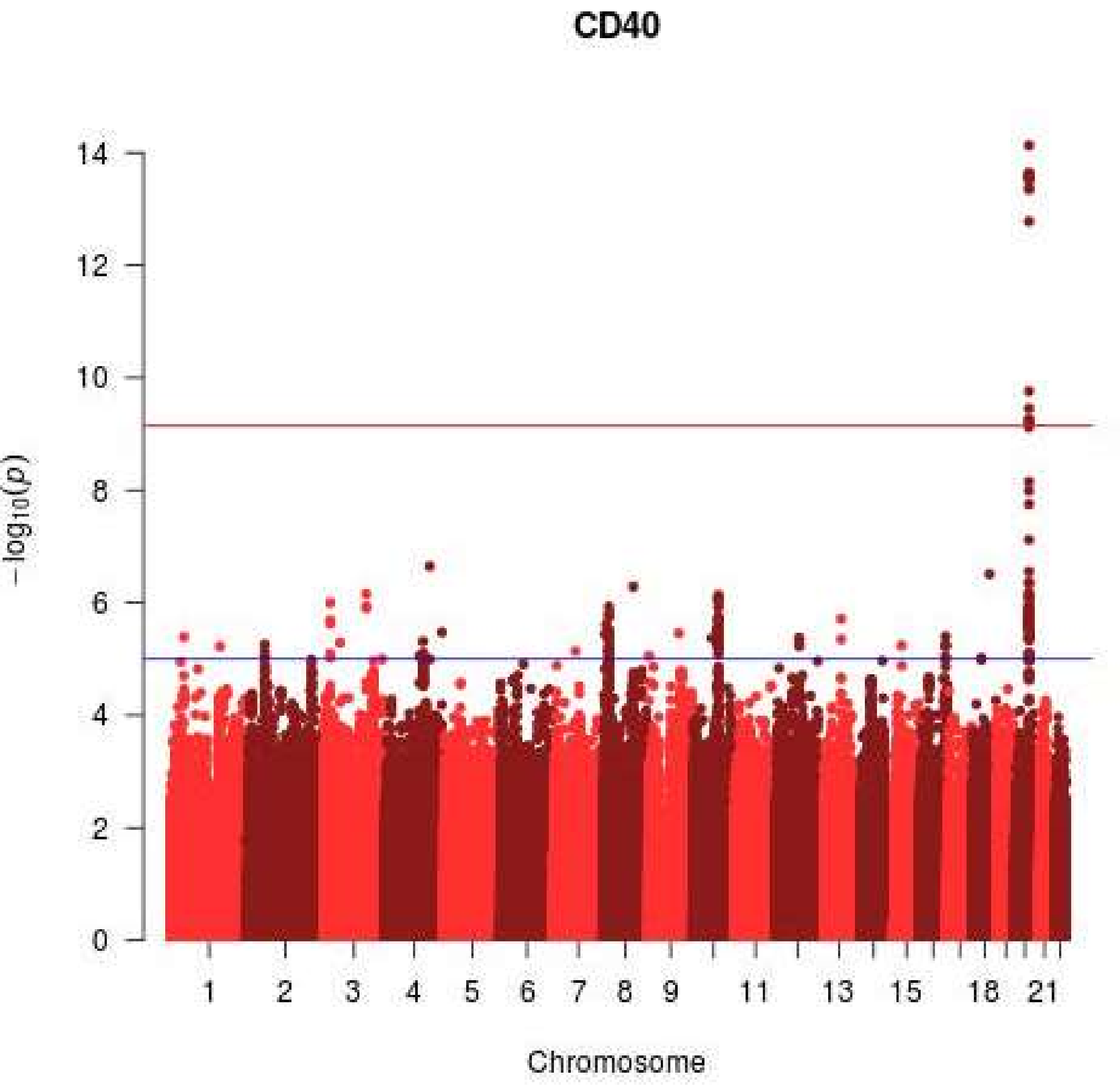

CD40
14
12
10
8
6
4
2
0
$-\log_{10}(p)$
1
2
3
4
5
6
7
8
9
11
13
15
18
21
Chromosome

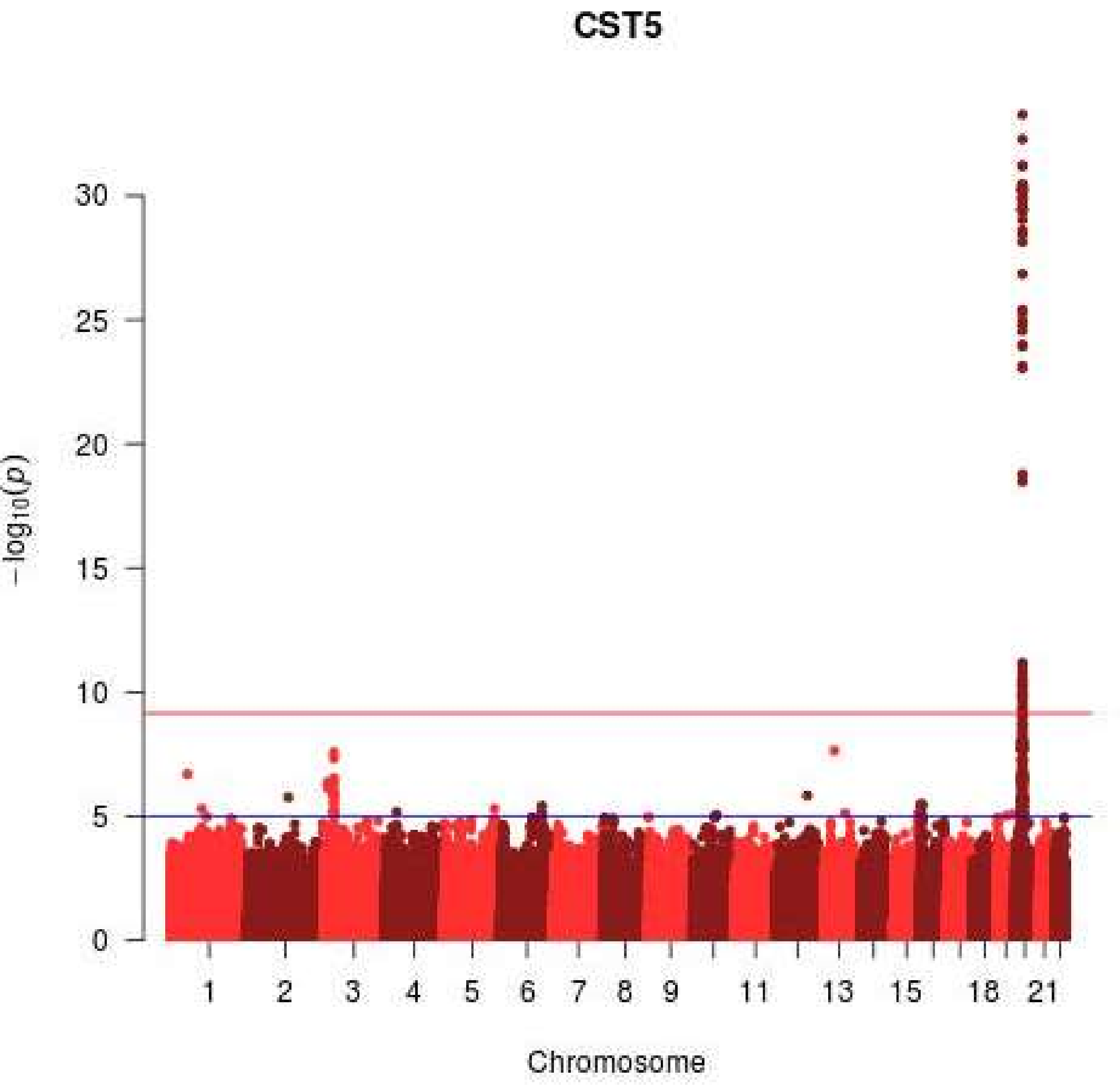

CST5
30
25
20
15
10
5
0
$-\log_{10}(p)$
1
2
3
4
5
6
7
8
9
11
13
15
18
21
Chromosome

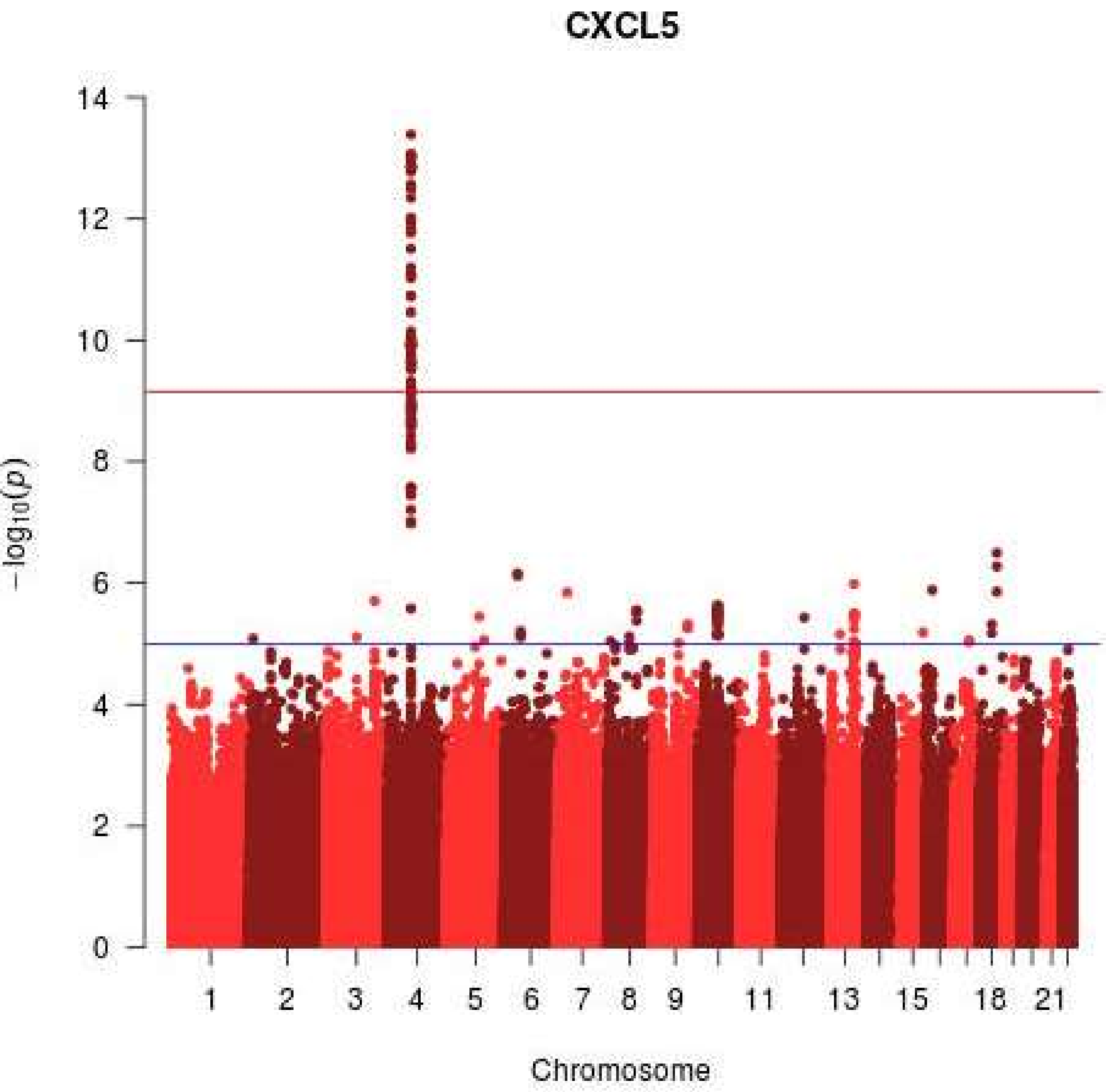
CXCL5
14
12
10
8
6
4
2
0
$-\log_{10}(p)$
1
2
3
4
5
6
7
8
9
11
13
15
18
21
Chromosome

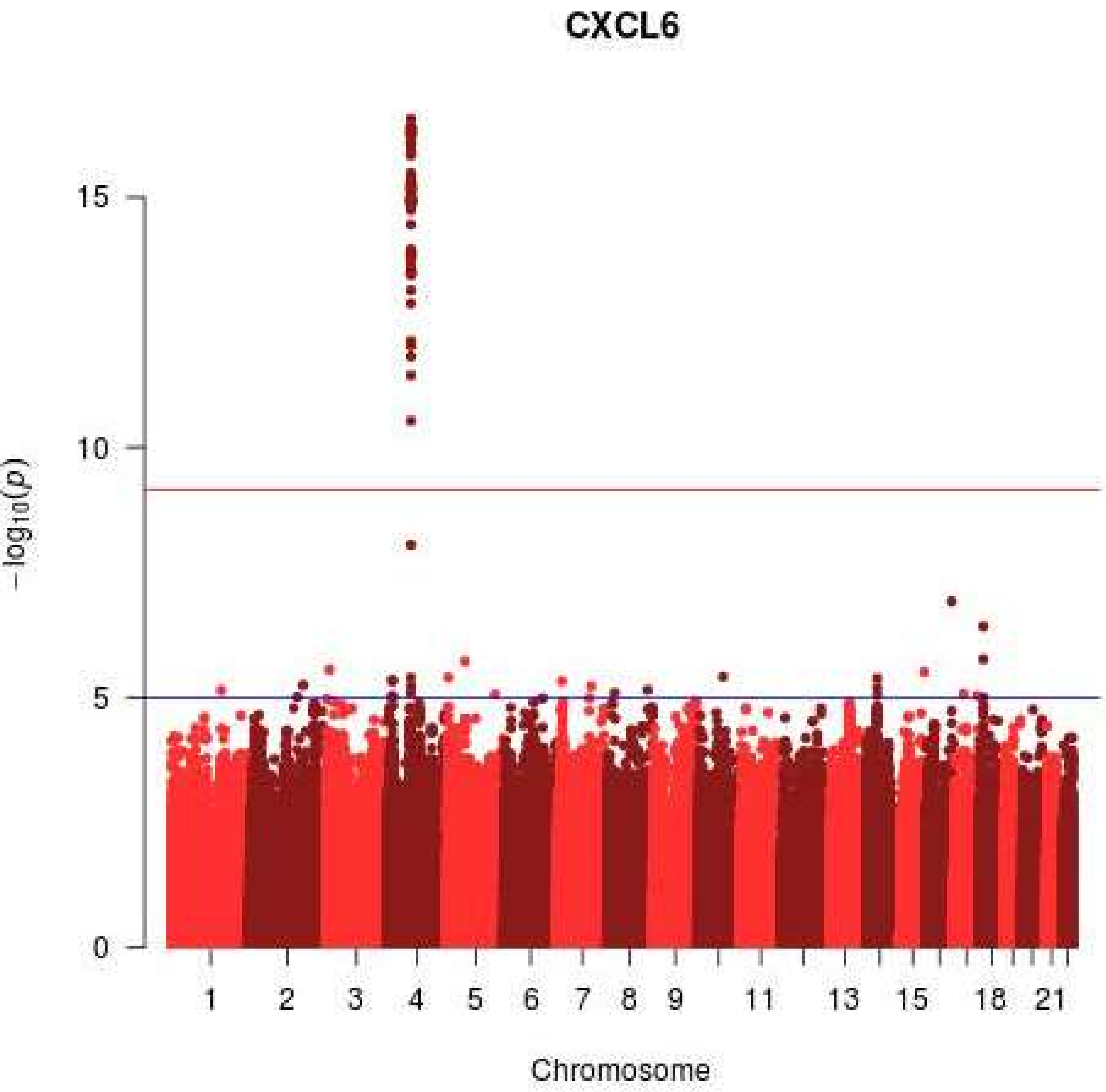
CXCL6
15
10
5
0
$-\log_{10}(p)$
1
2
3
4
5
6
7
8
9
11
13
15
18
21
Chromosome

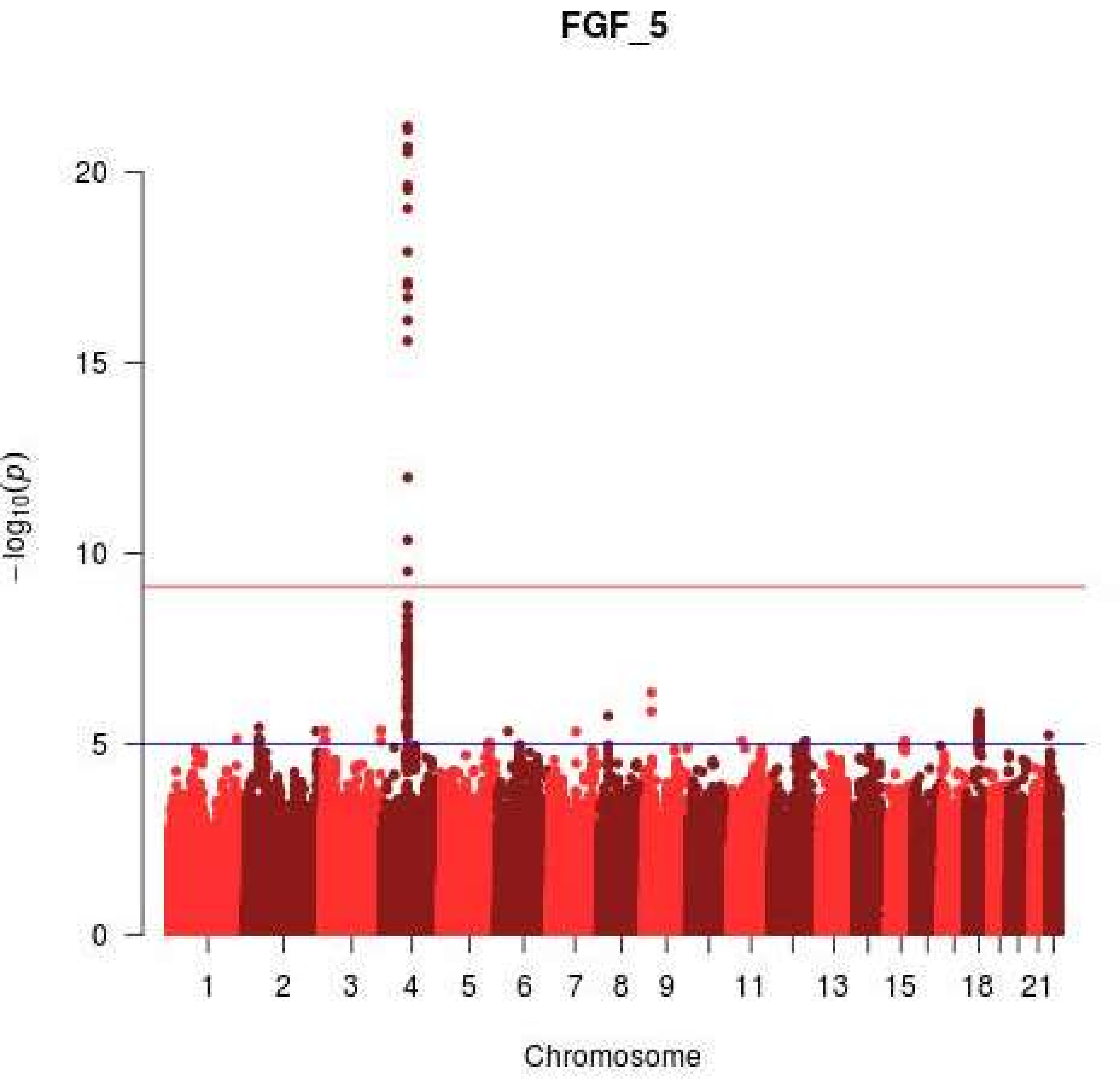
FGF_5
20
15
10
5
0
−log10(p)
1
2
3
4
5
6
7
8
9
11
13
15
18
21
Chromosome

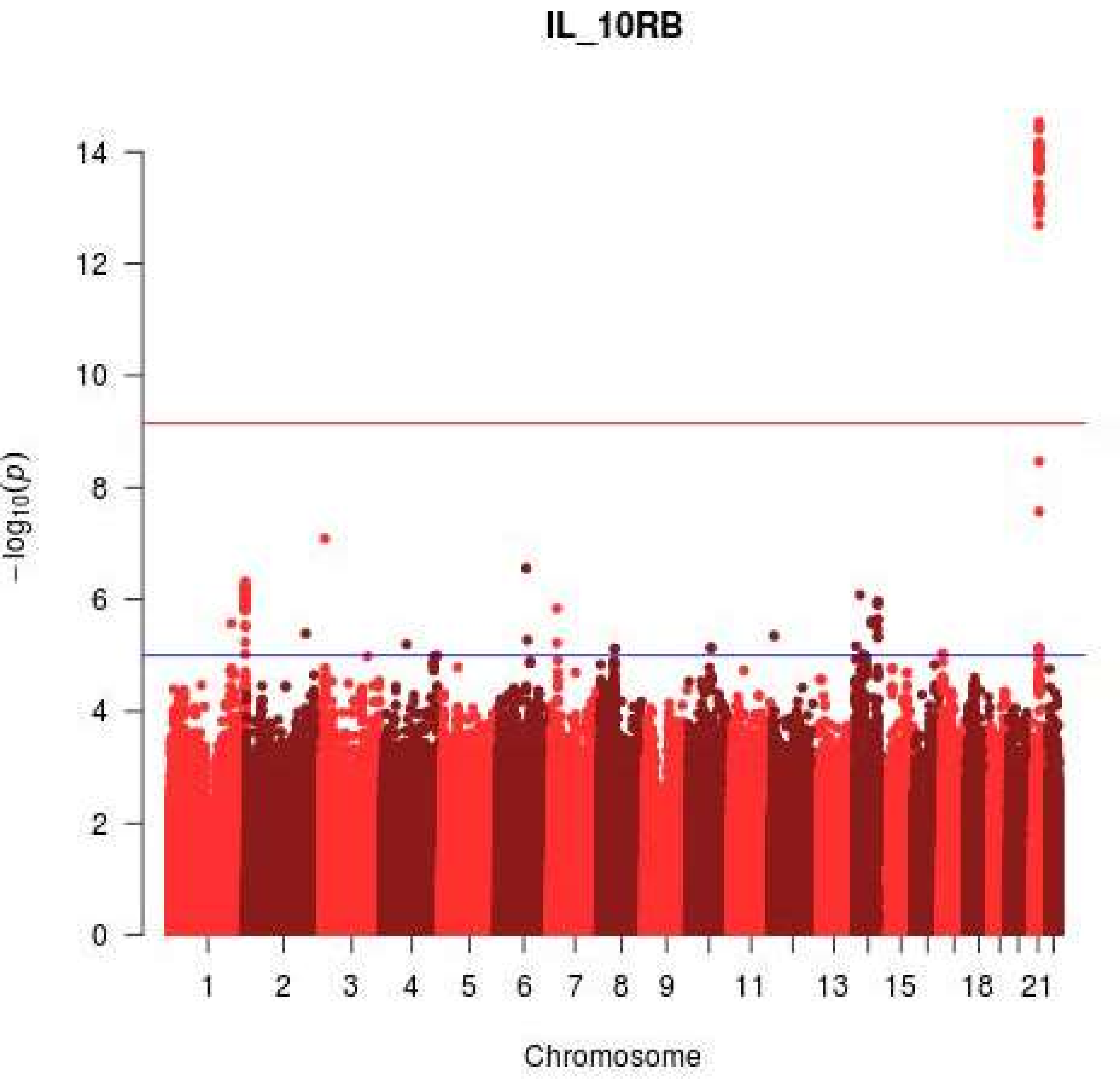
IL_10RB
14
12
10
8
6
4
2
0
−log10(p)
1
2
3
4
5
6
7
8
9
11
13
15
18
21
Chromosome

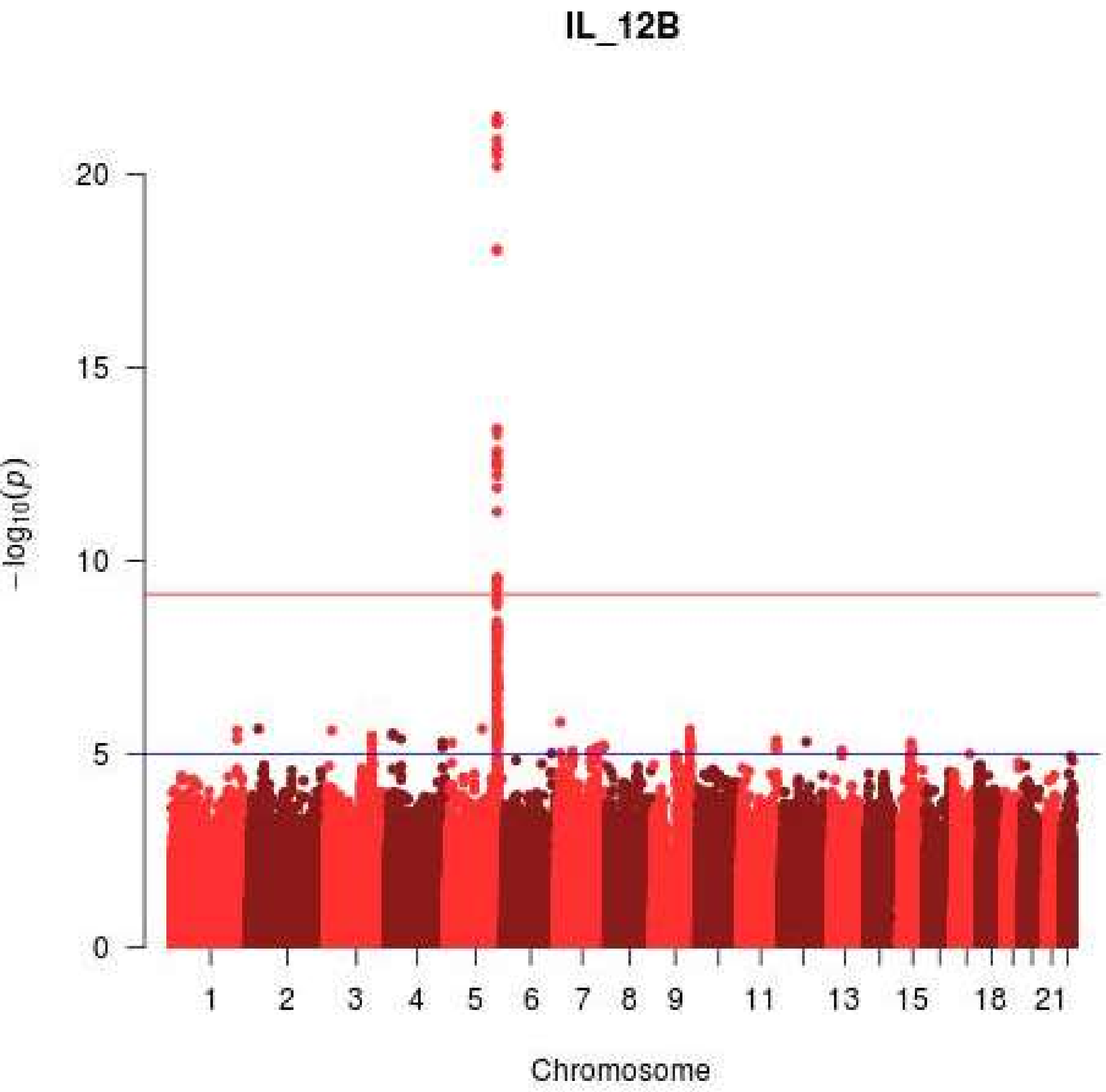
IL_12B
20
15
10
5
0
−log10(p)
1
2
3
4
5
6
7
8
9
11
13
15
18
21
Chromosome

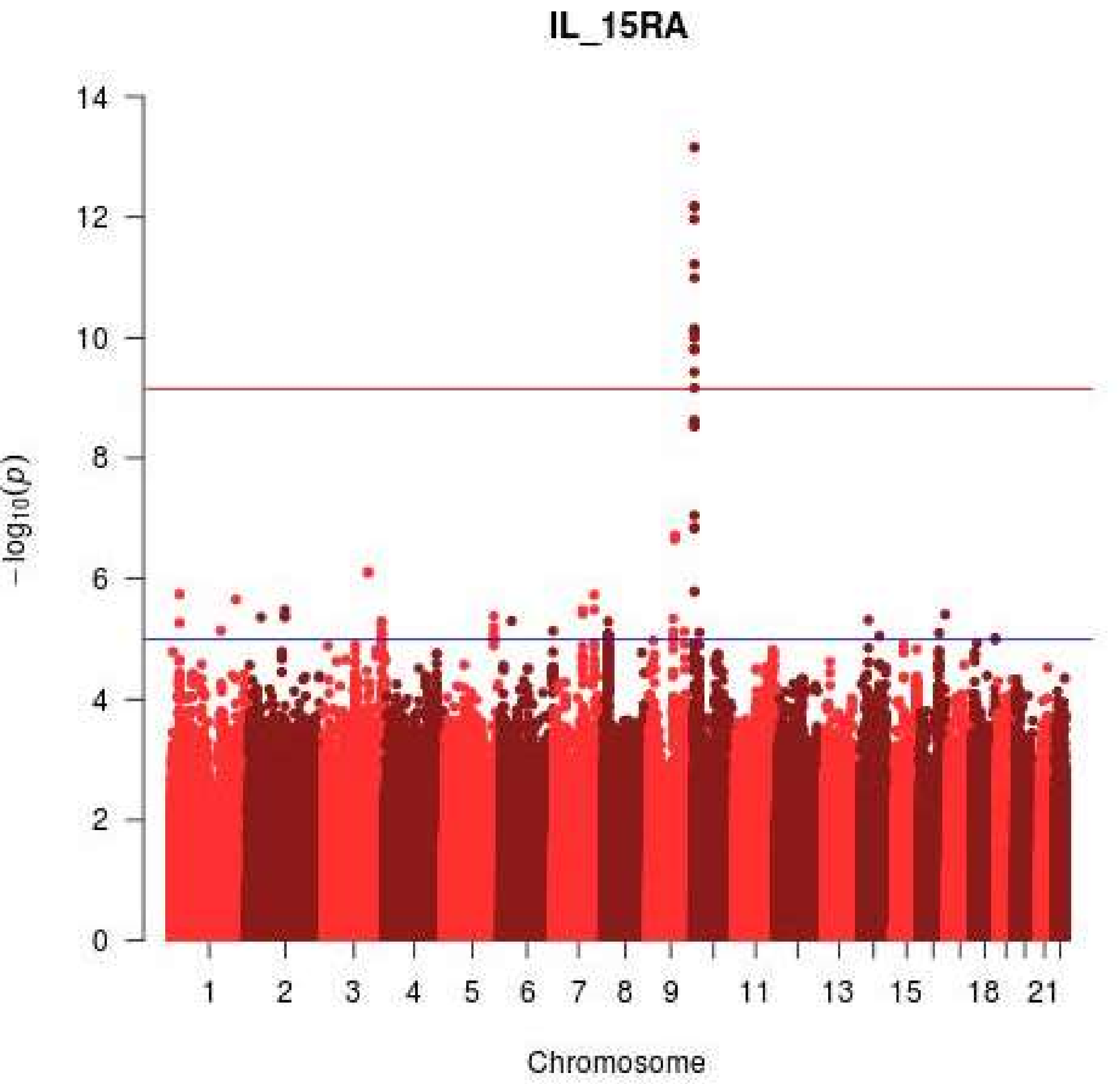

IL_15RA
14
12
10
8
6
4
2
0
$-\log_{10}(p)$
1
2
3
4
5
6
7
8
9
11
13
15
18
21
Chromosome

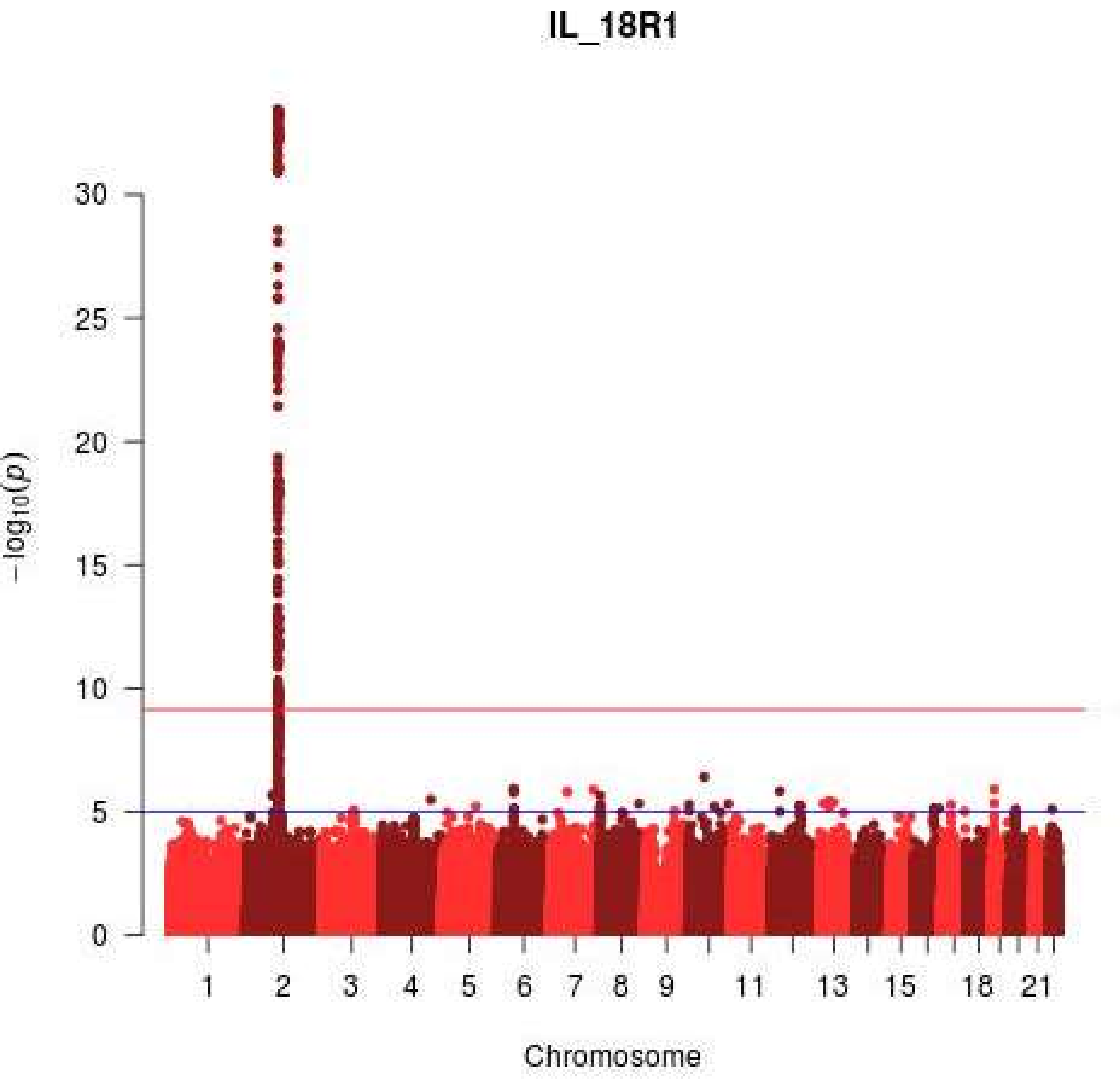
IL_18R1
30
25
20
15
10
5
0
$-\log_{10}(p)$
1
2
3
4
5
6
7
8
9
11
13
15
18
21
Chromosome

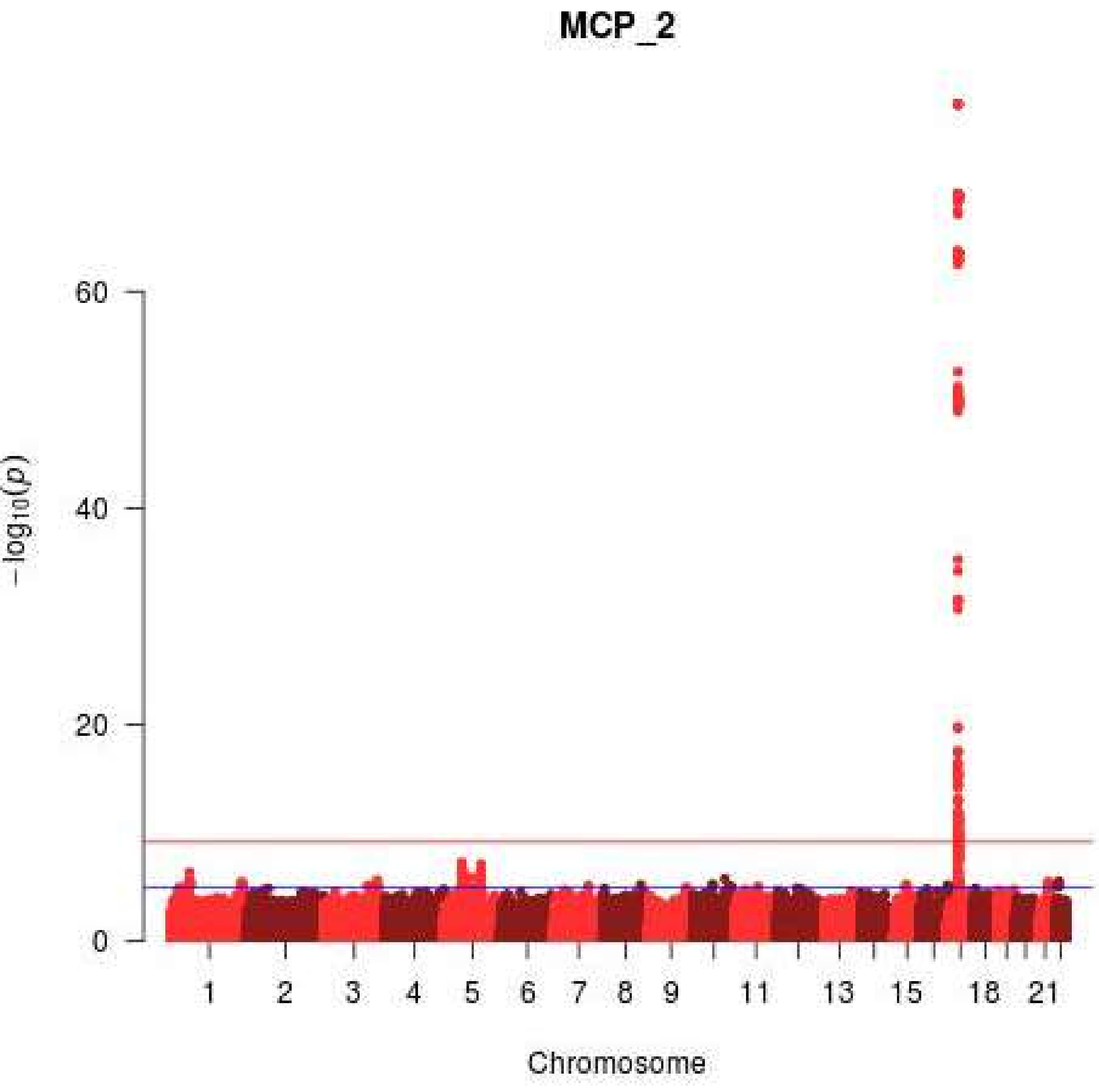
MCP_2
60
40
20
0
−log10(p)
1
2
3
4
5
6
7
8
9
11
13
15
18
21
Chromosome

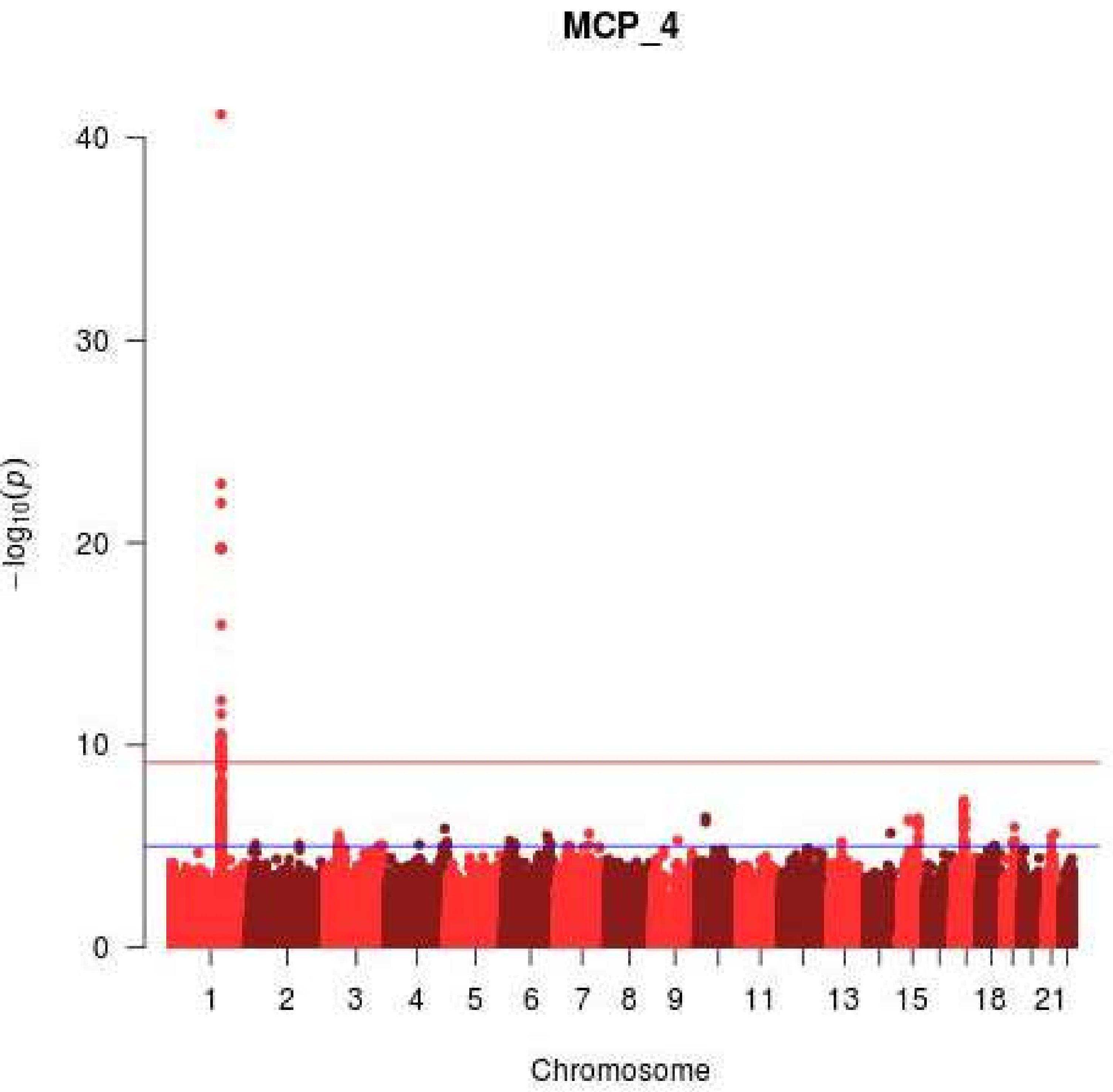

MCP_4
40
30
20
10
0
$-\log_{10}(p)$
1
2
3
4
5
6
7
8
9
11
13
15
18
21
Chromosome

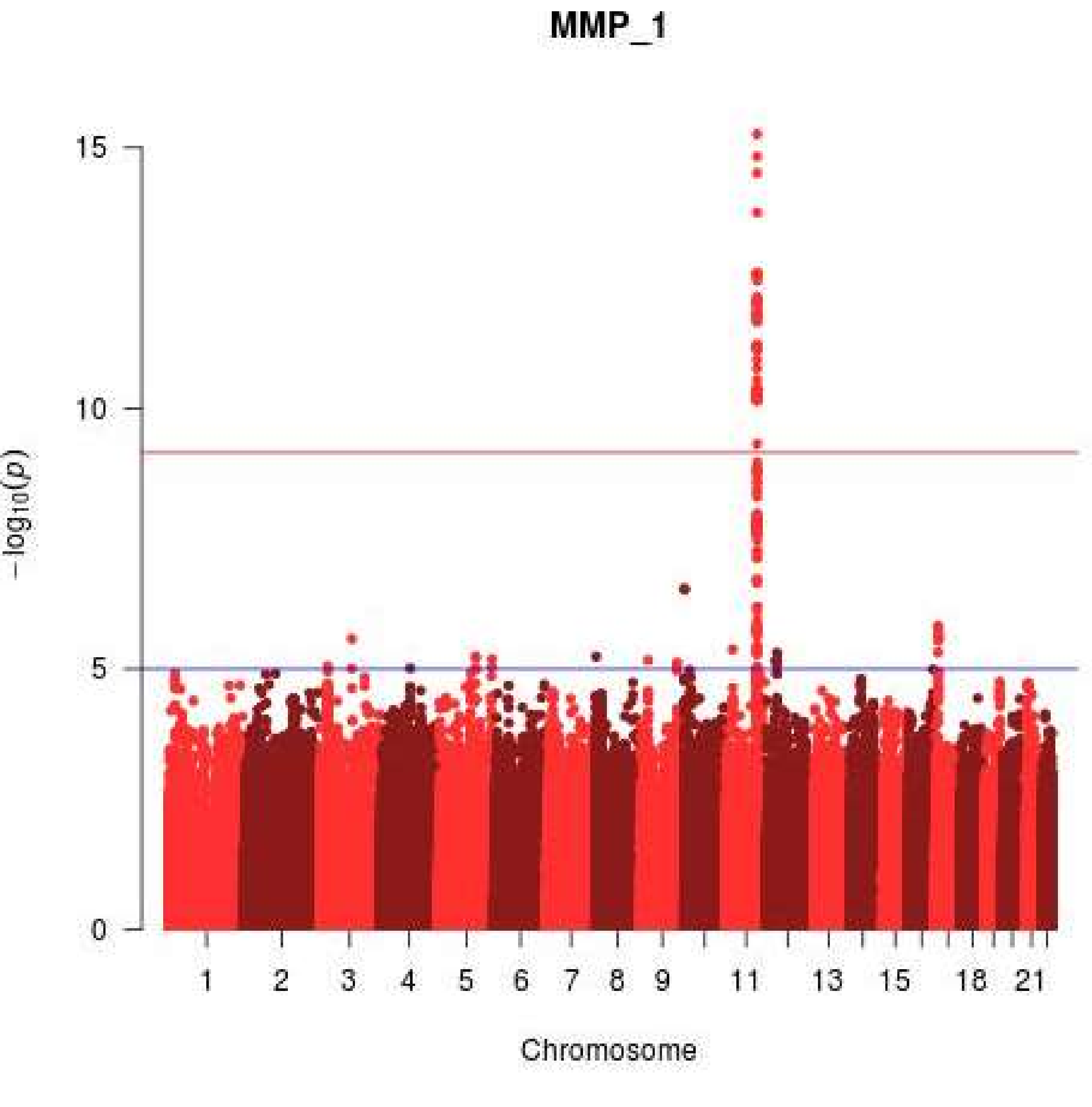

MMP_1
15
10
5
0
$-\log_{10}(p)$
1
2
3
4
5
6
7
8
9
11
13
15
18
21
Chromosome

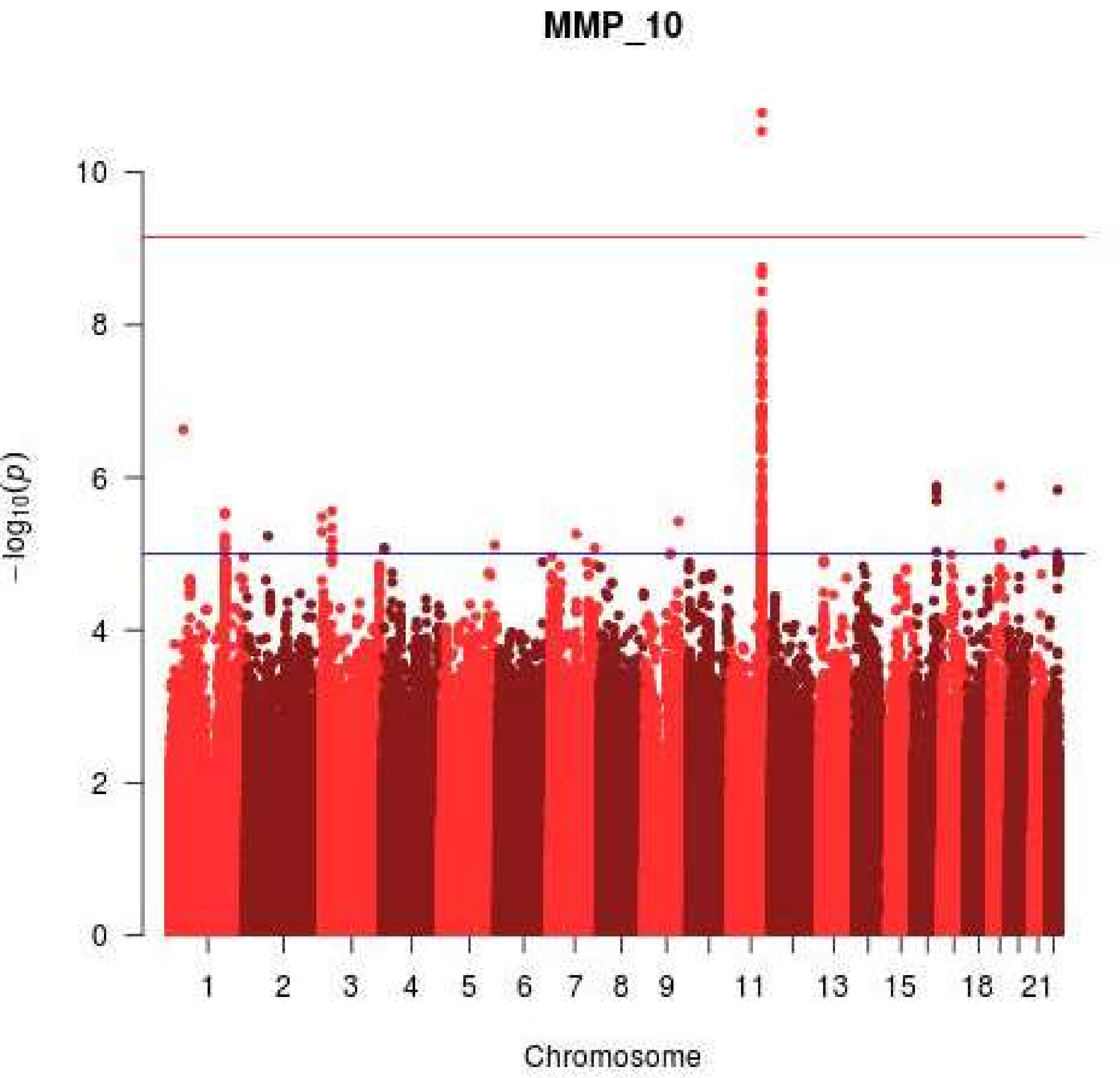
MMP_10
10
8
6
4
2
0
−log10(p)
1
2
3
4
5
6
7
8
9
11
13
15
18
21
Chromosome

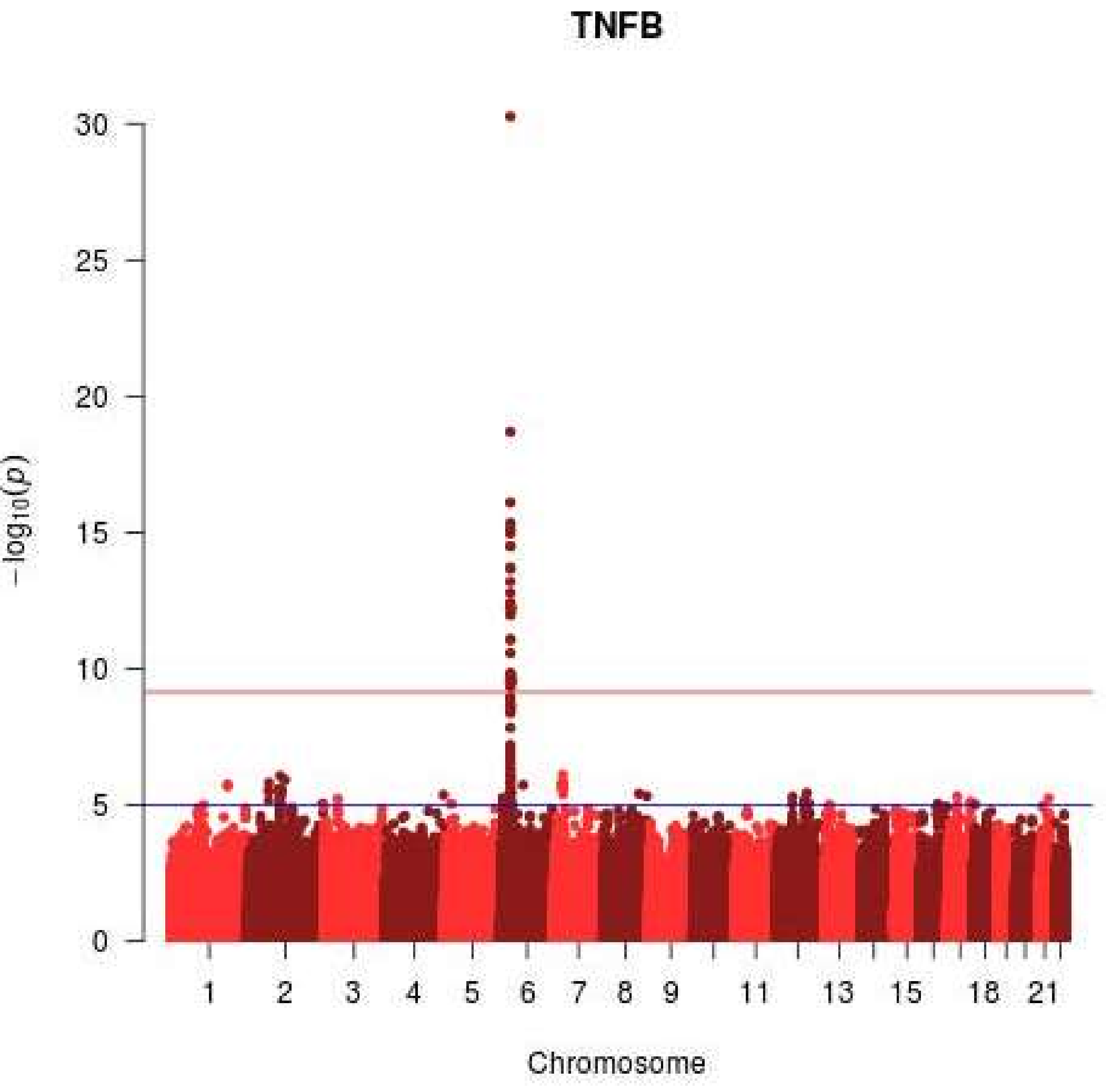
TNFB
30
25
20
15
10
5
0
$-\log_{10}(p)$
1
2
3
4
5
6
7
8
9
11
13
15
18
21
Chromosome

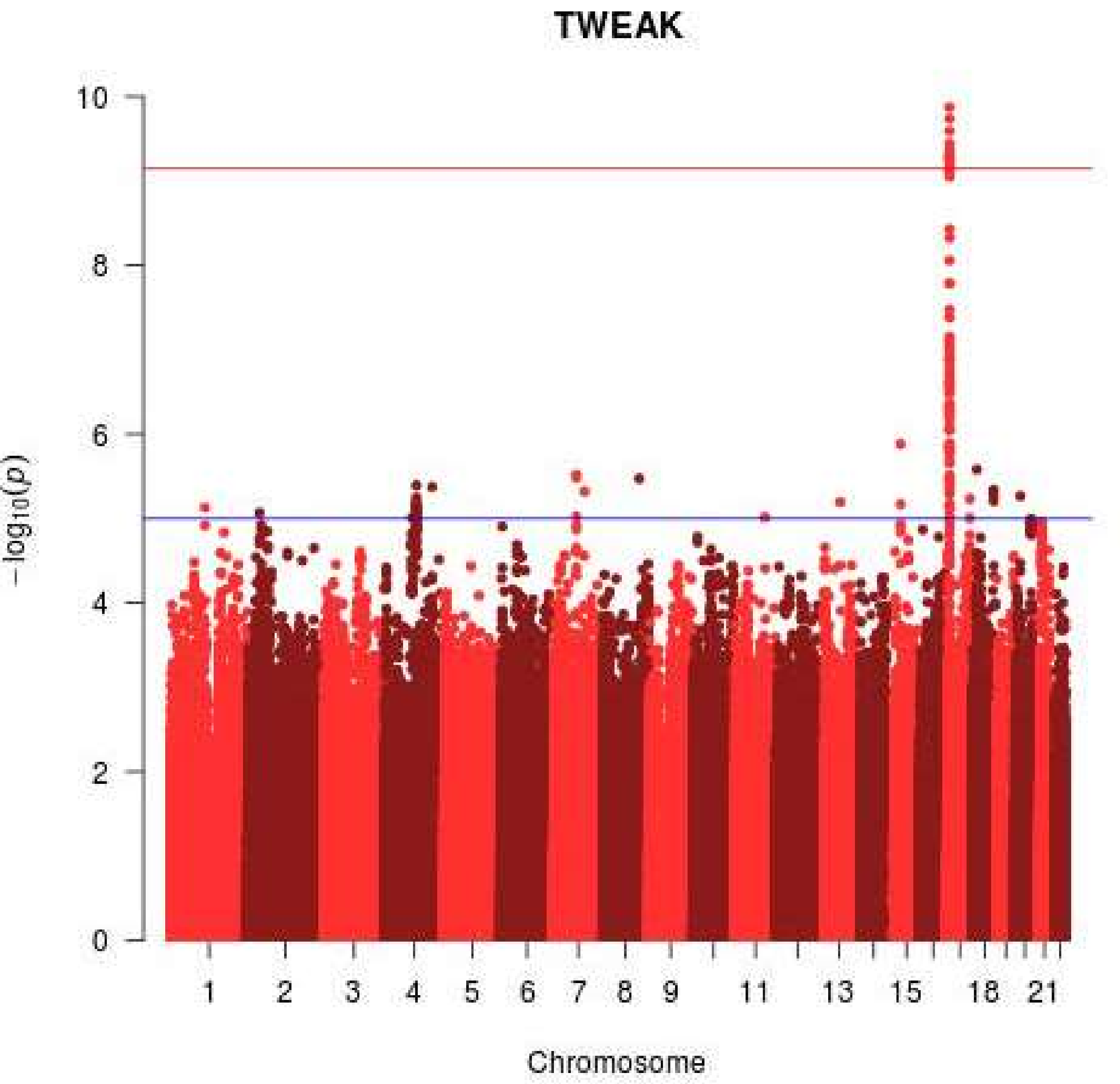
TWEAK
10
8
6
4
2
0
−log10(p)
1
2
3
4
5
6
7
8
9
11
13
15
18
21
Chromosome

Appendix 2

Q-Q Plots – Hillary *et al.*

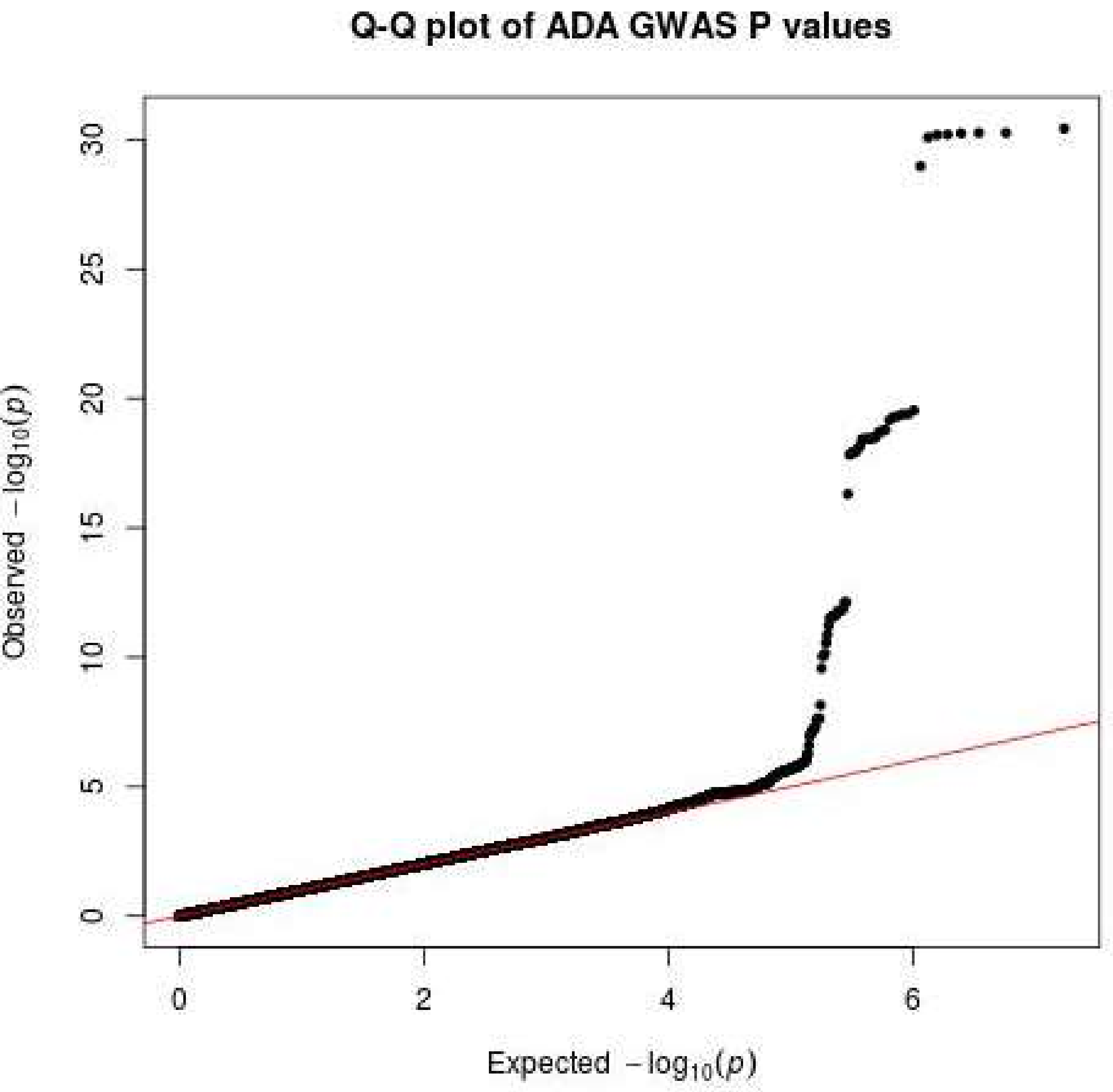

Q-Q plot of ADA GWAS P values
Observed $-\log_{10}(p)$
Expected $-\log_{10}(p)$
0
5
10
15
20
25
30
0
2
4
6

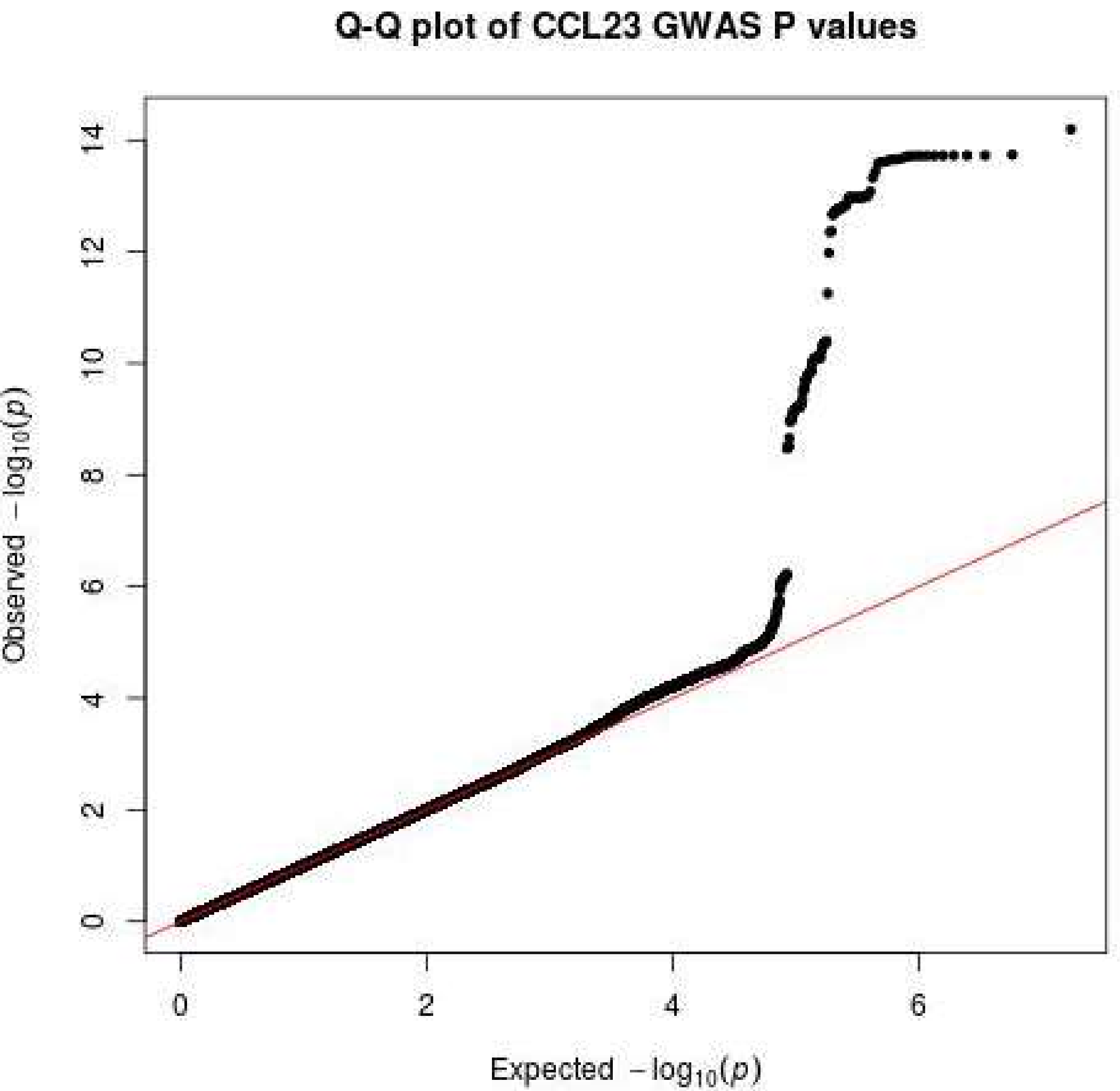
Q-Q plot of CCL23 GWAS P values
Observed $-\log_{10}(p)$
Expected $-\log_{10}(p)$
0
2
4
6
8
10
12
14
0
2
4
6

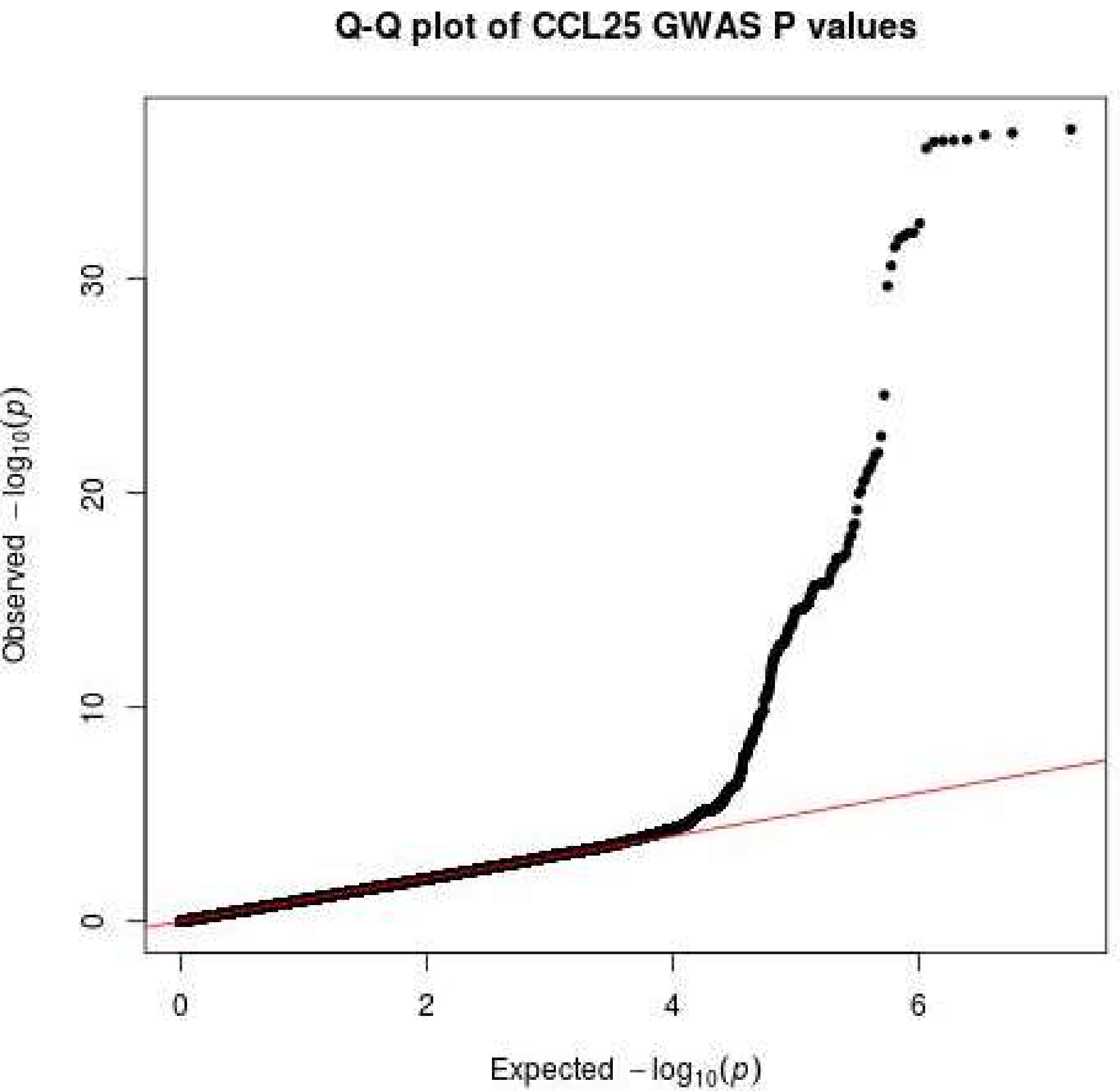
Q-Q plot of CCL25 GWAS P values
Observed $-\log_{10}(p)$
Expected $-\log_{10}(p)$
0
10
20
30
0
2
4
6

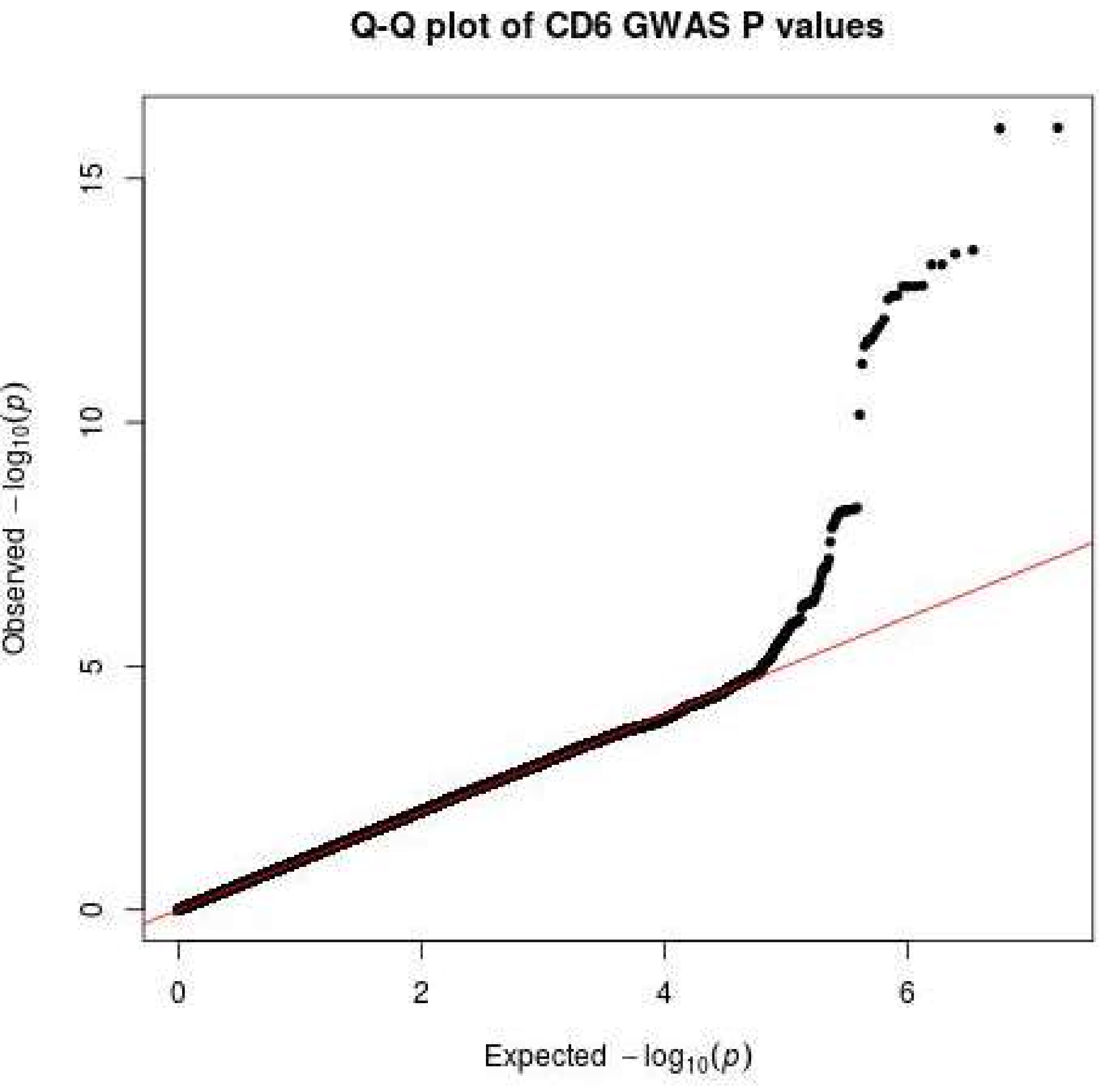
Q-Q plot of CD6 GWAS P values
Observed −log10(p)
Expected −log10(p)
0
5
10
15
0
2
4
6

Q-Q plot of CD40 GWAS P values

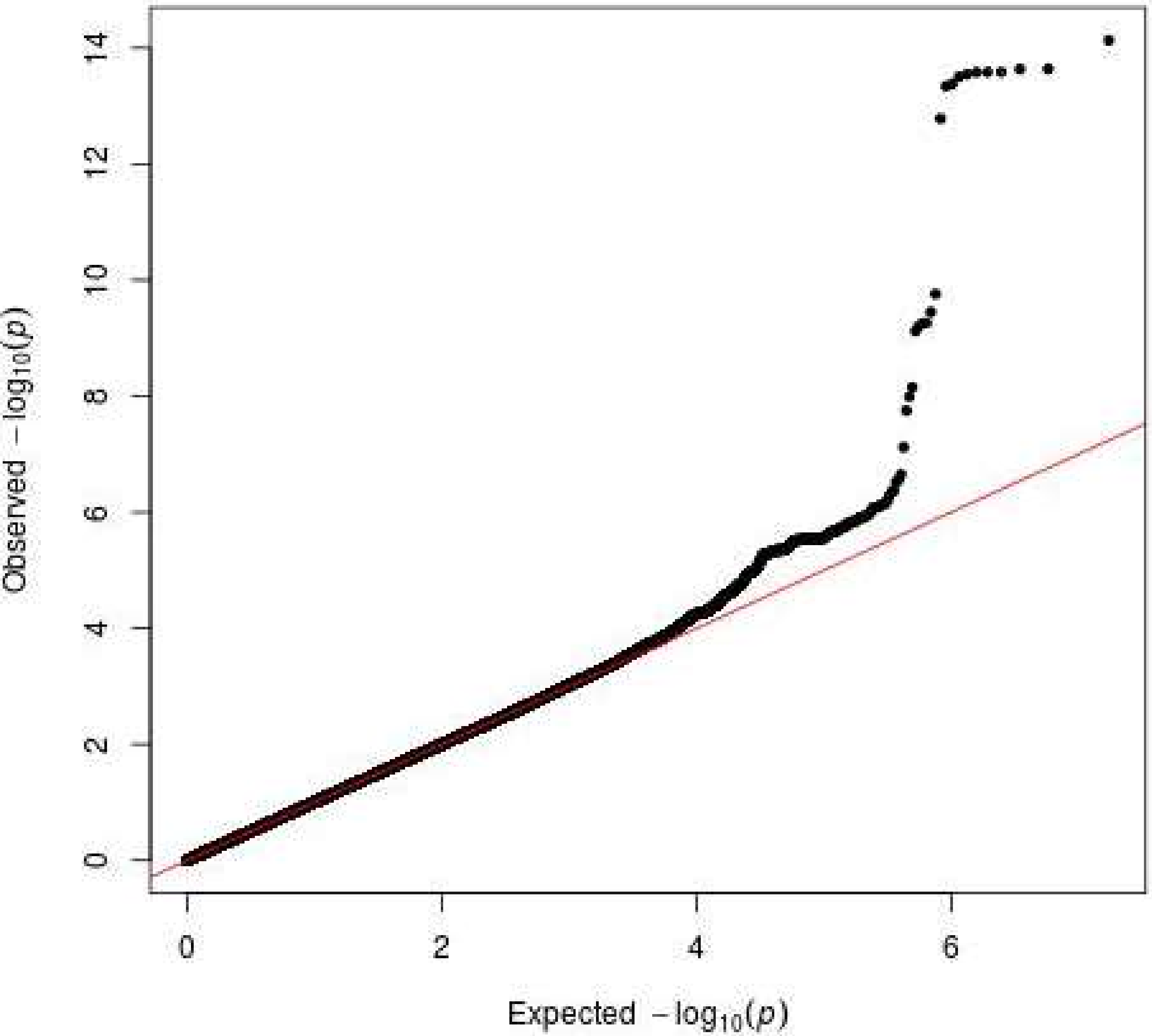

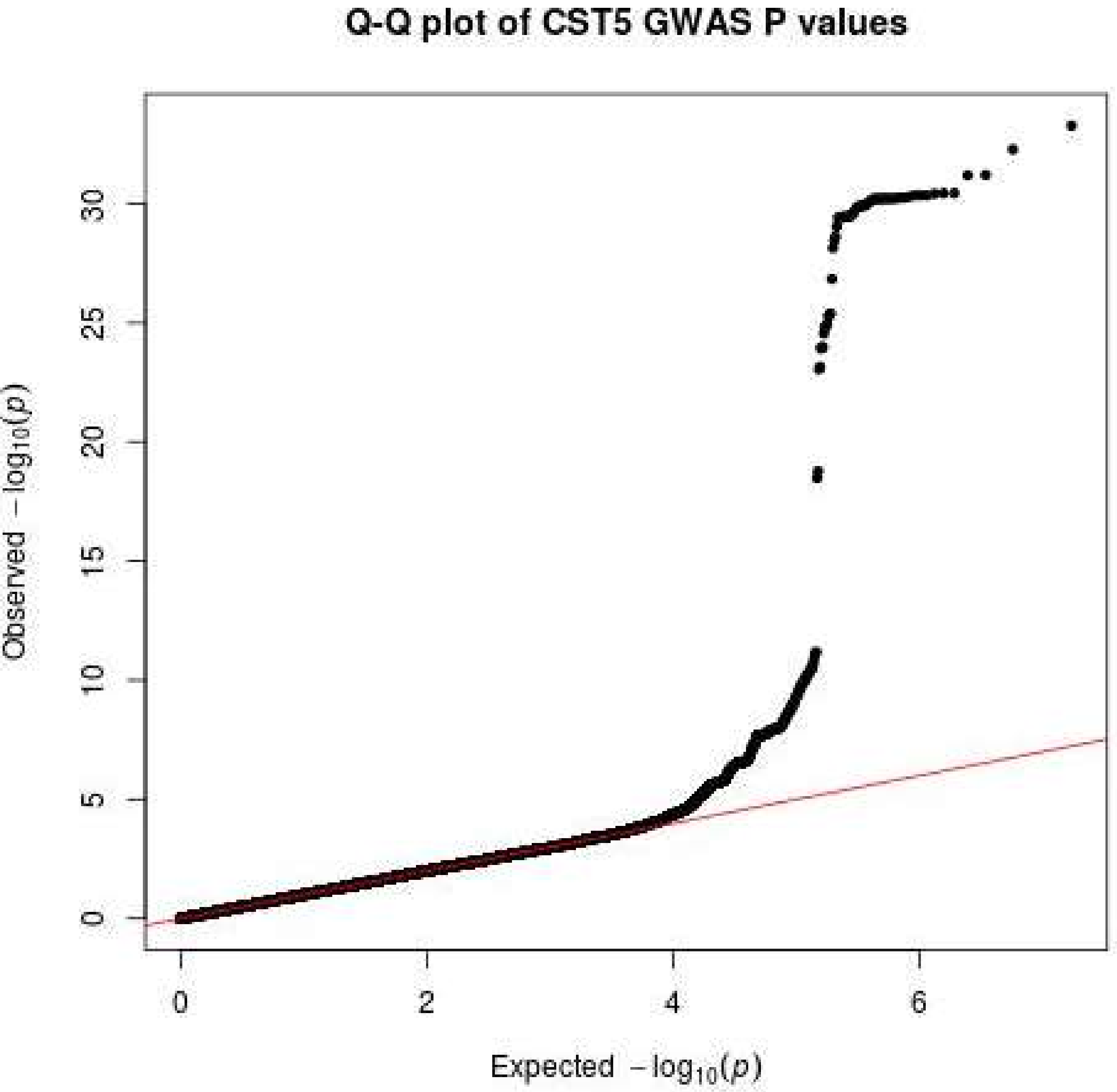

Q-Q plot of CST5 GWAS P values
Observed $-\log_{10}(p)$
0
5
10
15
20
25
30
Expected $-\log_{10}(p)$
0
2
4
6

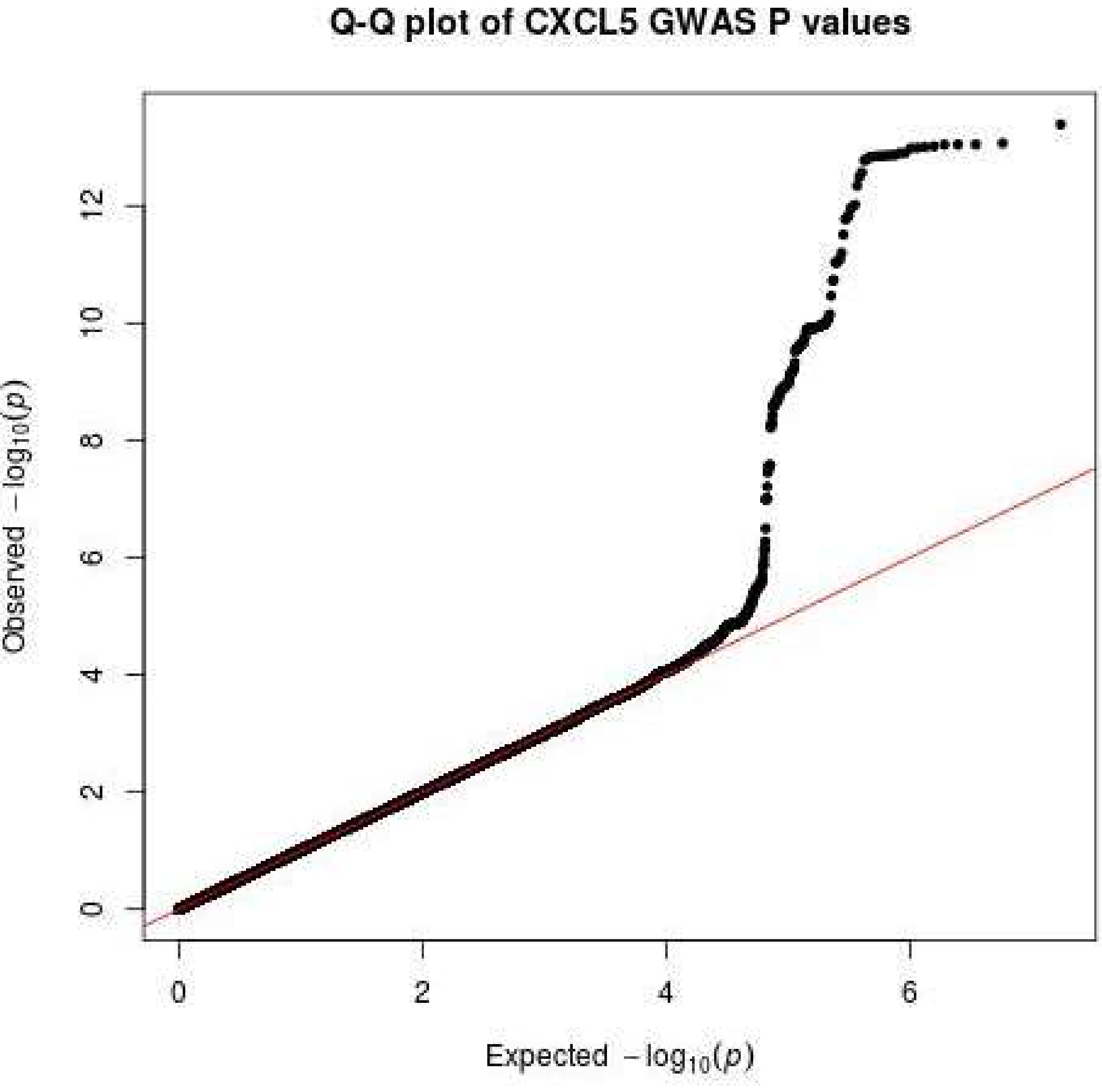
Q-Q plot of CXCL5 GWAS P values
Observed − log10(p)
Expected − log10(p)
0
2
4
6
8
10
12
0
2
4
6

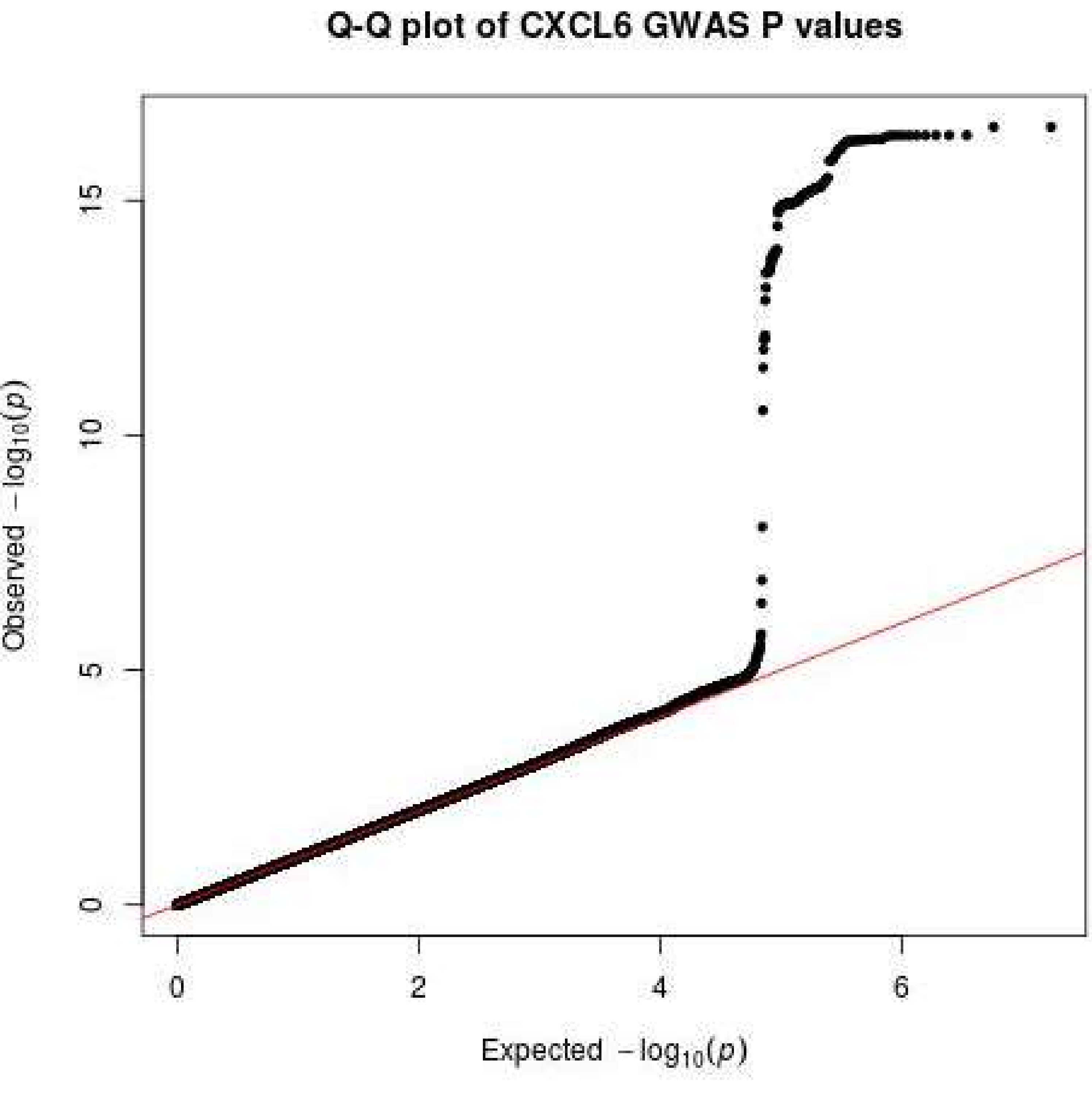

Q-Q plot of CXCL6 GWAS P values
Observed $-\log_{10}(p)$
Expected $-\log_{10}(p)$
0
5
10
15
0
2
4
6

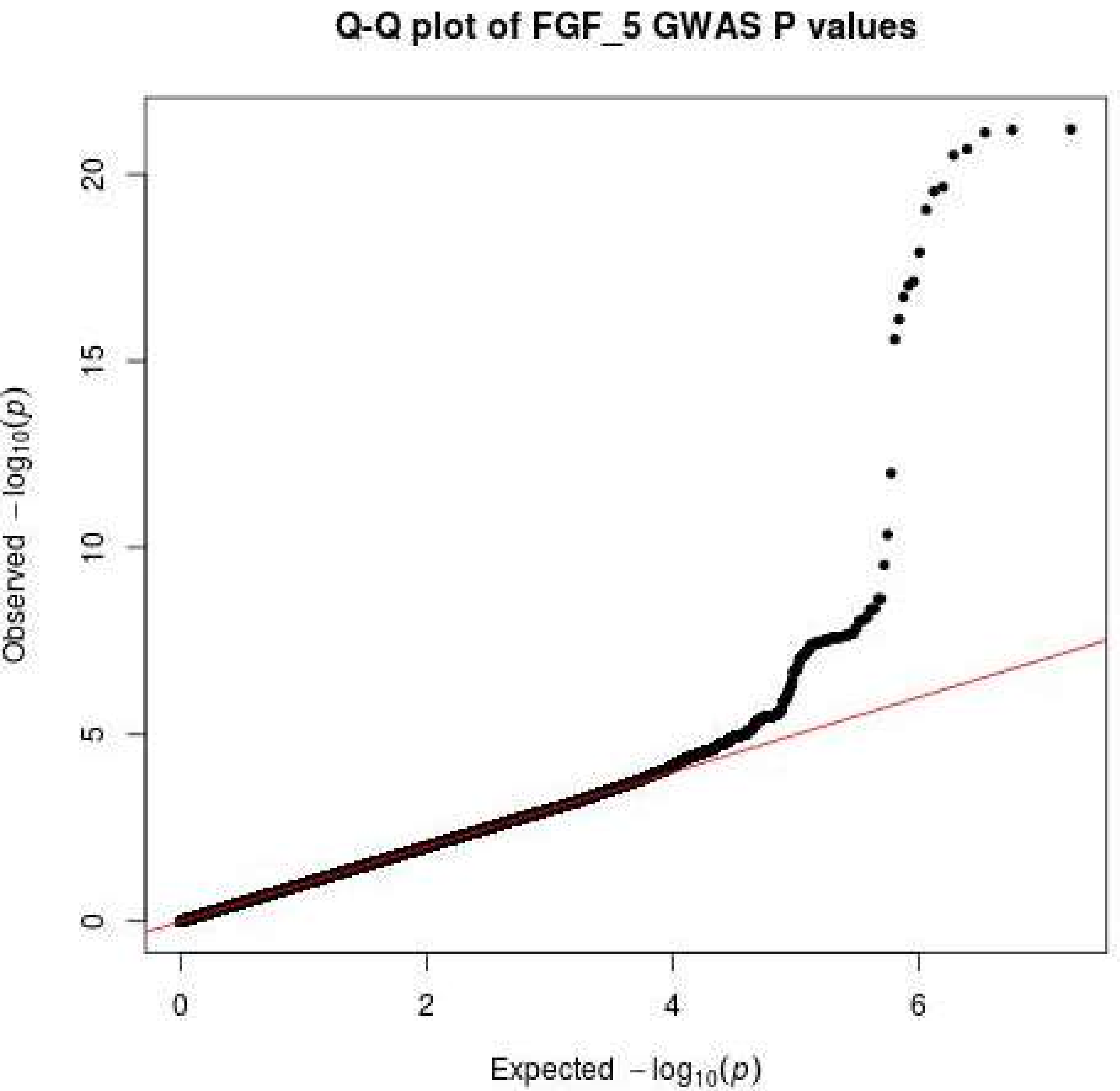
Q-Q plot of FGF_5 GWAS P values
Observed $-\log_{10}(p)$
Expected $-\log_{10}(p)$
0
5
10
15
20
0
2
4
6

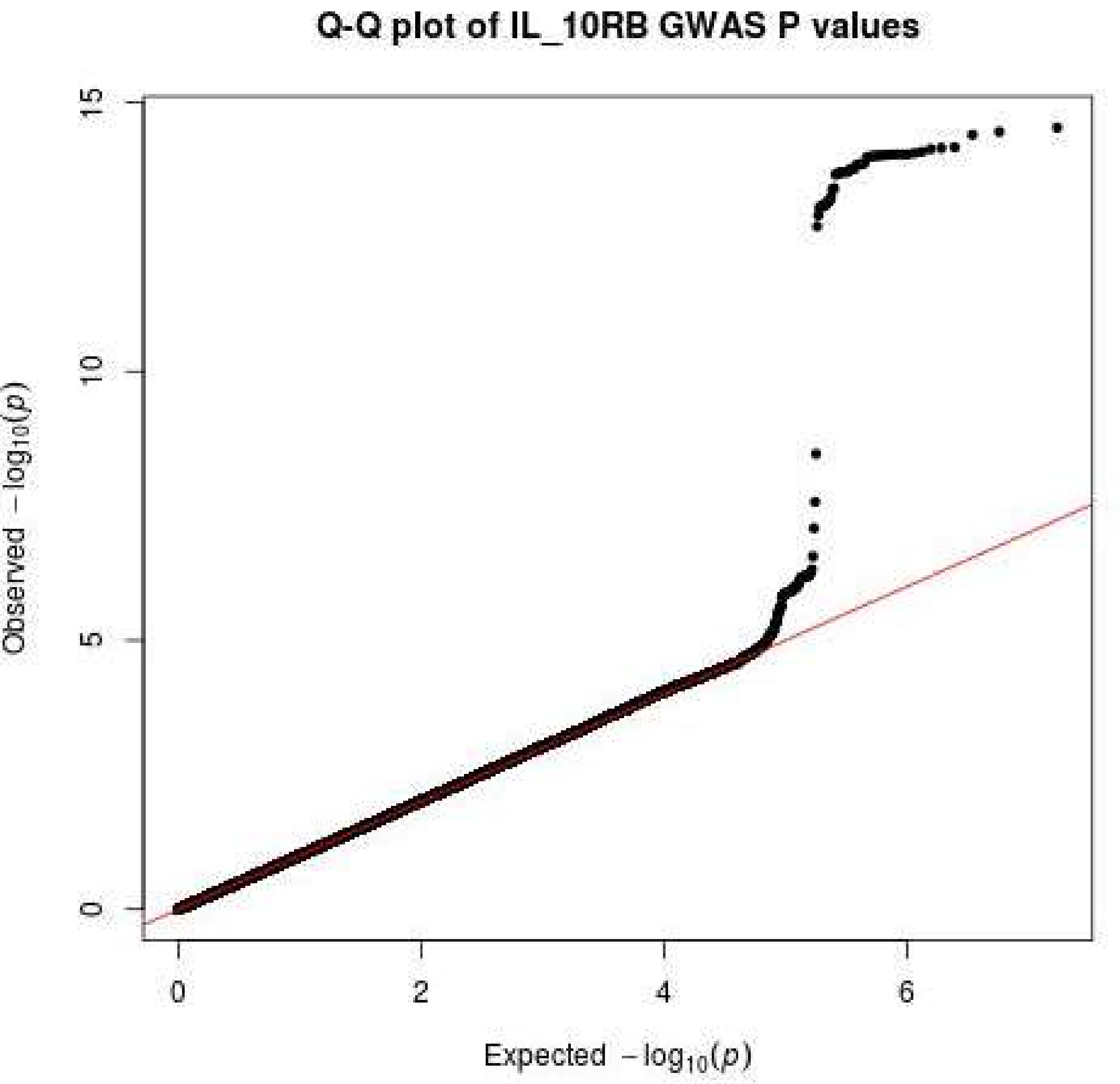
Q-Q plot of IL_10RB GWAS P values
Observed −log10(p)
Expected −log10(p)
0
5
10
15
0
2
4
6

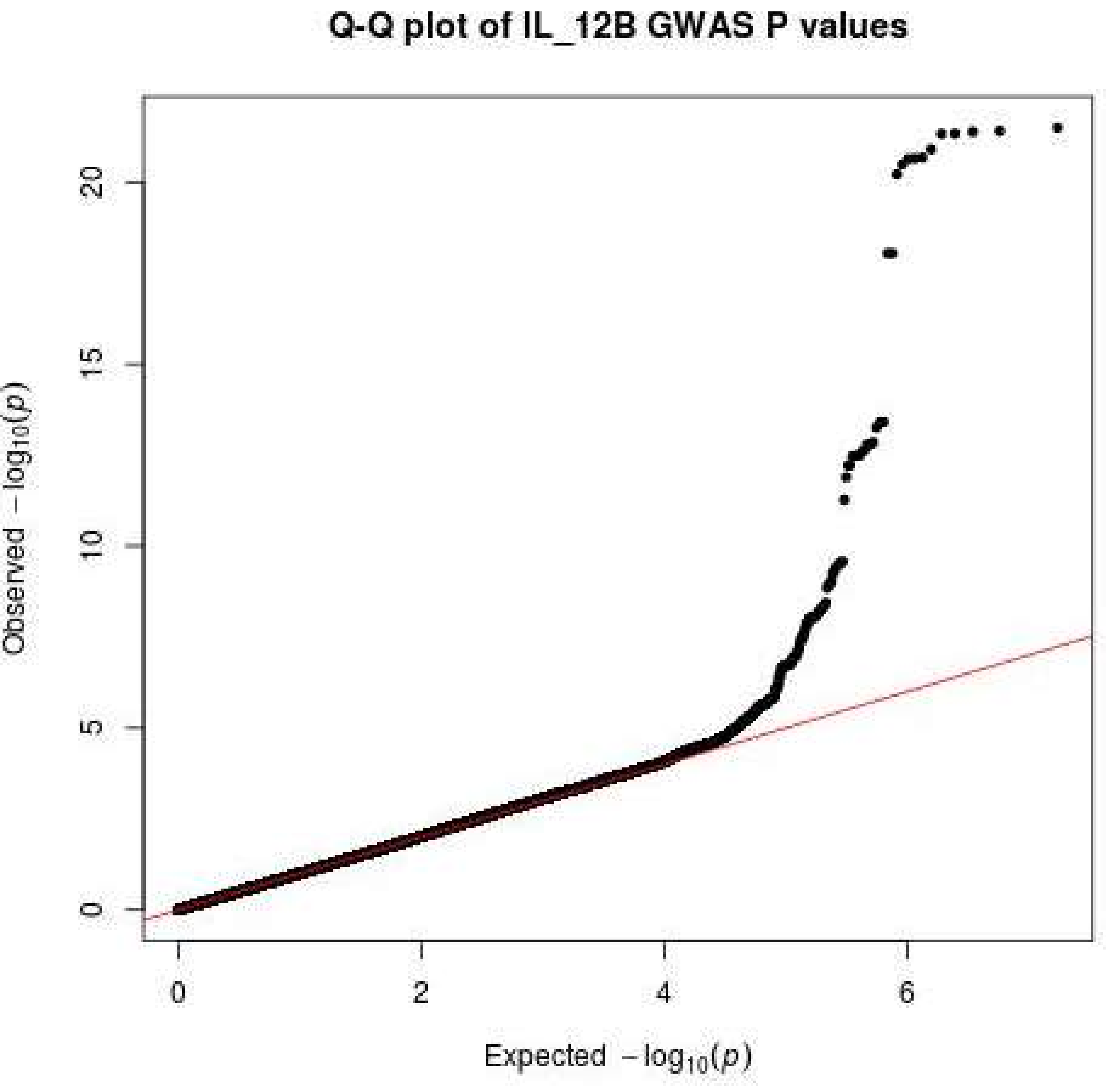

Q-Q plot of IL_12B GWAS P values
Observed −log10(p)
Expected −log10(p)
0
5
10
15
20
0
2
4
6

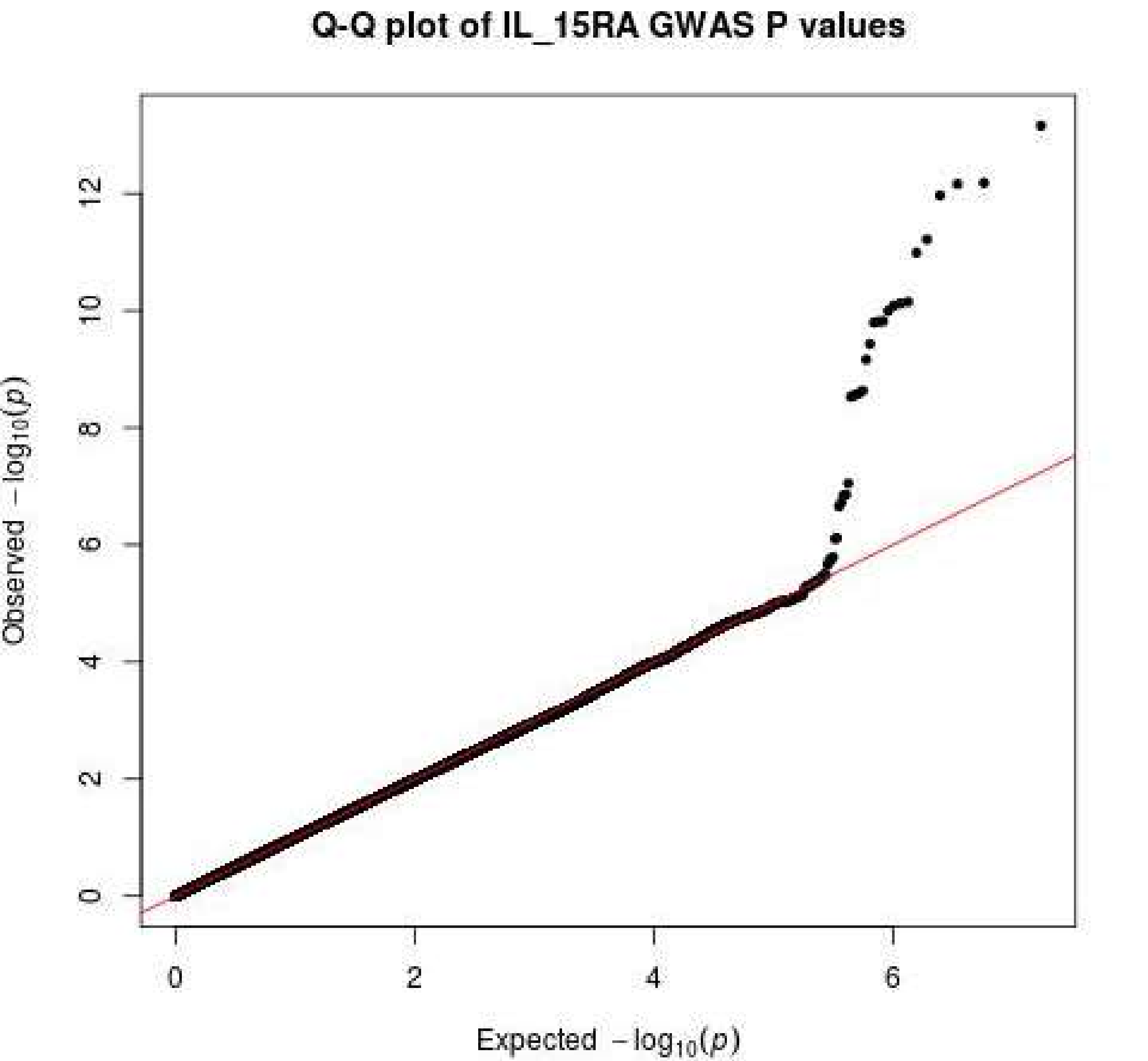

Q-Q plot of IL_15RA GWAS P values
0
2
4
6
8
10
12
Observed $-\log_{10}(p)$
0
2
4
6
Expected $-\log_{10}(p)$

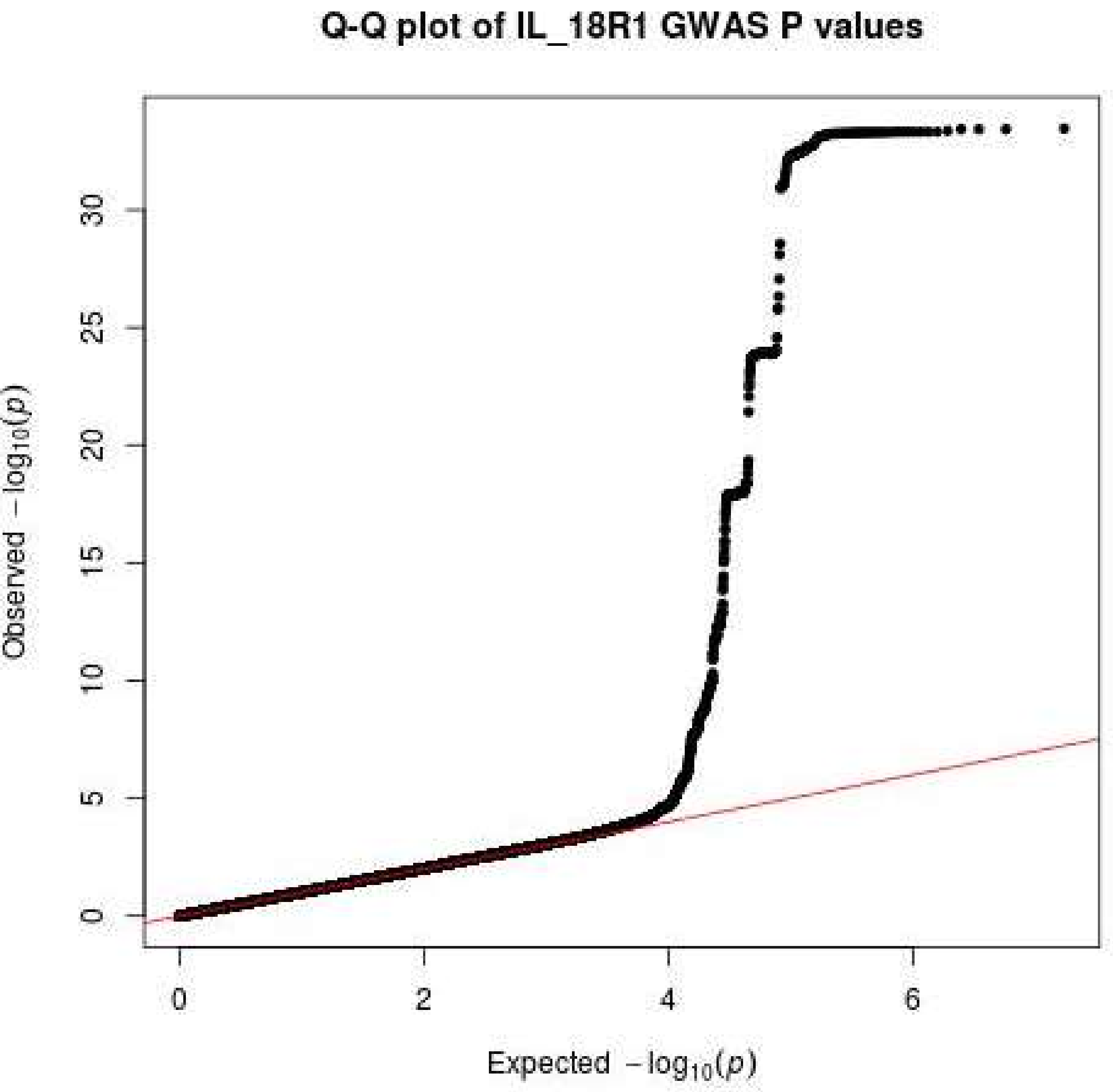
Q-Q plot of IL_18R1 GWAS P values
Observed −log10(p)
Expected −log10(p)
0
5
10
15
20
25
30
0
2
4
6

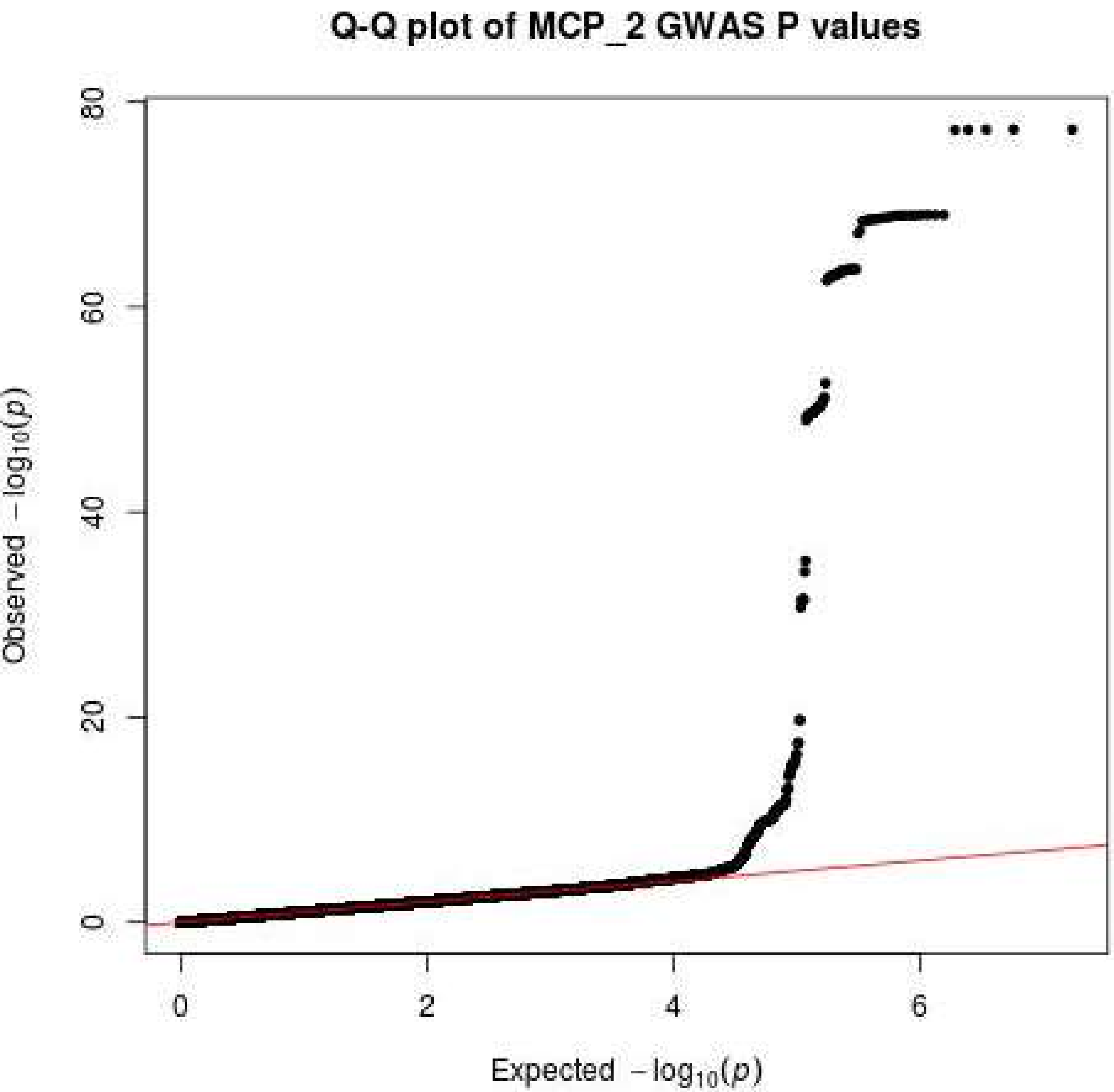

Q-Q plot of MCP_2 GWAS P values
80
60
40
20
0
Observed −log10(p)
0
2
4
6
Expected −log10(p)

Q-Q plot of MCP_4 GWAS P values

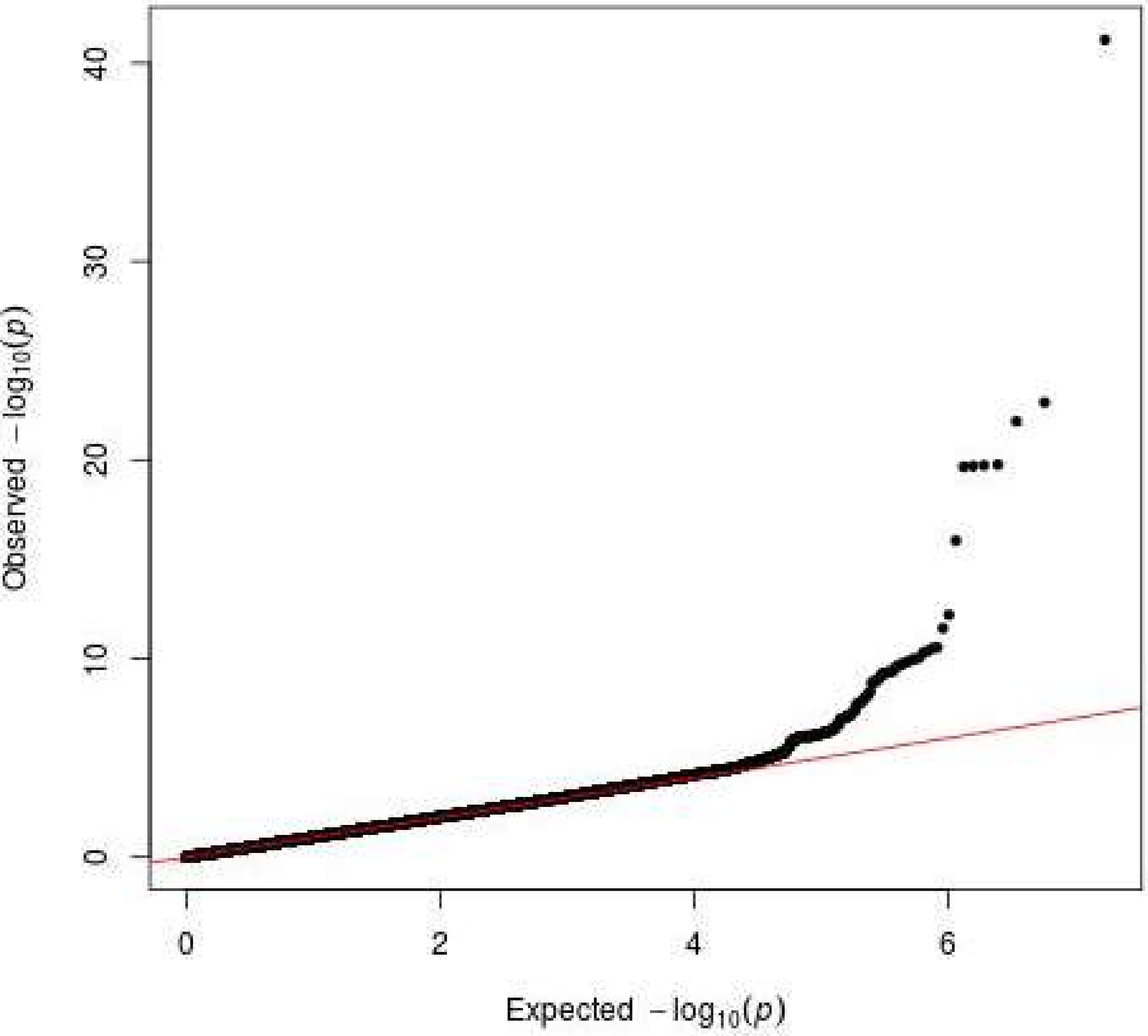

Q-Q plot of MMP_1 GWAS P values

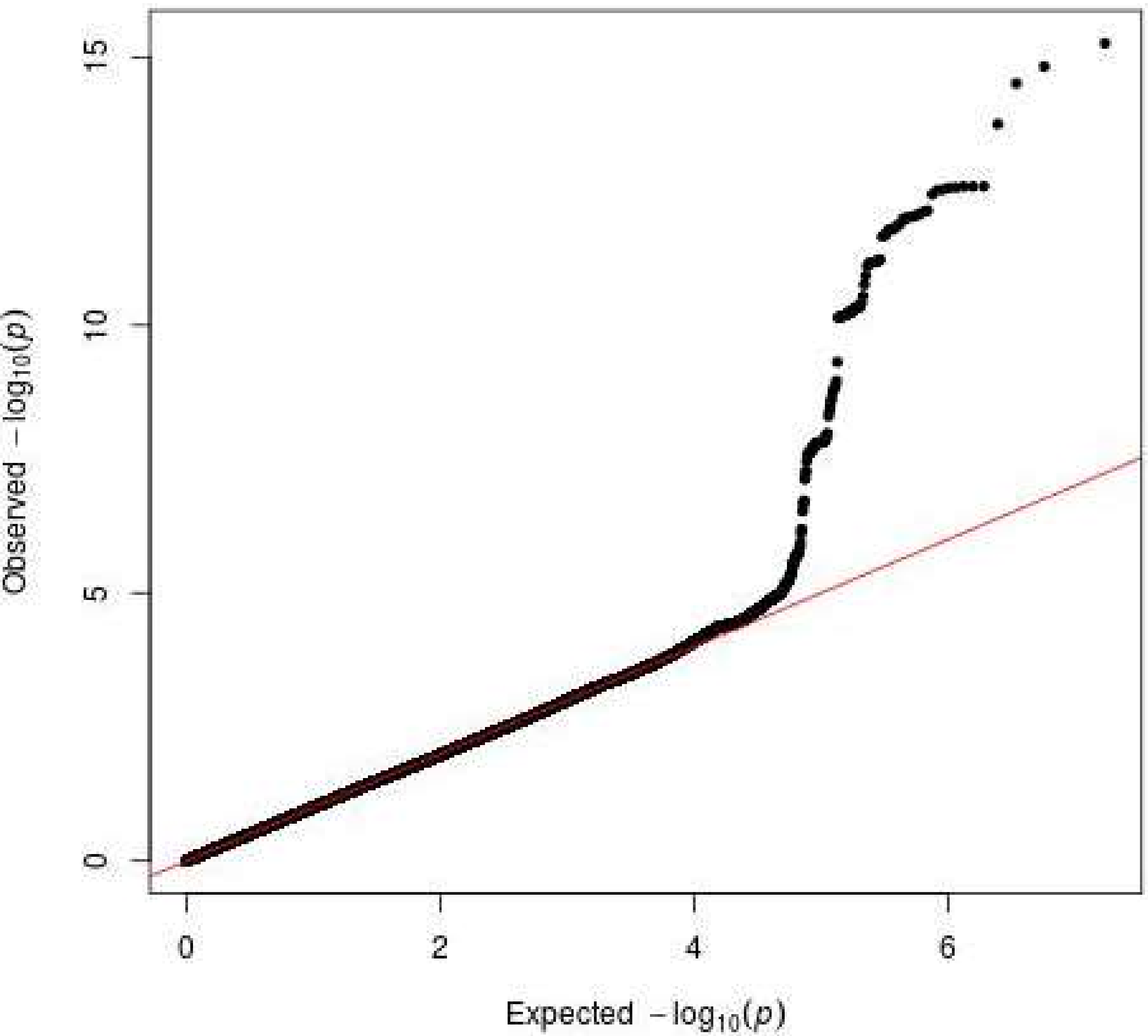

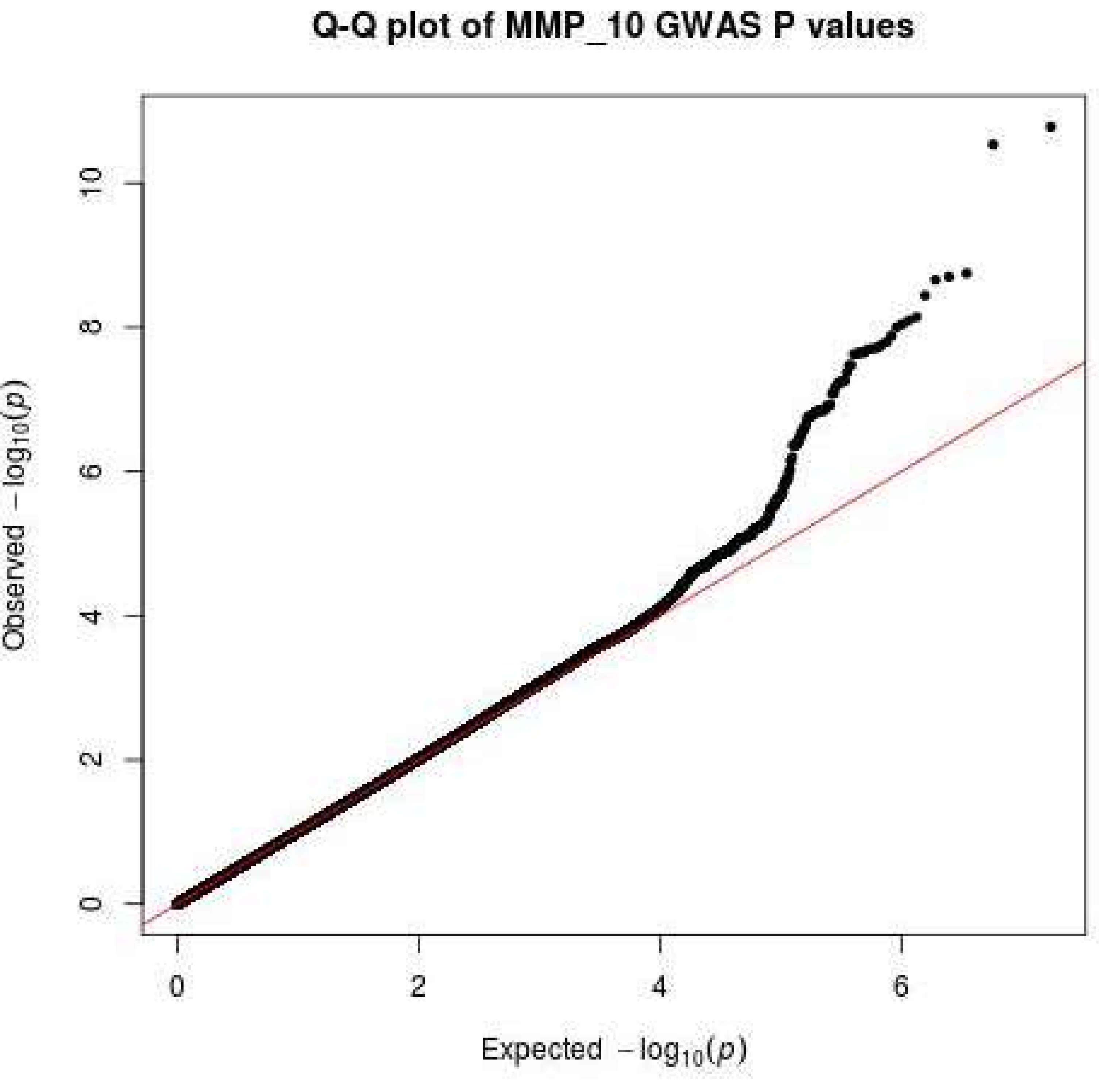

Q-Q plot of MMP_10 GWAS P values
Observed −log10(p)
Expected −log10(p)
0
2
4
6
8
10
0
2
4
6

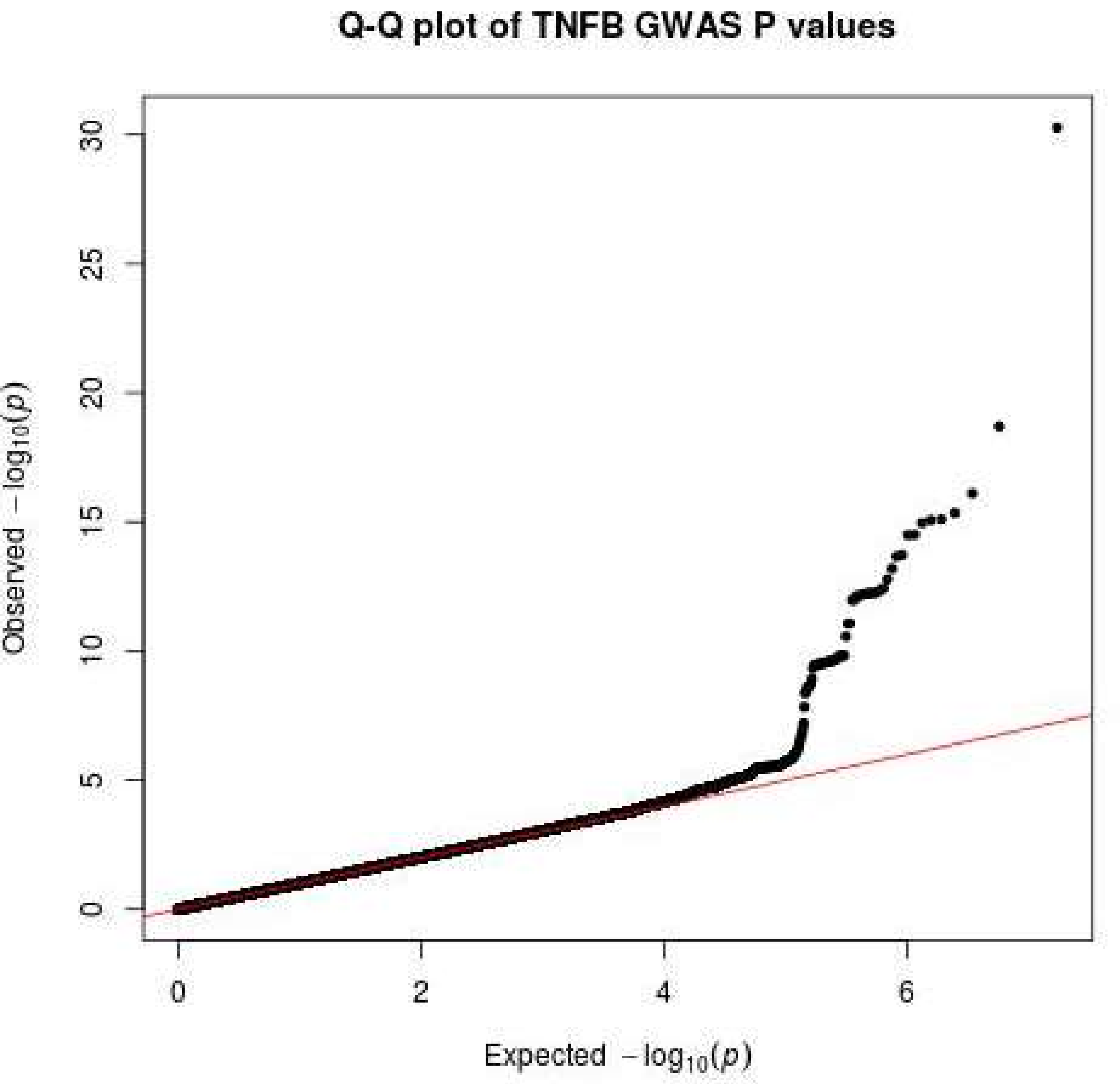

Q-Q plot of TNFB GWAS P values
Observed $-\log_{10}(p)$
Expected $-\log_{10}(p)$
0
5
10
15
20
25
30
0
2
4
6

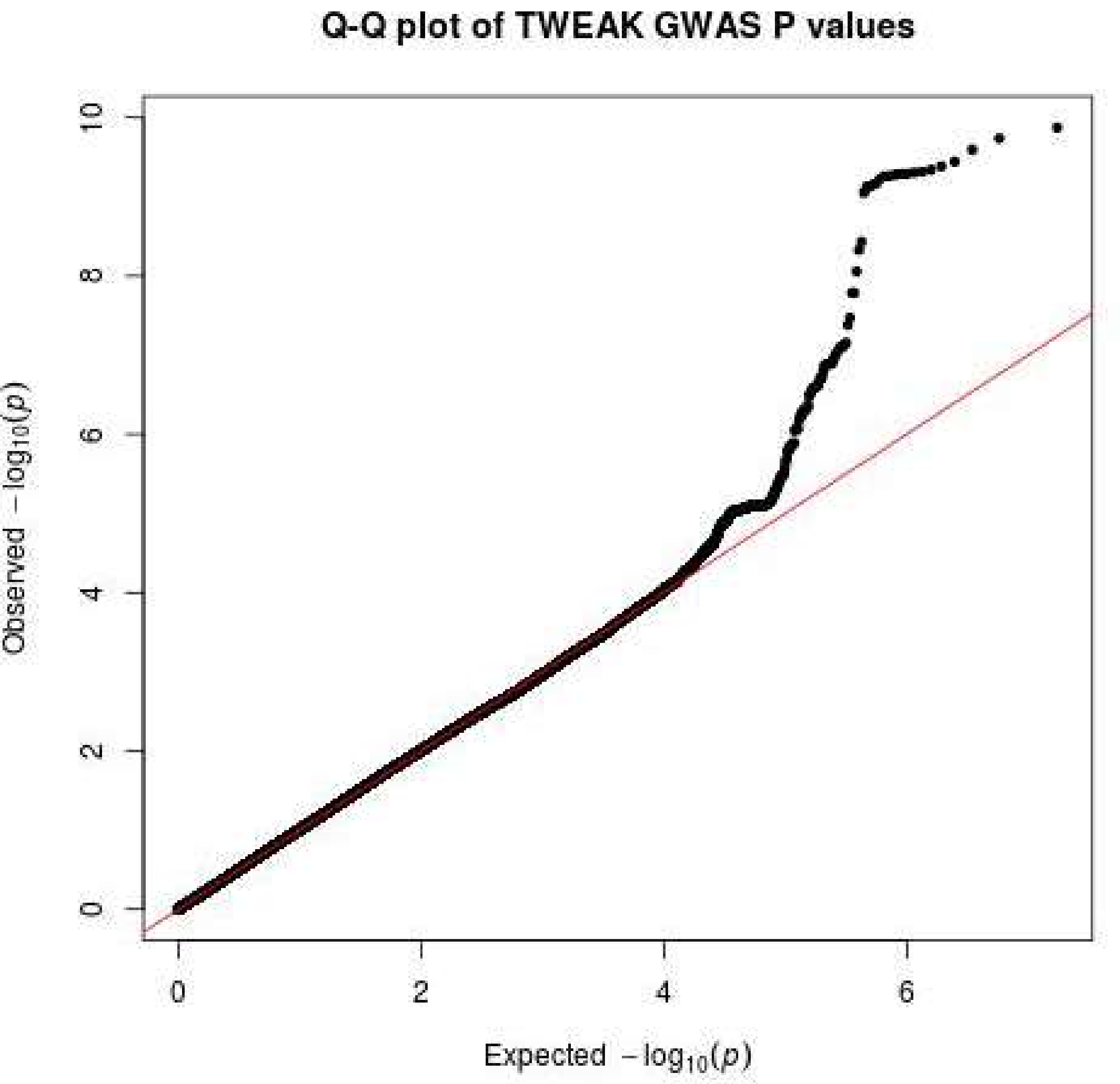

Q-Q plot of TWEAK GWAS P values
Observed $-\log_{10}(p)$
Expected $-\log_{10}(p)$
0
2
4
6
8
10
0
2
4
6
