# Supplementary material for: Multi-method genome- and epigenome-wide studies of inflammatory protein levels in healthy older adults
Source: Genome Med. 2020 Jul 8;12:60. doi: 10.1186/s13073-020-00754-1 (PMC7346642; doi:10.1186/s13073-020-00754-1)
Supplement: Supplementary file 4 — Additional file 4: Supplementary Figures. Correlation between the 13 proteins with significant pQTLs as identified by ordinary least squares and Bayesian penalised regression. (Figure S1). Correlation between heritability estimates for circulating inflammatory protein biomarkers from present study and that of Ahsan et al. The protein with the greatest discordance between studies (MMP-1) is annotated. (Figure S2). Correlation between the 3 proteins with significant CpG associations as identified across ordinary least squares model, mixed model and Bayesian penalised regression approaches. (Figure S3). Tissue-specific expression of genes annotated to CpGs associated with CCL11 levels at P < 1 × 10− 5. Differential expression was observed in kidney, adipose and breast tissue. (Figure S4). Tissue-specific expression of genes annotated to CpGs associated with IL18R1 levels at P < 1 × 10− 5. Differential expression was observed in pancreatic, hippocampal and substantia nigra tissue. (Figure S5). Tissue-specific expression of genes annotated to CpGs associated with CXCL9 levels at P < 1 × 10^–5^. No tissue-specific expression was observed. (Figure S6). Miami plot for IL18R1 which exhibited both genome-wide significant SNP and genome-wide significant CpG associations. The top half of the plot (skyline) shows the results from the GWAS on protein levels, whereas the bottom half (waterfront) shows the results from the EWAS. IL18R1 (chromosome 2: 102,311,529-102,398,775). (Figure S7). [file 13073_2020_754_MOESM4_ESM.pdf]

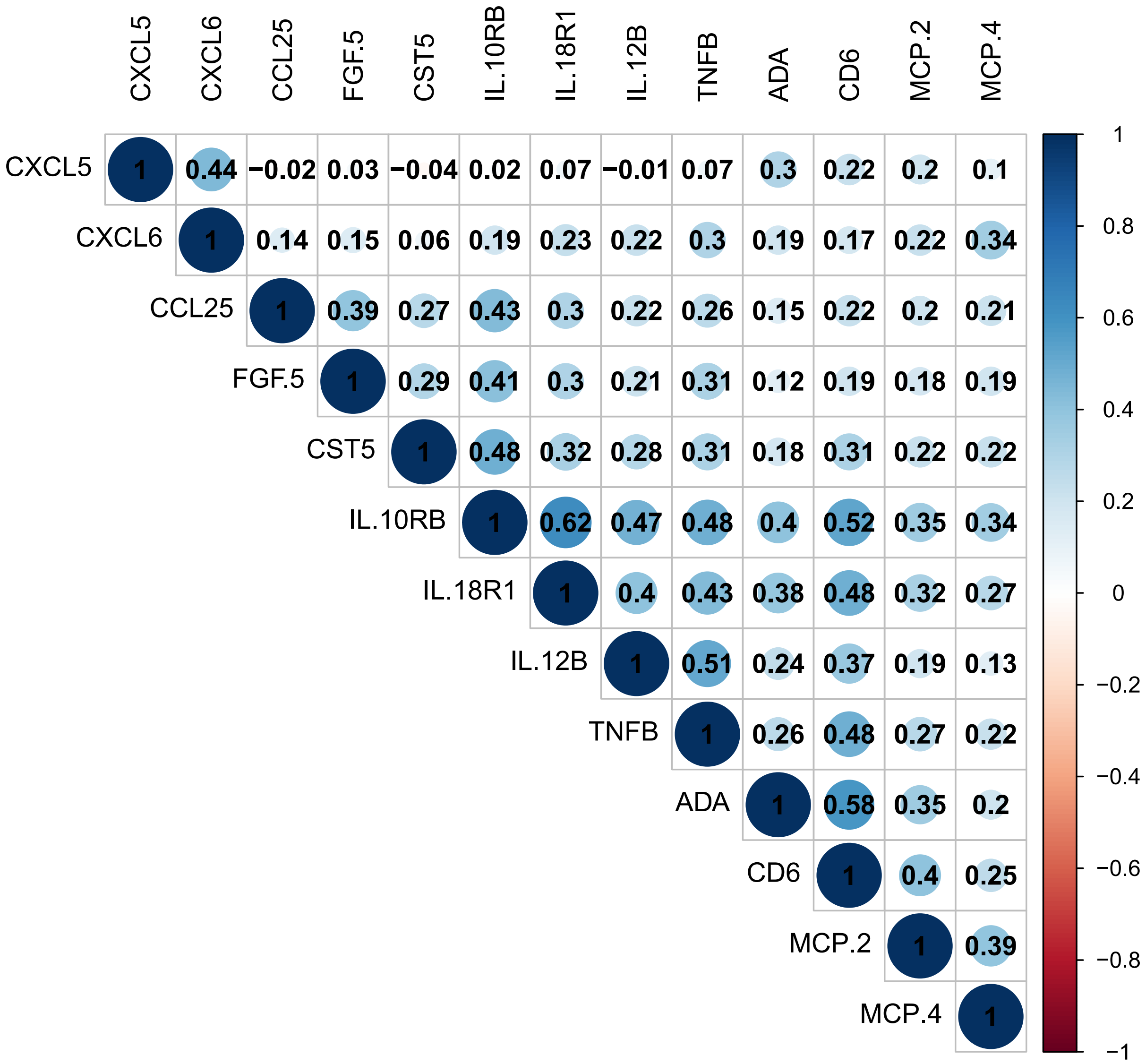

Fig. S1. Correlation between the 13 proteins with significant pQTLs as identified by ordinary least squares and Bayesian penalised regression.

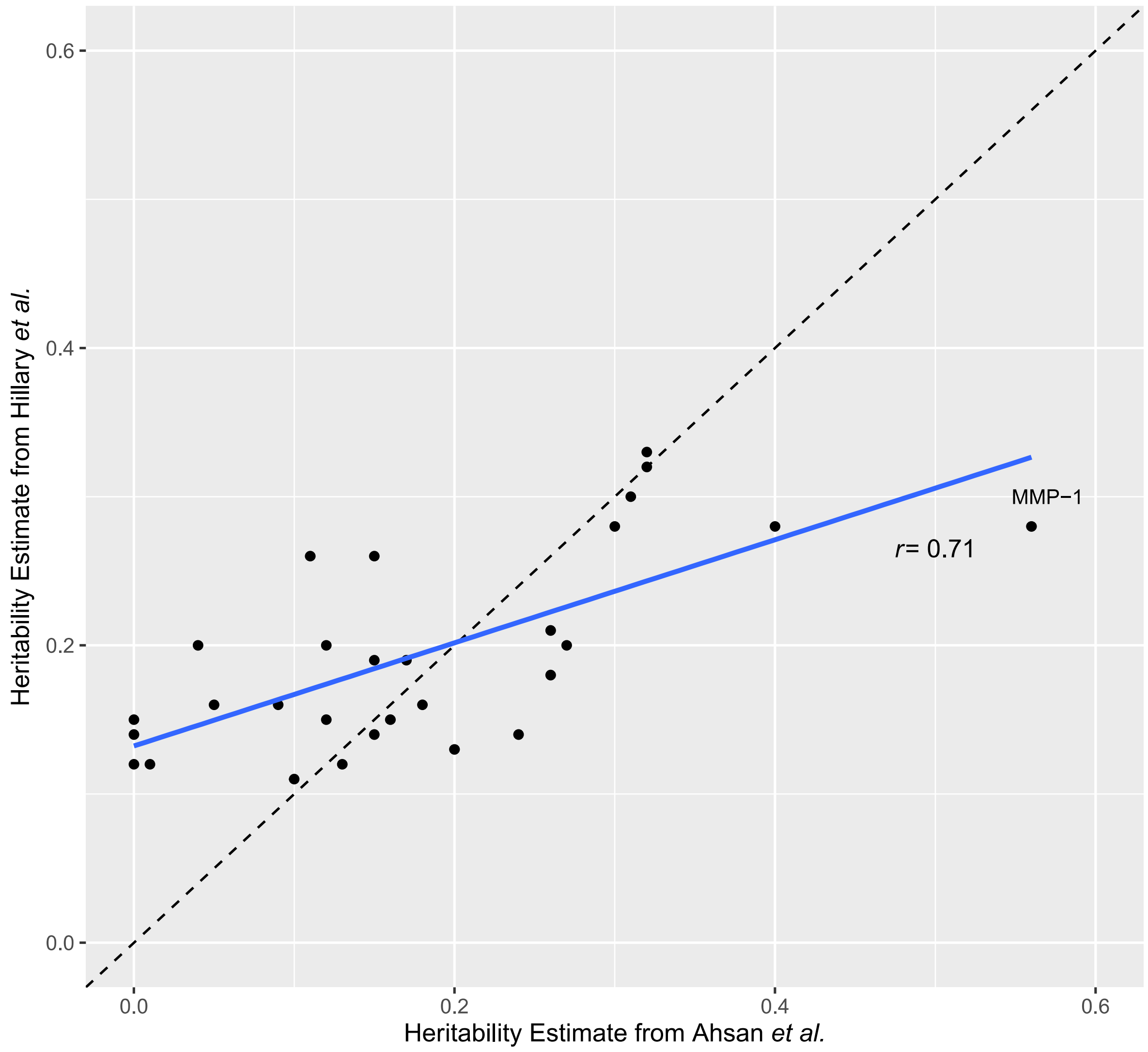

Fig. S2. Correlation between heritability estimates for circulating inflammatory protein biomarkers from present study and that of Ahsan *et al.* The protein with the greatest discordance between studies (MMP-1) is annotated.

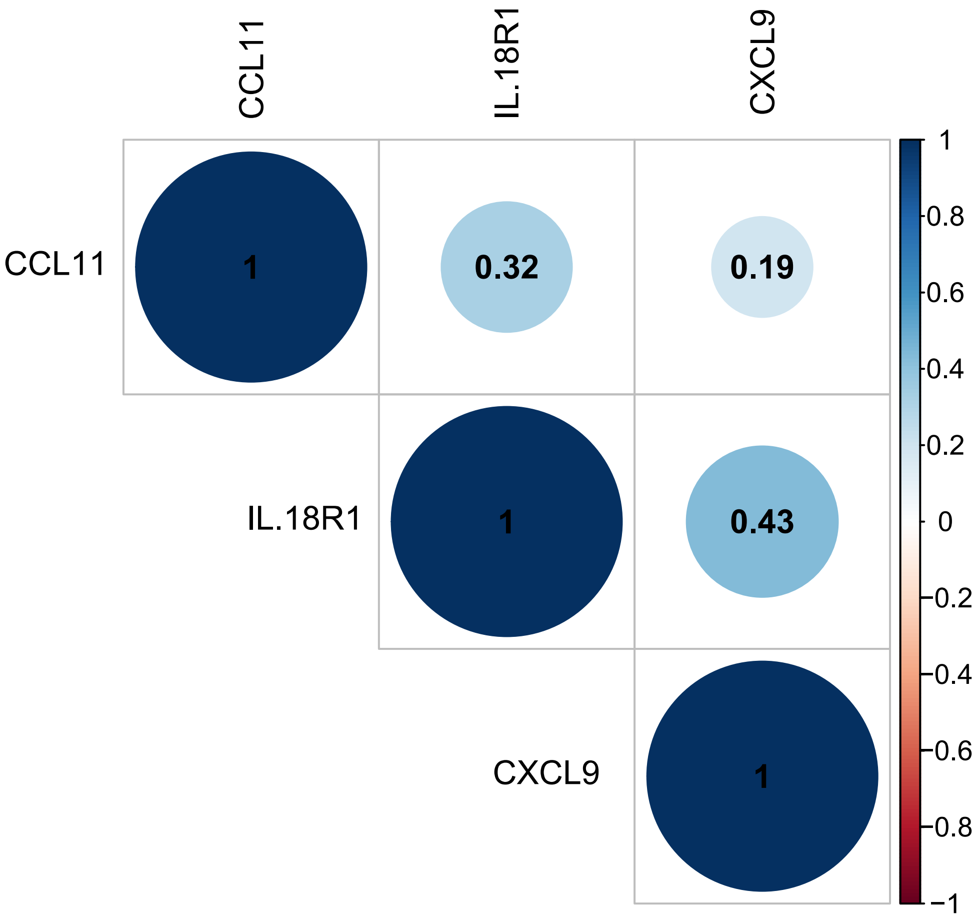

Fig. S3. Correlation between the 3 proteins with significant CpG associations as identified across ordinary least squares model, mixed model and Bayesian penalised regression approaches.

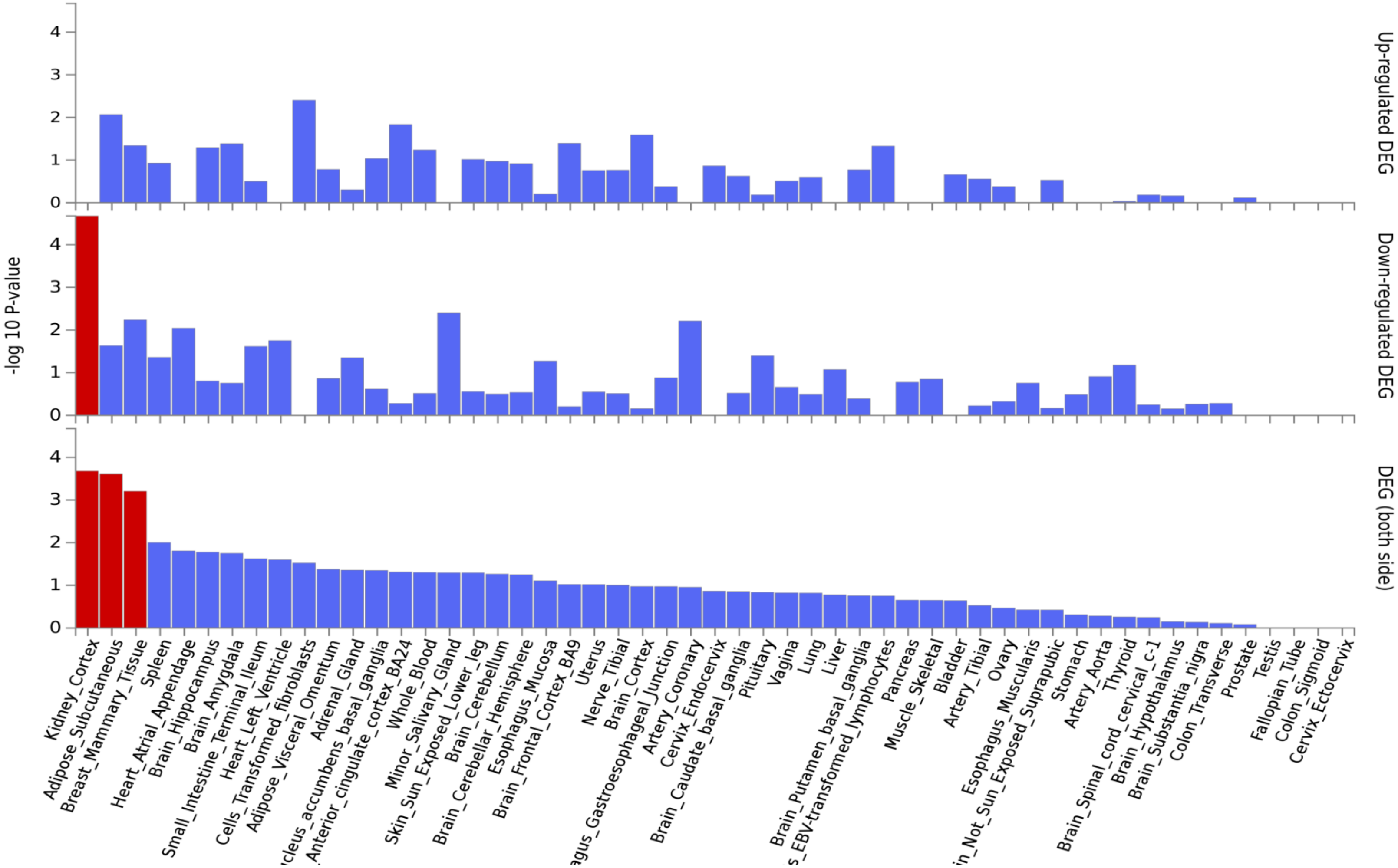

Fig. S4. Tissue-specific expression of genes annotated to CpGs associated with CCL11 levels at P < 1 x 10^{-5}. Differential expression was observed in kidney, adipose and breast tissue.

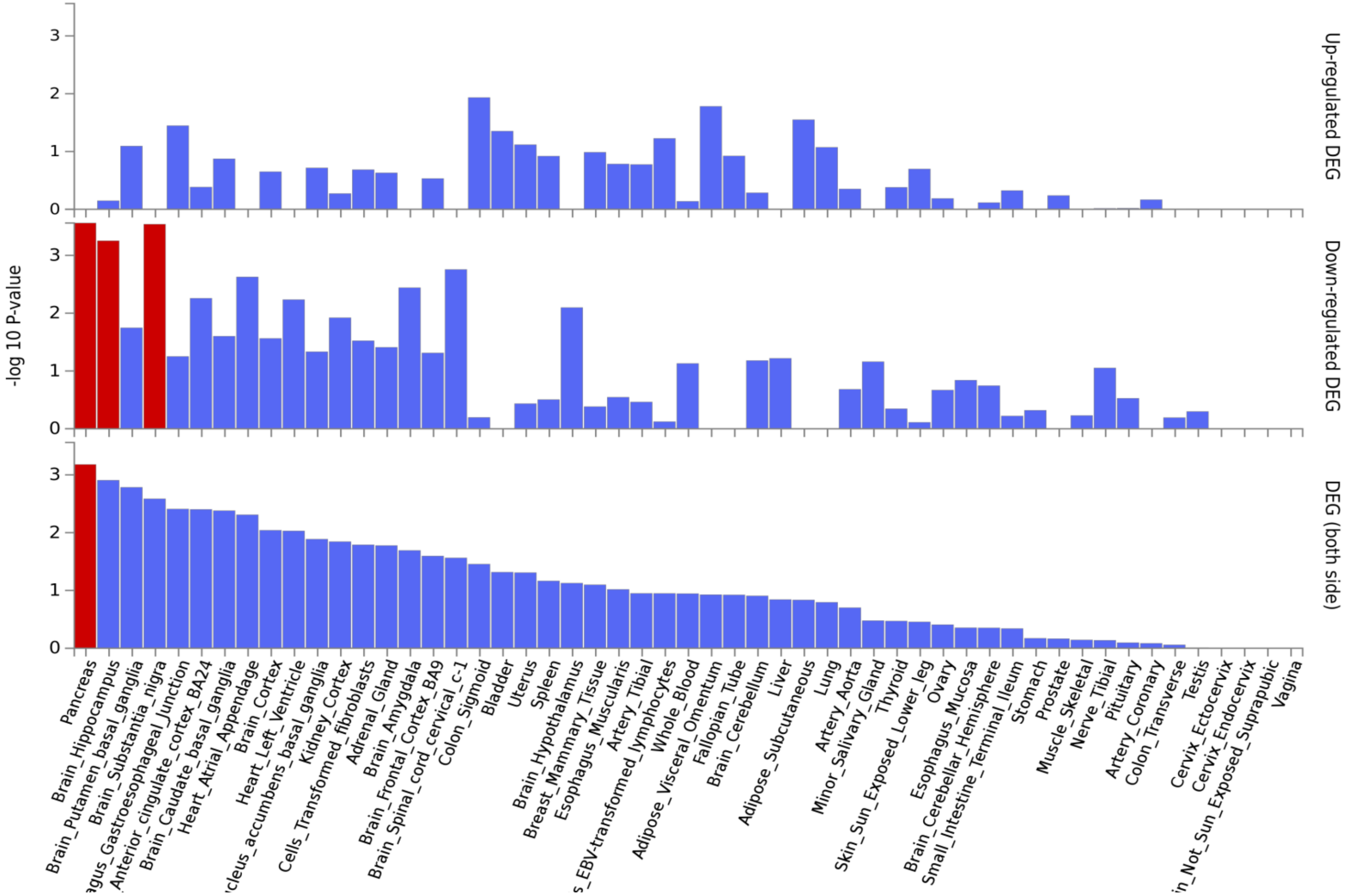

Fig. S5. Tissue-specific expression of genes annotated to CpGs associated with IL18R1 levels at $P < 1 \times 10^{-5}$. Differential expression was observed in pancreatic, hippocampal and substantia nigra tissue.

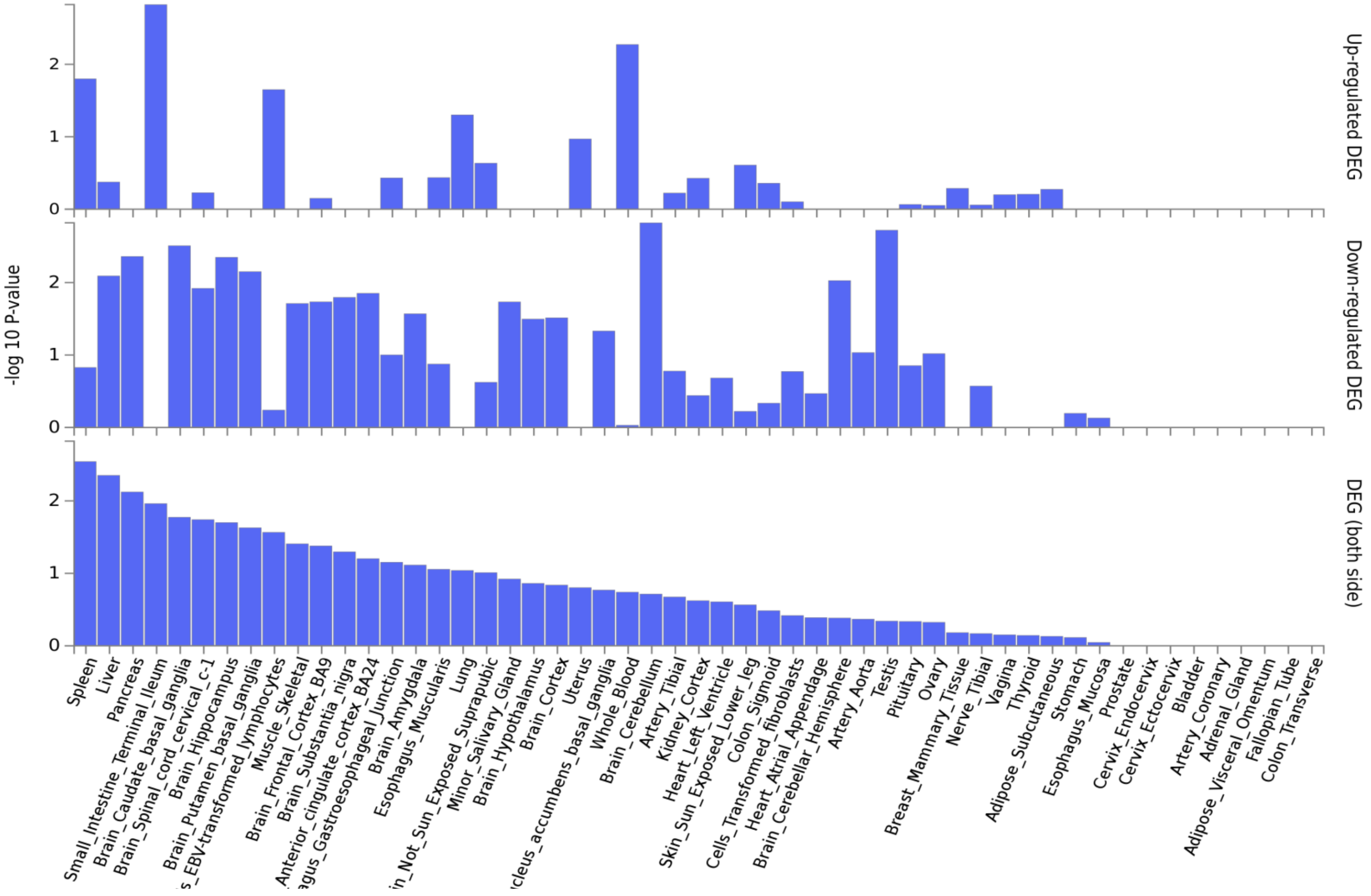

Fig. S6. Tissue-specific expression of genes annotated to CpGs associated with CXCL9 levels at $P < 1 \times 10^{-5}$. No tissue-specific expression was observed.

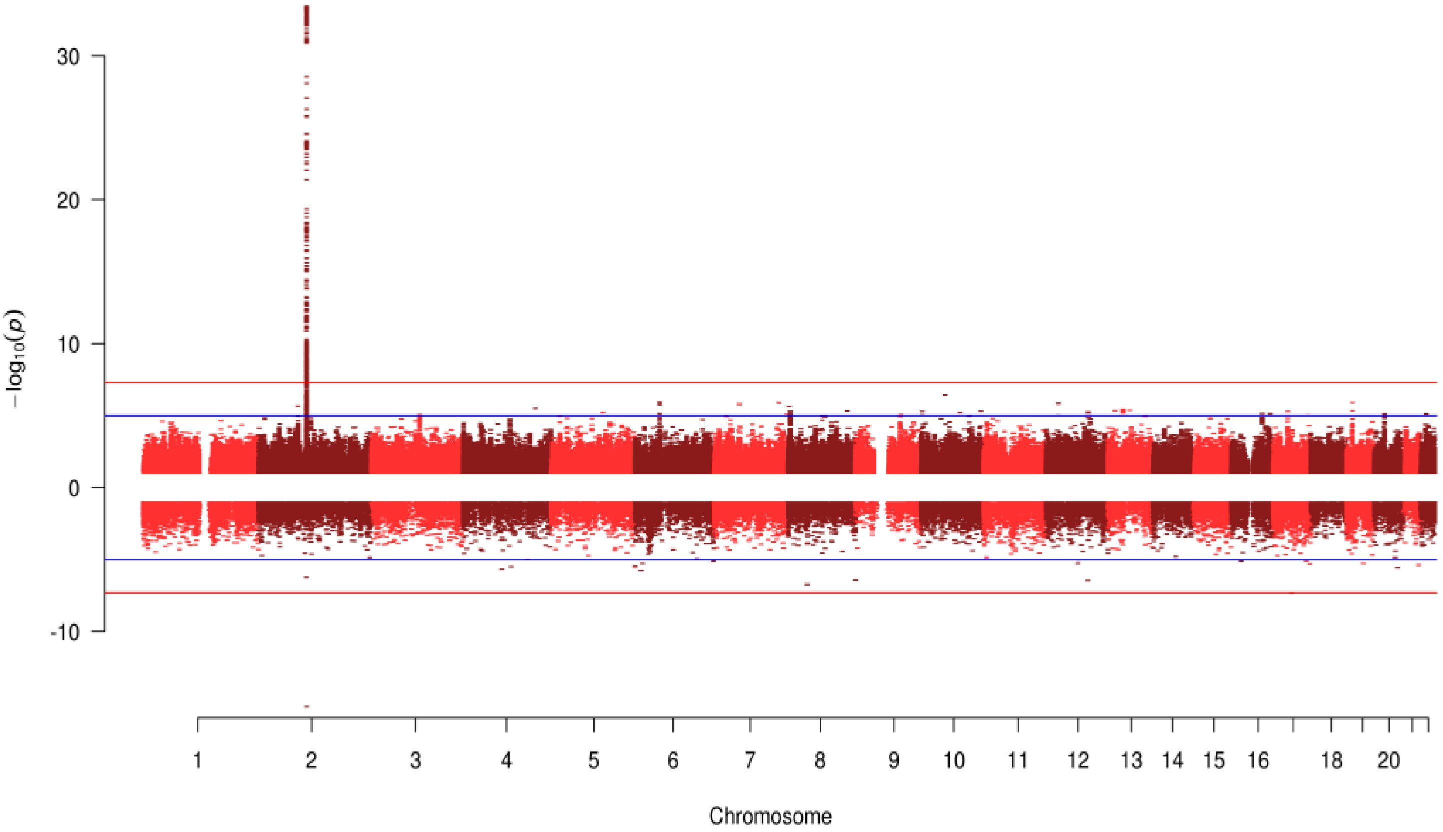

Fig. S7. Miami plot for IL18R1 which exhibited both genome-wide significant SNP and genome-wide significant CpG associations. The top half of the plot (skyline) shows the results from the GWAS on protein levels, whereas the bottom half (waterfront) shows the results from the EWAS. IL18R1 (chromosome 2: 102,311,529-102,398,775).
