# Supplementary figures and images for: Multi-method genome- and epigenome-wide studies of inflammatory protein levels in healthy older adults
Source: Genome Med. 2020 Jul 8;12:60. doi: 10.1186/s13073-020-00754-1 (PMC7346642; doi:10.1186/s13073-020-00754-1)

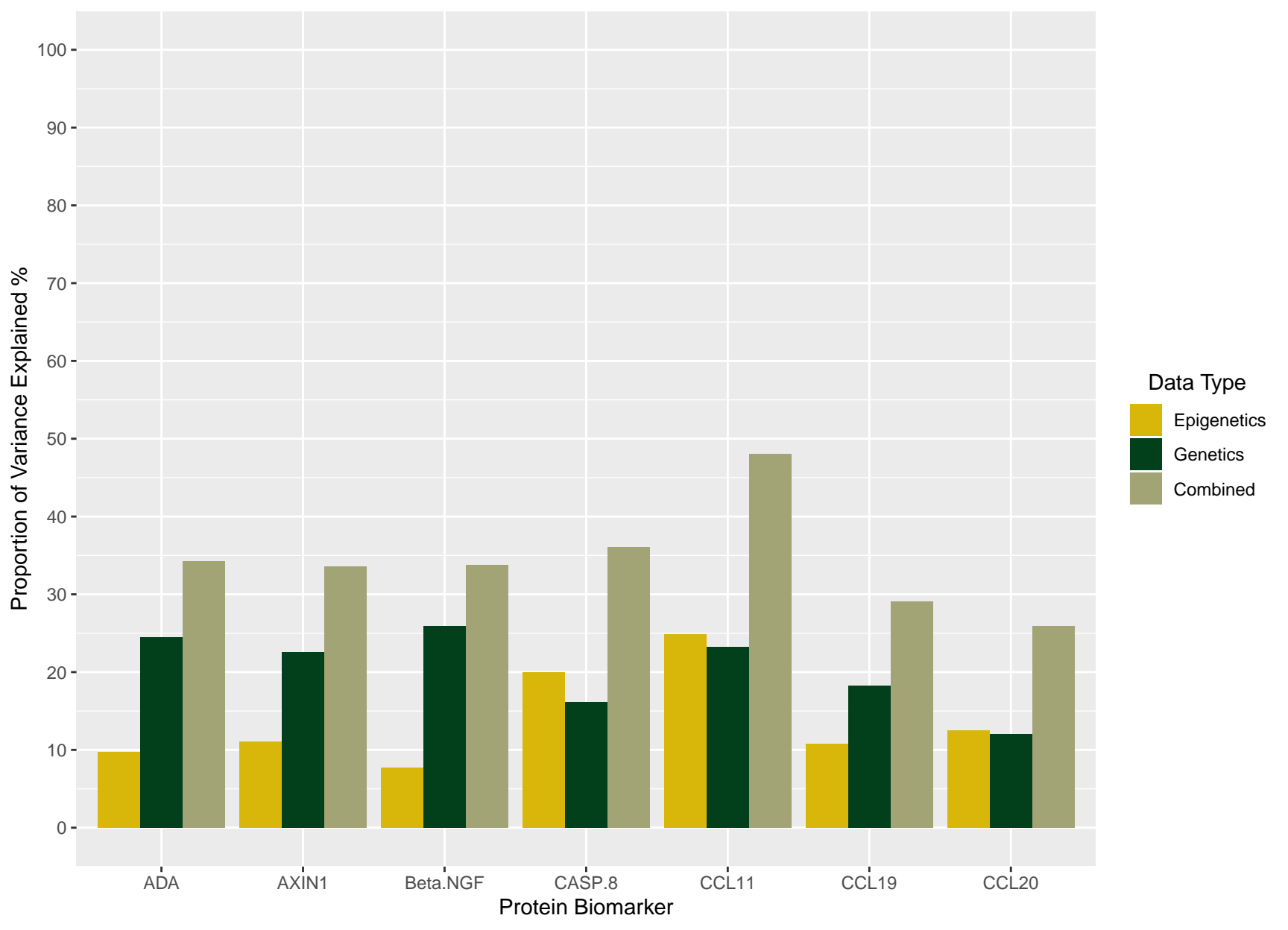

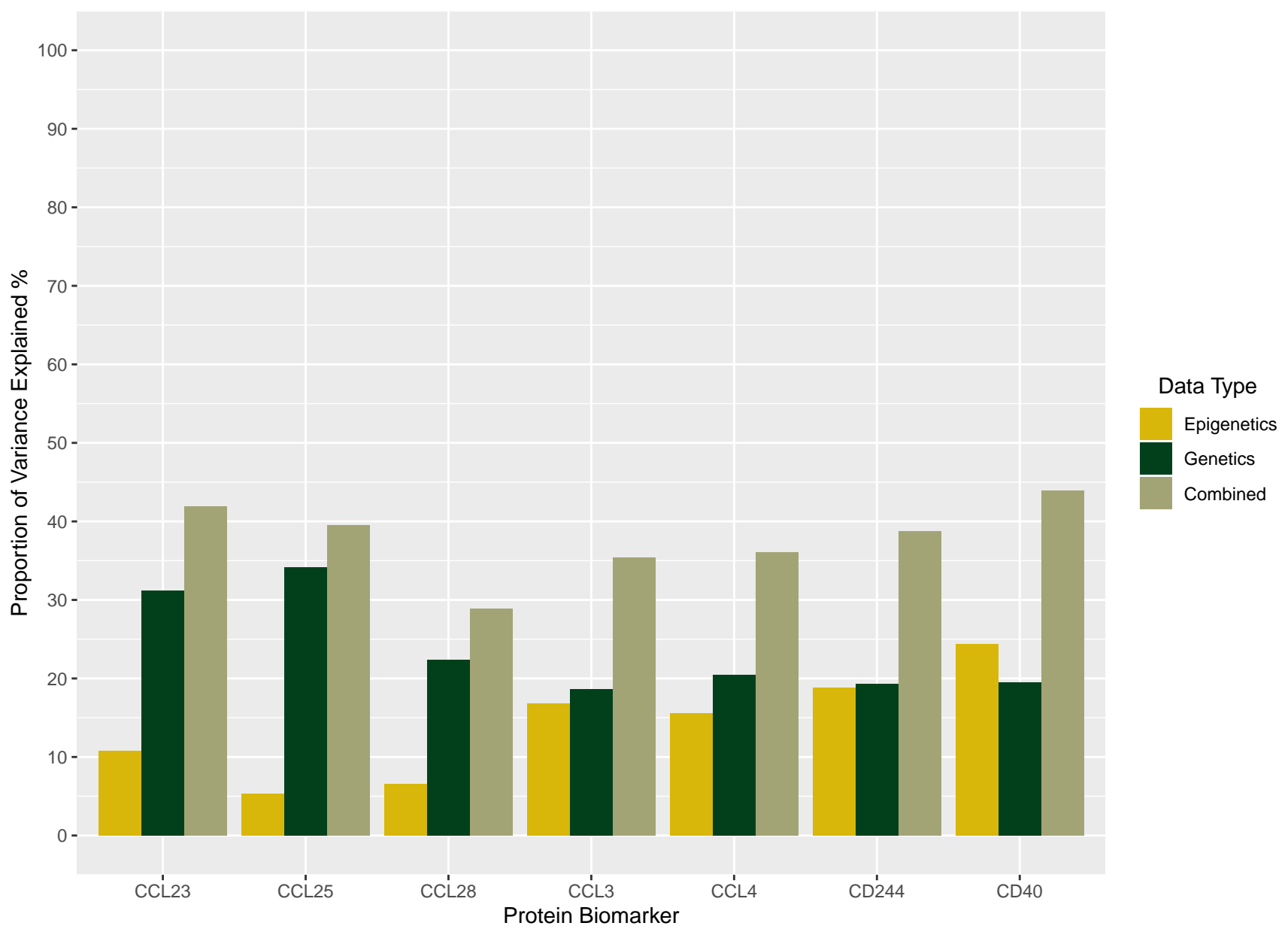

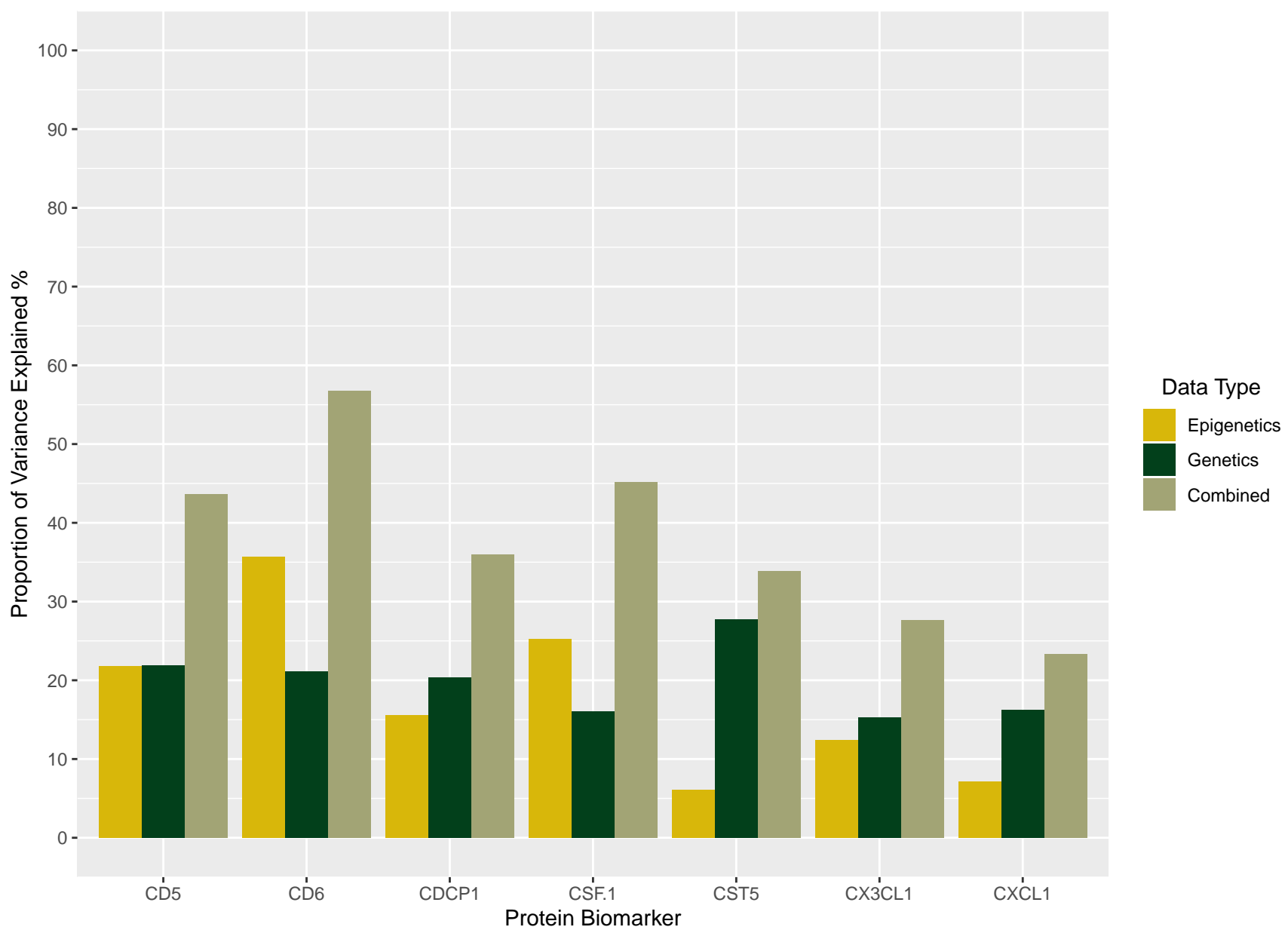

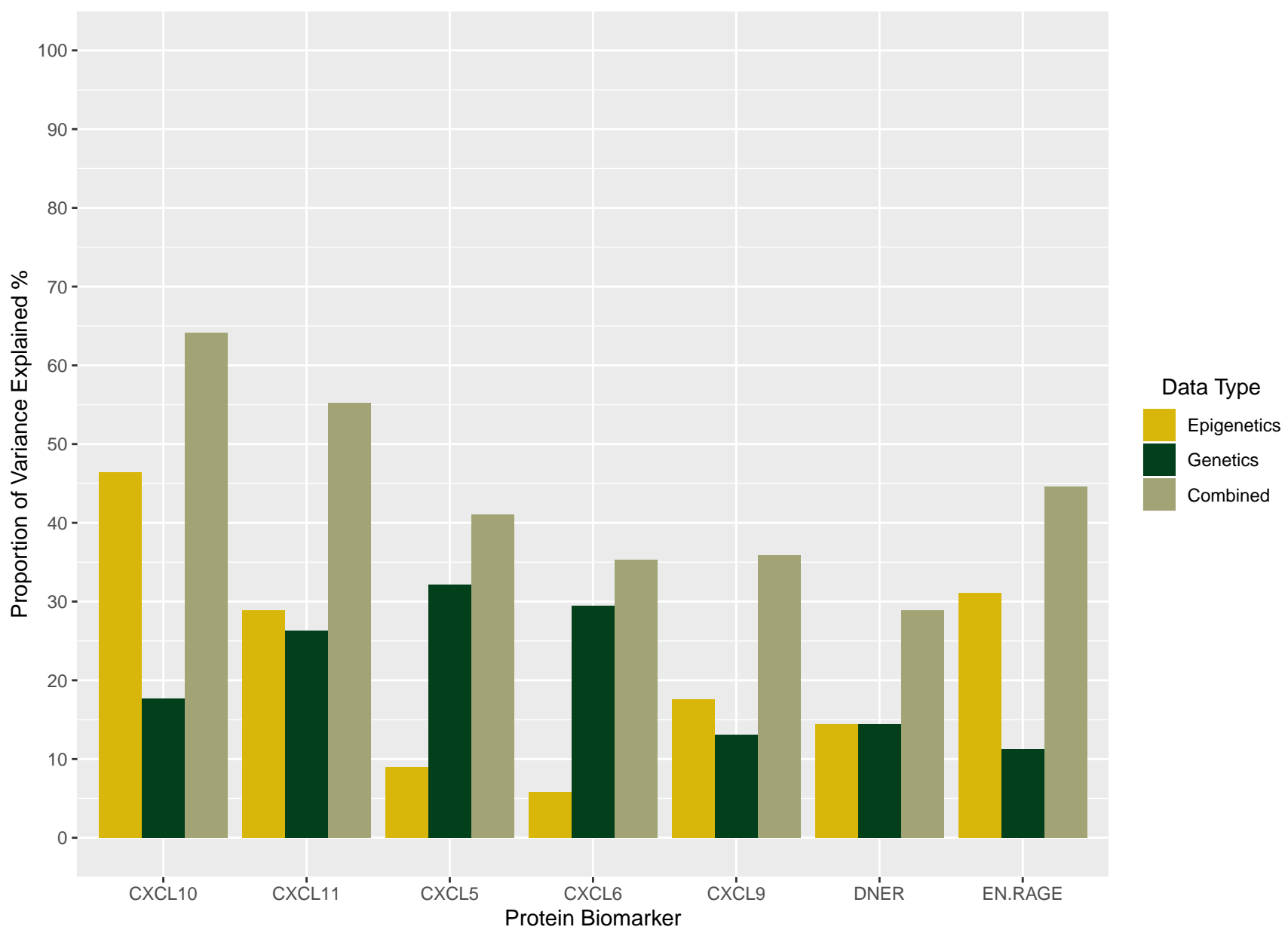

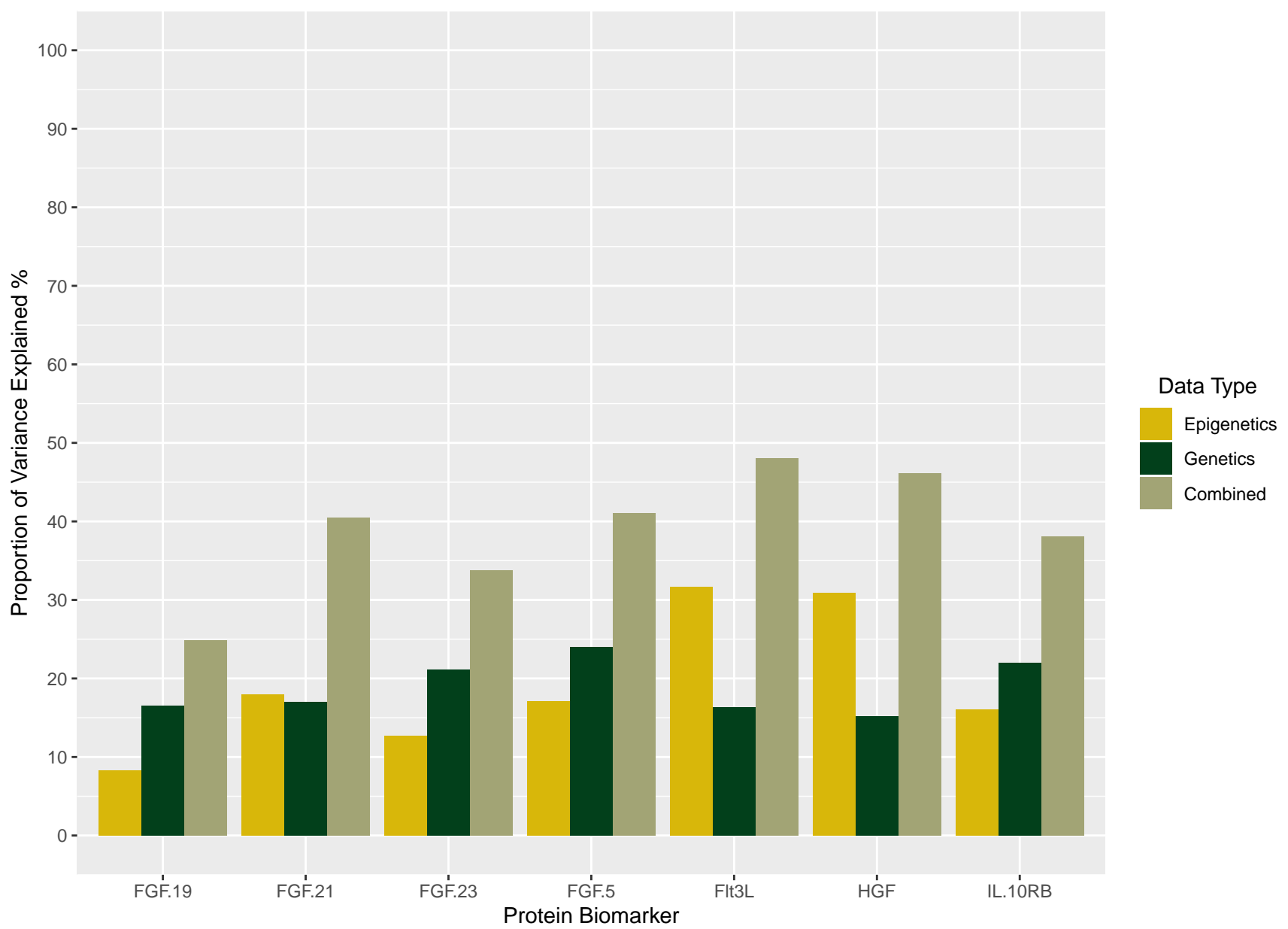

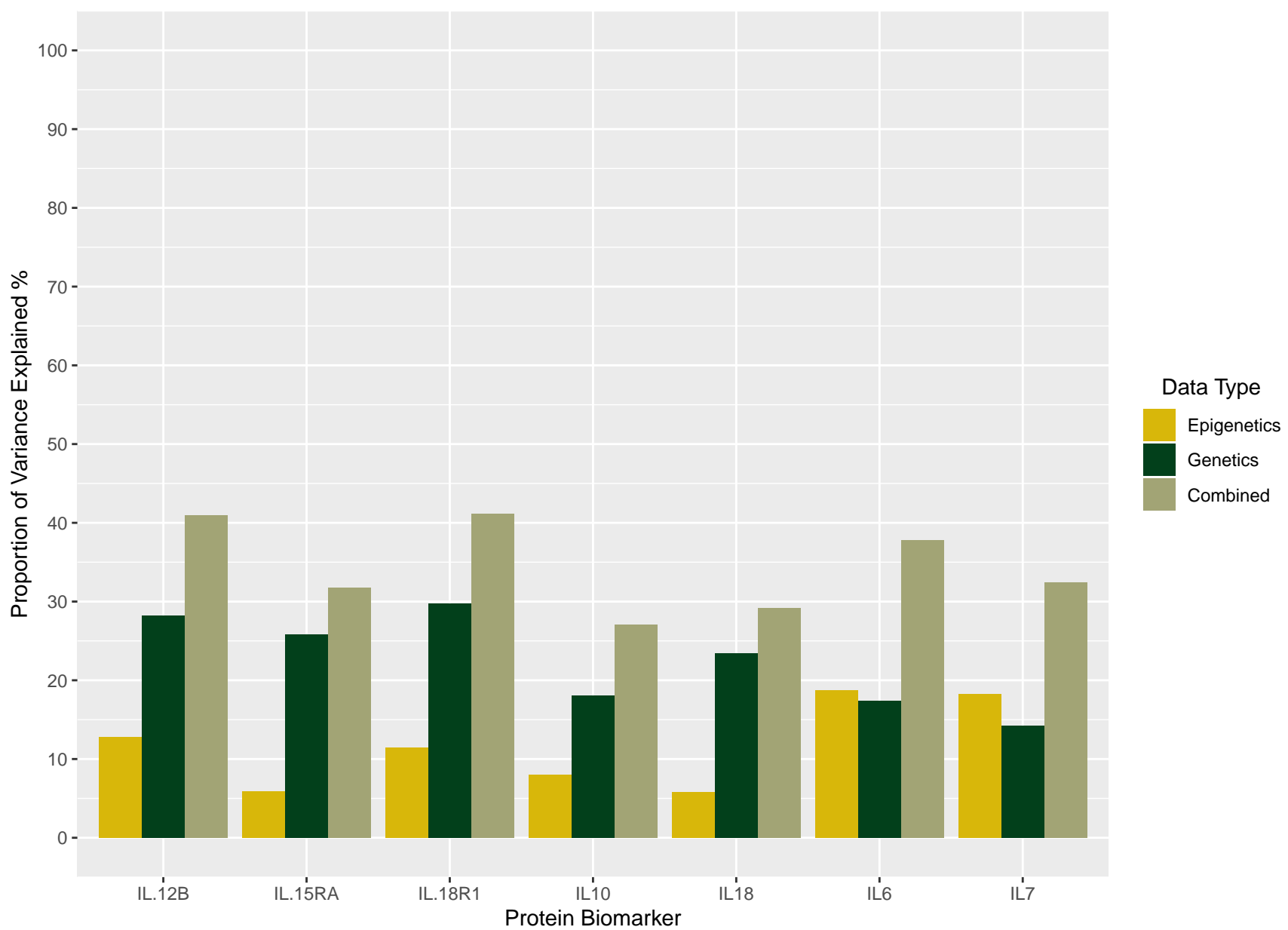

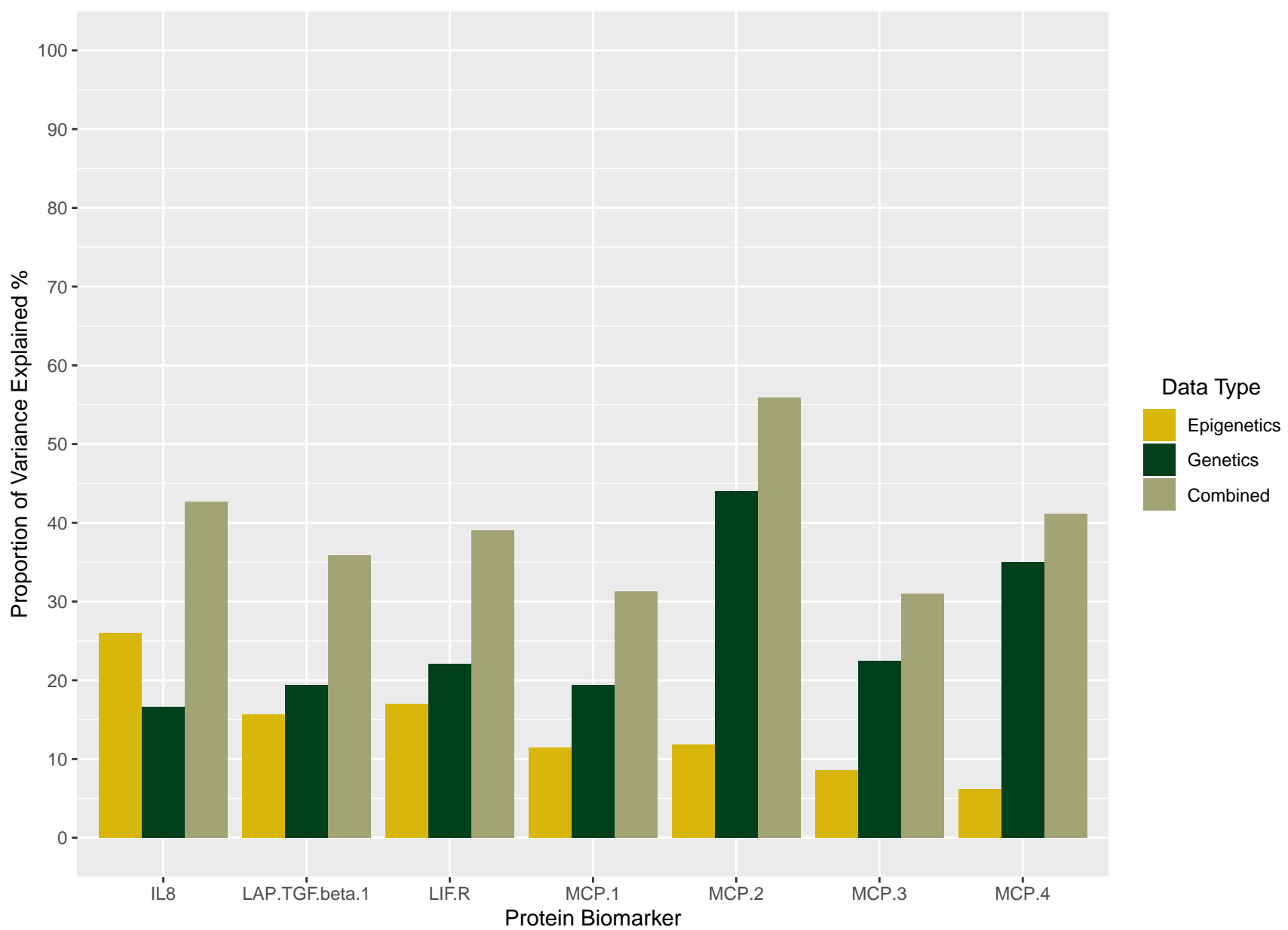

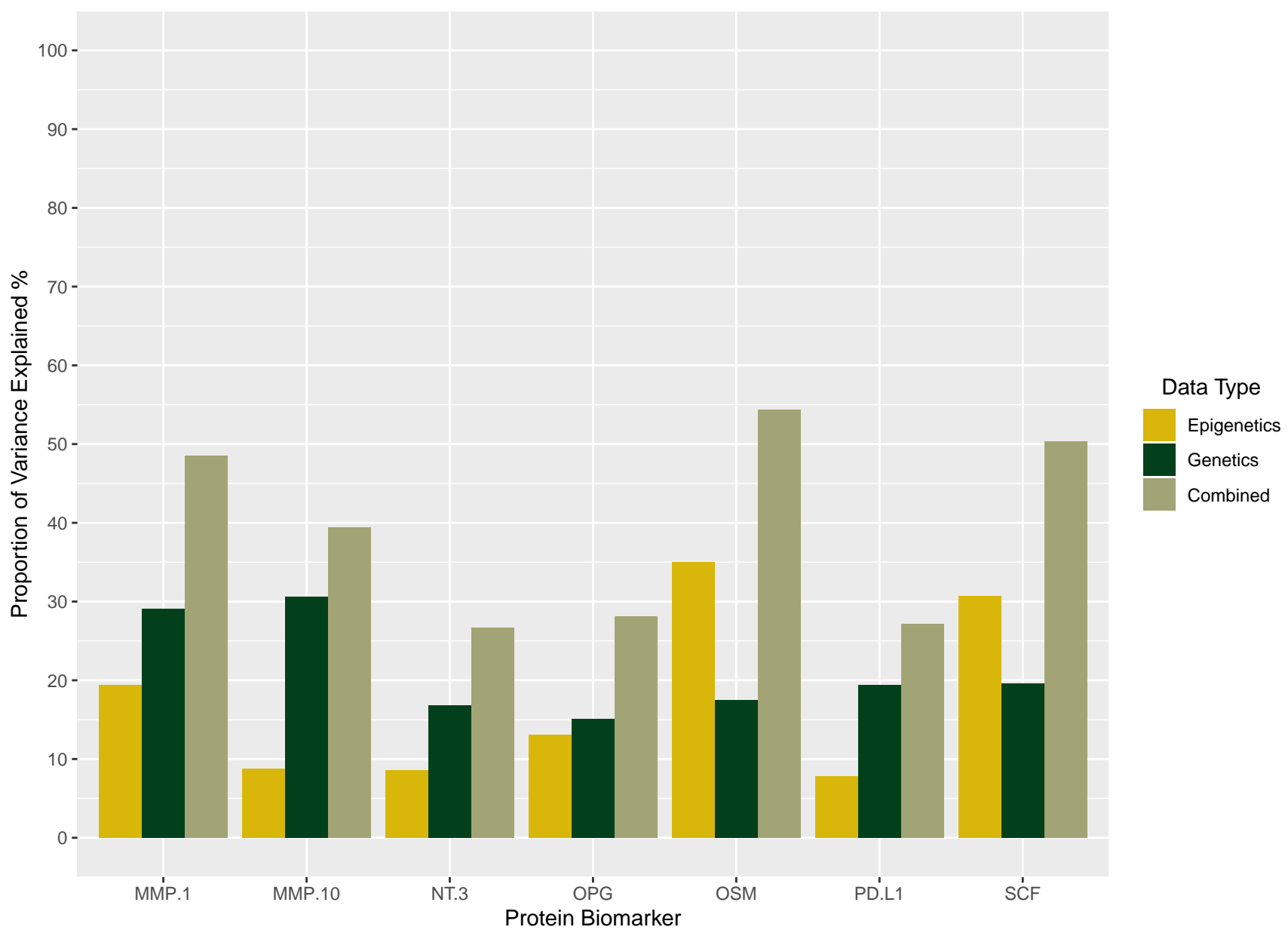

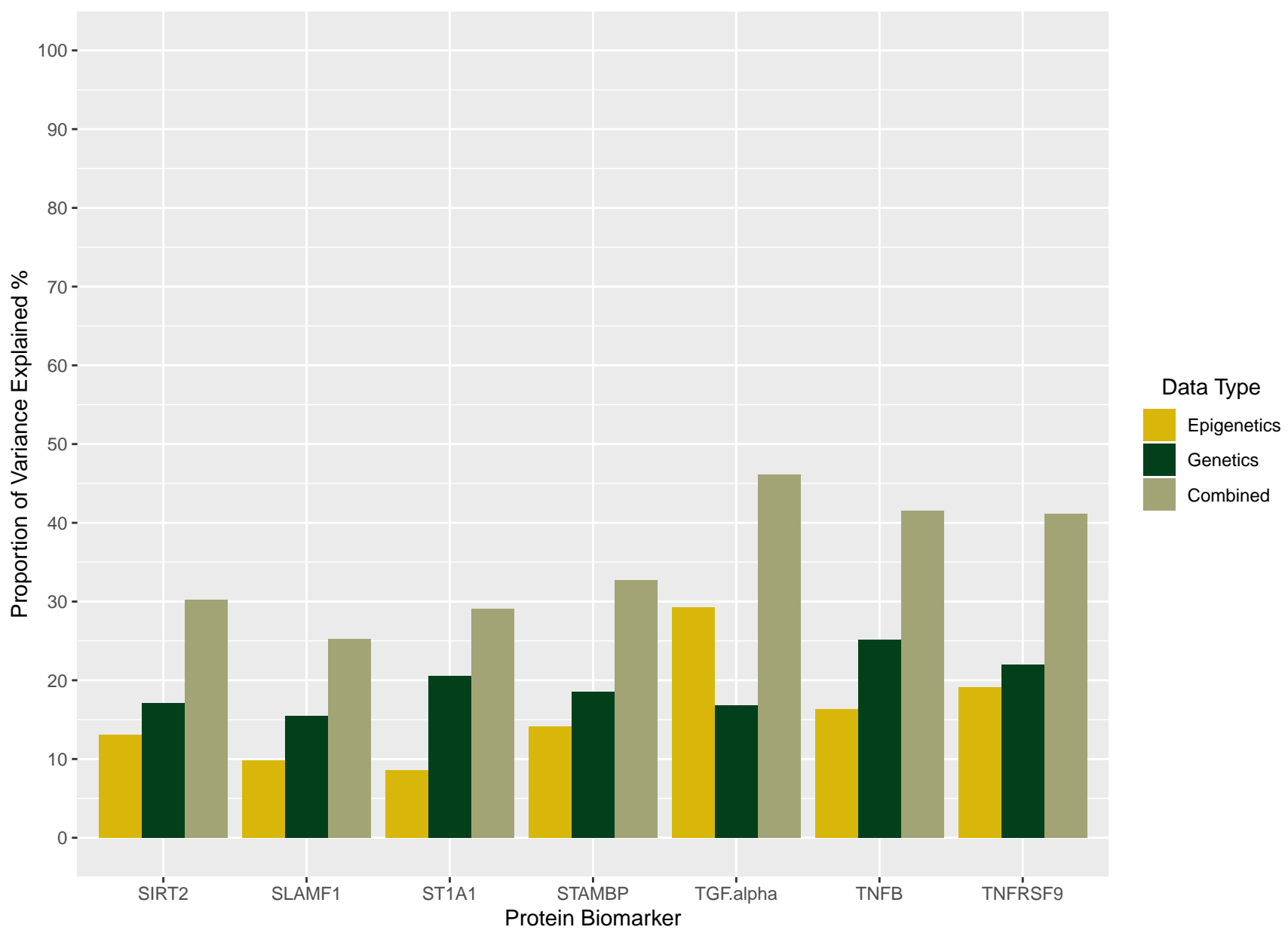

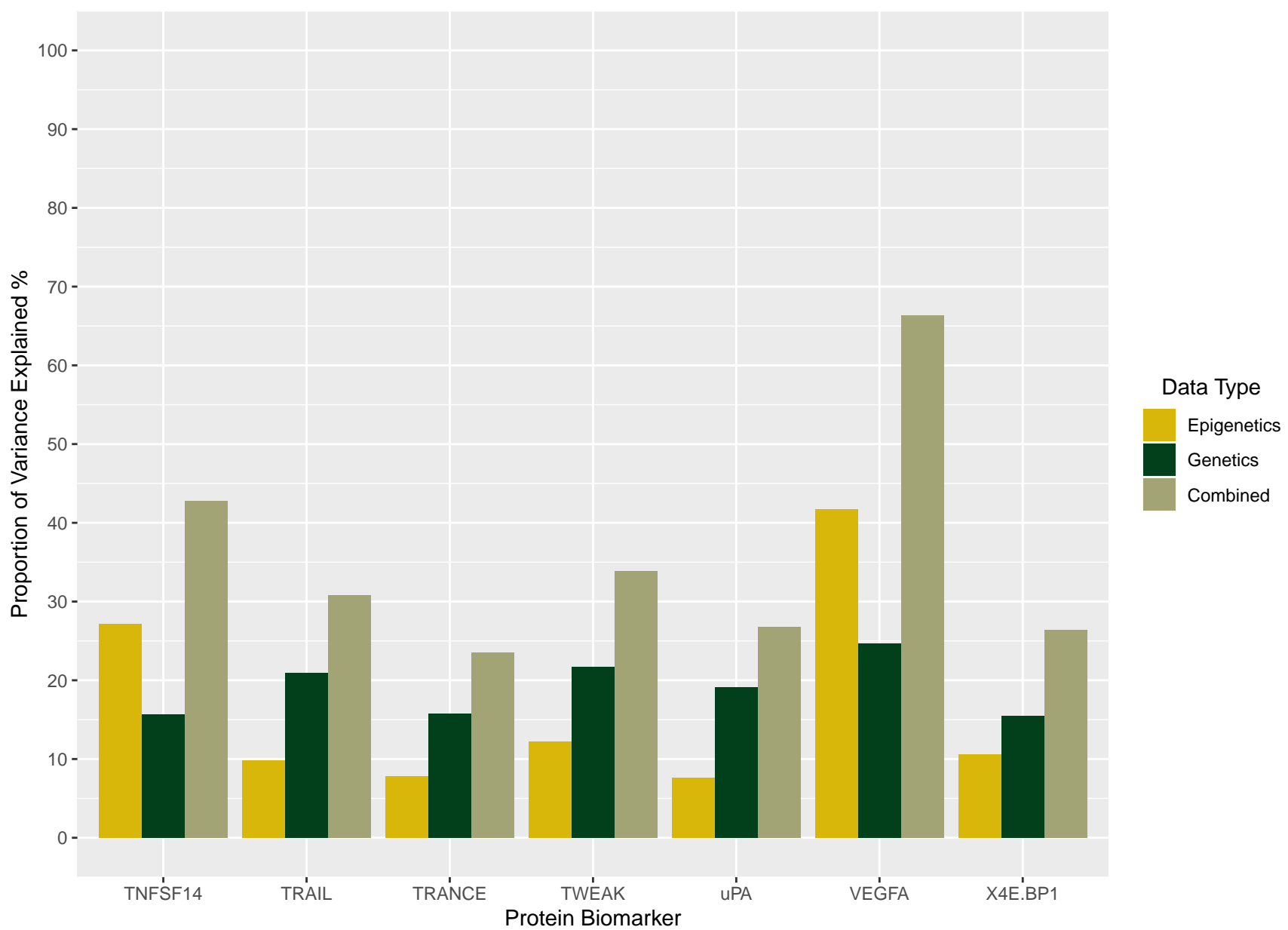

Supplement: Supplementary file 5 — Additional file 5. Variance in circulating protein levels explained by common genetic and methylation data together. [file 13073_2020_754_MOESM5_ESM.pdf]
